# Synthesis of disulfides and 3-sulfenylchromones from sodium sulfinates catalyzed by TBAI

**DOI:** 10.3762/bjoc.21.17

**Published:** 2025-02-03

**Authors:** Zhenlei Zhang, Ying Wang, Xingxing Pan, Manqi Zhang, Wei Zhao, Meng Li, Hao Zhang

**Affiliations:** 1 School of Chemistry and Material Engineering, Anhui Provincial Key Laboratory of Green Carbon Chemistry, Engineering Research Center of Biomass Conversion and Pollution Prevention of Anhui Educational Institutions, Biomass-derived Functional Oligosaccharides Engineering Technology Research Center of Anhui Province, Fuyang Normal University, Fuyang, Anhui, 236037, P. R. China

**Keywords:** disulfides, sodium sulfinates, 3-sulfenylchromones, TBAI, thiosulfonates

## Abstract

A new synthetic method for disulfides and 3-sulfenylchromones is reported. This innovative approach is based on the tetrabutylammonium iodide (TBAI)/H_2_SO_4_ reduction system using sodium sulfinate as key component, thus eliminating the need for thiols and redox reagents commonly used in traditional methods. Various disulfides and 3-sulfenylchromones were obtained in moderate to excellent yields through this methodology. Mechanistic studies indicate that thiosulfonates play an important role in the reaction process.

## Introduction

Organosulfur compounds containing S–S bonds, often referred to as disulfides, are among the most valuable functional groups in organic synthesis [1–4]. In chemistry and biology, disulfide bonds play crucial roles in protein folding and stabilization [5–8] and in the rubber industry, they are used to link different polymer chains [9–10]. The disulfide bond backbone is commonly used as a linker for antibody–drug coupling (ADCs), in which the active drug released in the target cell by selectively breaking the disulfide bond [11]. Given the wide applicability of disulfides, the development of efficient, green, mild, and cost-effective methods for the organic synthesis of disulfides is of significant importance.

**Scheme 1:**
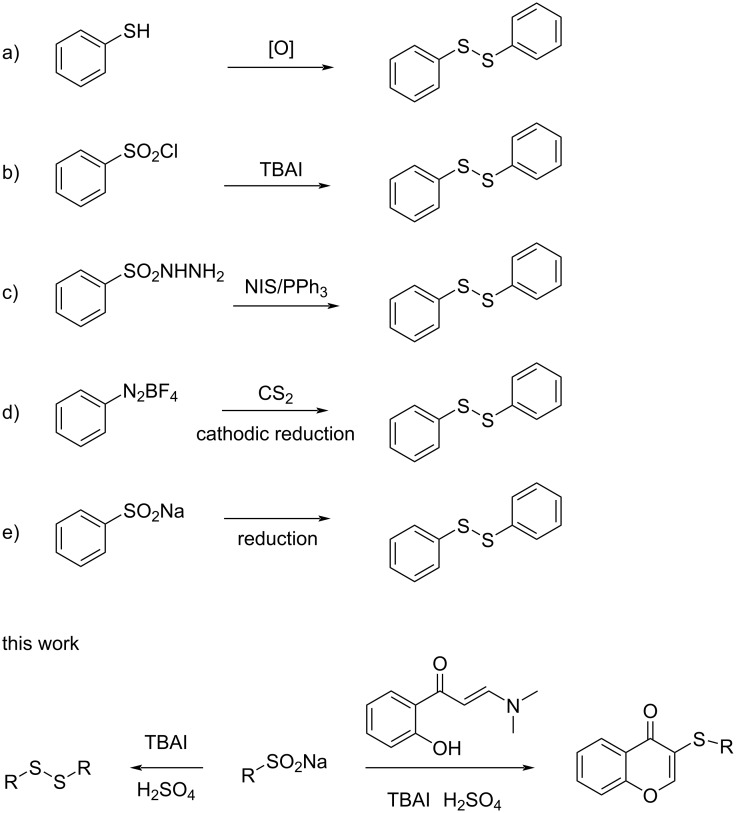
Different strategies for the synthesis of disulfides and 3-sulfenylchromones.

In the field of disulfide synthesis, the conventional approach mainly involves oxidizing thiols to form disulfides. This process employs various oxidation agents, which can be categorized as nonmetallic and metal derivatives [12–16]. Over the past few years, the implementation of catalysts in conjunction with oxygen [17–22], electrochemical oxidation [23–24], and photochemical oxidation techniques [25] have emerged as alternative methods. However, these approaches have a significant limitation: the substrates must be thiols, which have unpleasant odors. This has prevented their widespread use on a large scale. Recently, research efforts have focused on exploring alternative reagents that offer the advantages of being odorless and more stable than thiols. These alternatives include sulfonyl chloride [26], sulfonyl hydrazine [27], carbon disulfide [28], and sodium sulfinate (Scheme 1) [29–32]. Among the available alternatives, sodium sulfinate is particularly interesting because it is more stable and easier to transport, and it is widely used in organic synthesis [33–37]. When using sodium sulfite as the starting material for the construction of disulfides, it is typically necessary to introduce equivalent reducing agents, such as PPh_3_ [29], HI [30], HPO(OEt)_2_ [31] or iron powder [32] into the reaction mixture. Although there have been numerous studies on the synthesis of disulfides from sodium sulfinate, the development of a method to synthesize disulfides from sodium sulfinate without the use of additional redox reagents remains a challenging task.

3-Sulfenylchromones are an important class of heterocyclic compounds with unique skeletons in nature that play an essential role in drug synthesis and development. To synthesize these compounds, direct C(sp^2^)–H radical sulfenylation of enaminones or chromones has emerged as a popular strategy using various sulfenyl precursors such as thiophenols [38–40], sulfonyl hydrazides [41–42], disulfides [43], KSCN [44], S_8_ [45], methylsulfinyl derivatives [46], sodium sulfinates [47], and thiosulfonates [48–49]. However, some of the previously reported methods have limitations such as the use of strong oxidants, expensive reagents/catalysts, and lengthy work-up procedures, so there is still a need for simpler and more environmentally friendly methods for the preparation of 3-sulfenylchromones.

In this study, we report the synthesis of corresponding disulfides under the catalysis of TBAI (tetrabutylammonium iodide) using sodium alkyl or aromatic sulfinates as sulfur sources. Sodium sulfinates are more stable than sulfonyl hydrazides, sulfonyl chlorides, and thiols, and there is no need to add additional redox reagents to the reaction, making this method a valuable addition to the synthesis of disulfides. Furthermore, this approach can be extended to the synthesis 3-sulfenylchromones, thereby broadening its application.

## Results and Discussion

Reaction conditions were optimized using sodium *p*-toluenesulfinate as the model substrate and tetrabutylammonium iodide (TBAI) as the catalyst, the results are listed in Table 1. Various acids were tested to assess their effect on the reaction (Table 1, entries 1–5). From the results, it could be concluded that, in the presence of strong acids, all afforded the product in moderate yields, with sulfuric acid being the most effective. It is important to note that sulfuric acid is a dibasic acid. Subsequently, when the amount of HCl was increased to 2 equivalents, a significant increase in yield was observed (Table 1, entry 5). Weak acids such as acetic acid did not give the product, suggesting that conversion of sodium 4-methylbenzenesulfinate to 4-methylbenzenesulfinic acid was required. Several solvents were tested and found to produce the disulfide in low yield (Table 1, entries 6–11), except for dimethylacetamide (DMA). Given the dimerization nature of the reaction, an attempt was made to increase the concentration, reducing the amount of solvent to 1 mL (Table 1, entry 12), product **2a** was obtained in 67%, and a further reduction to 0.5 mL failed to increase the yield significantly (Table 1, entry 13). Although the amount of solvent used could have been further decreased, in the end, 0.5 mL of DMF was used because of the solubility of the reagents. Increasing the amount of catalyst to 20 mol % resulted in a significant increase in the yield, but further increases did not significantly change the yield (Table 1, entries 14 and 15). A decrease in reaction temperature resulted in a significant decrease in the yield (Table 1, entry 16) and at 80 °C no desired product, but only thiosulfonate was formed (Table 1, entry 17). The reaction did not proceed in the absence of acid or catalyst (Table 1, entries 18 and 19). Compared to TBAI, other iodized salts gave relatively lower yields (Table 1, entries 20 and 21). The replacement of the catalyst with tetrabutylammonium bromide (TBAB) gave no product (Table 1, entry 22). Finally, sulfuric acid as the acidifying agent, tetrabutylammonium iodide as the catalyst, DMF as the solvent and carrying out the reaction at 120 °C for 2 hours were determined as optimal conditions for the reaction (Table 1, entry 14).

**Table 1:** Optimization of reaction conditions.^a^

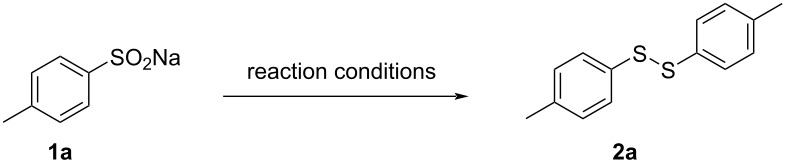					

Entry	Solvent	Acid	Catalyst	Temperature (°C)	Yield (%)^b^

1	DMF	H_2_SO_4_	TBAI	120	48
2	DMF	TFA	TBAI	120	31
3	DMF	HCl	TBAI	120	33
4	DMF	HOAc	TBAI	120	0
5^c^	DMF	HCl	TBAI	120	46
6	DMSO	H_2_SO_4_	TBAI	120	0
7	toluene	H_2_SO_4_	TBAI	120	24
8	dioxane	H_2_SO_4_	TBAI	120	0
9	DMA	H_2_SO_4_	TBAI	120	41
10	H_2_O	H_2_SO_4_	TBAI	120	11
11	THF	H_2_SO_4_	TBAI	120	9
12^d^	DMF	H_2_SO_4_	TBAI	120	67
13^e^	DMF	H_2_SO_4_	TBAI	120	68
**14** ^e,f^	**DMF**	**H** **_2_** **SO** **_4_**	**TBAI**	**120**	**80**
15^e,g^	DMF	H_2_SO_4_	TBAI	120	77
16^e,f^	DMF	H_2_SO_4_	TBAI	100	60
17^e,f^	DMF	H_2_SO_4_	TBAI	80	0
18^e^	DMF	H_2_SO_4_	–	120	0
19^e,f^	DMF	–	TBAI	120	0
20^e,f^	DMF	H_2_SO_4_	KI	120	70
21^e,f^	DMF	H_2_SO_4_	NH_4_I	120	72
22^e,f^	DMF	H_2_SO_4_	TBAB	120	0

^a^Reaction conditions: **1a** (1 mmol), acid (1 mmol), catalyst (0.1 mmol), solvent (2 mL), 120 °C for 2 h, under air. ^b^Isolated yield. ^c^Acid (2 mmol). ^d^DMF (1 mL). ^e^DMF (0.5 mL). ^f^Catalyst (0.2 mmol). ^g^TBAI (0.3 mmol).

**Scheme 2:**
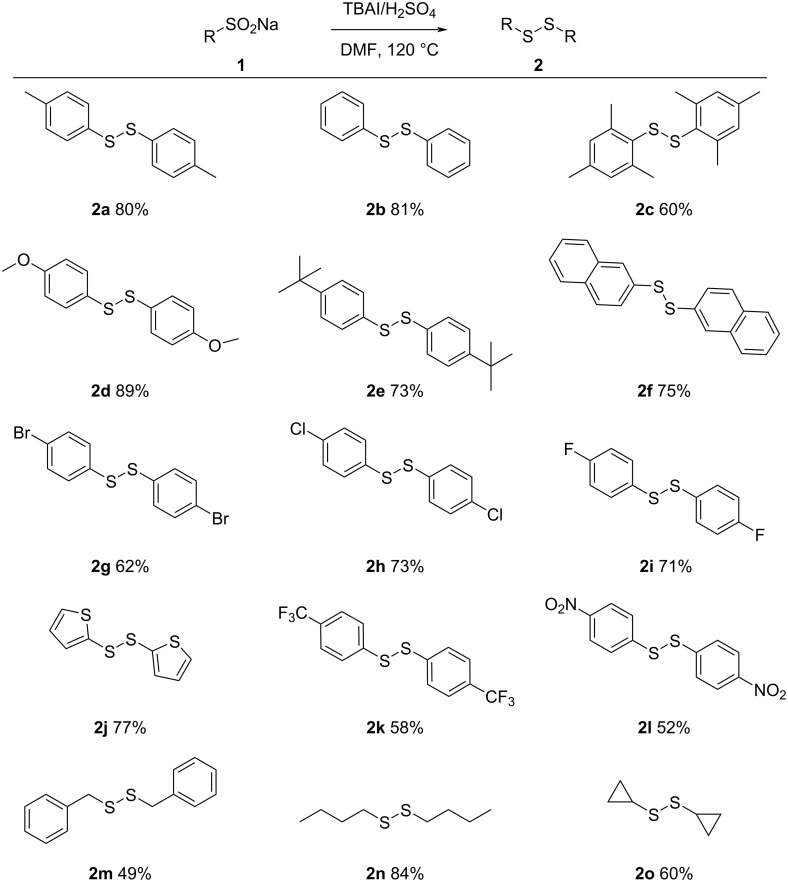
Substrate scope for the synthesis of disulfides. Reaction conditions: **1** (1 mmol), TBAI (0.2 mmol), H_2_SO_4_ (1 mmol), and DMF (0.5 mL) at 120 °C for 2 h.

Under the optimized conditions, the coupling reaction of various sodium arylsulfinates was investigated to assess the scope and generality of this protocol. The results, shown in Scheme 2, demonstrated that a wide range of disulfides were synthesized efficiently when sodium sulfinates served as substrates. The isolated yields ranged from 49% to 89% for products **2a**–**o**. In the case of sodium arylsulfinates with various substituents on the benzene ring, we observed minimal effects on the reaction. For example, electron-donating groups (Me, *tert*-butyl, MeO, naphthyl) and electron-withdrawing groups (F, Cl, Br) attached to the benzene ring led to products **2a**–**i** with yields ranging from 60% to 89%. Interestingly, products with strong electron-withdrawing substituents were obtained in moderate yields (**2k** and **2l**). Additionally, when a heterocyclic compound was used as substrate (**2j**), the desired product was obtained in higher yield, and when sodium benzylsulfinate was used as substrate, the corresponding disulfide **2m** was obtained in moderate yield. Under these reaction conditions, sodium alkylsulfinates were also successfully converted to the corresponding disulfides in high yields (**2n** and **2o**).

In our synthesis of disulfides, thiosulfonates were also present as intermediates besides forming disulfides. In view of the fact that in the literature above disulfides or thiosulfonates could be converted with enaminones to 3-sulfenylchromones under iodine catalysis, an attempt was made to see whether this reaction system would be suitable for this reaction. Fortunately, the target products could indeed be obtained in high yields under these reaction conditions.

**Scheme 3:**
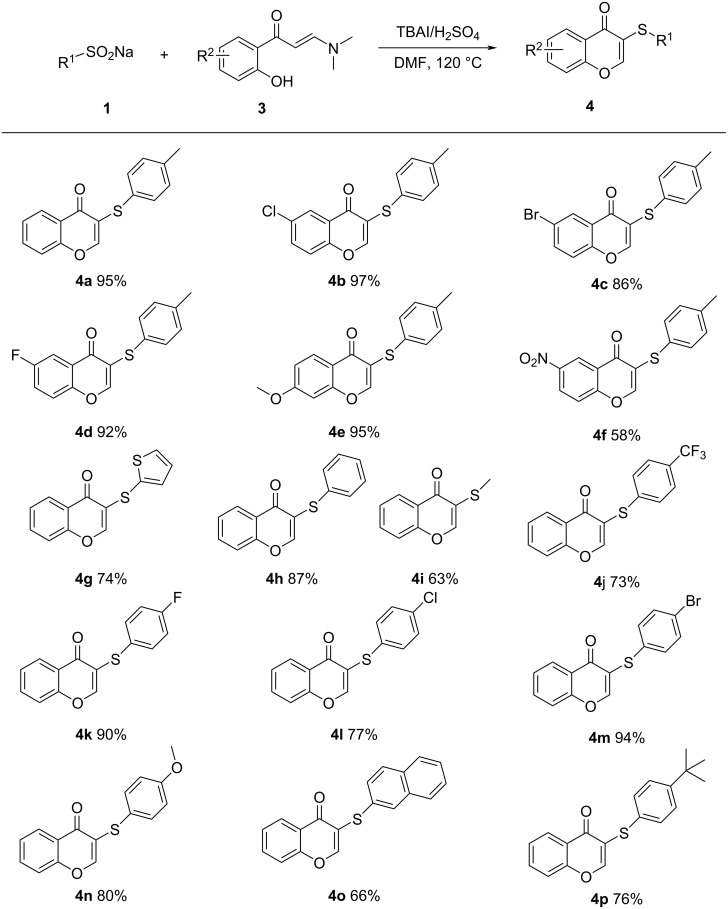
Substrate scope for the synthesis of 3-sulfenylchromones. Reaction conditions: **1** (1 mmol), **3** (0.5 mmol), TBAI (0.2 mmol), H_2_SO_4_ (1 mmol) and DMF (1 mL) at 120 °C for 12 h; yield based on **3**.

Based on the optimized conditions presented above, the substrate range of the TBAl-catalyzed sulfenylation/annulation reaction between different arylated enaminones and sodium *p*-tolylsulfinate was subsequently investigated, and the corresponding results are shown in Scheme 3. From the results, both electron-withdrawing and electron-donating groups on the aryl substituents of the enaminones were tolerated and afforded the corresponding products in good to excellent yields (**4a**–**f**), with a slight decrease in the yield if 5-nitrophenyl-substituted enaminone was used as a substrate (**4f**). Both sodium arylsulfinates and sodium alkylsulfinates (**4g**–**p**) gave more than moderate yields of the corresponding products.

**Scheme 4:**
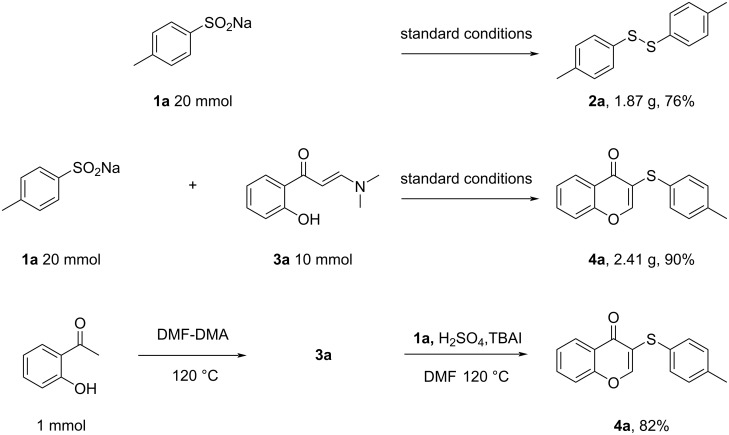
Gram-scale synthesis of **2a** and **4a** and one-pot synthesis of **4a**.

To evaluate the feasibility of the process in scale-up studies, gram-scale **2a** was synthesized under optimized conditions. The reactions of **1a** (1.760 g, 10 mmol) with H_2_SO_4_ (10 mmol) and TBAI (2 mmol) gave the corresponding 1,2-di-*p*-tolyldisulfane (**2a**, 1.87 g) in a yield of 76% (Scheme 4). A similar yield of 90% was obtained for compound **4a**.

To make the synthesis of 3-sulfenylchromones more convenient, the product was synthesized by a one-pot method using 2-hydroxyacetophenone as the starting material (Scheme 4). First, 2-hydroxyacetophenone and *N*,*N*-dimethylformamide dimethyl acetal (DMF-DMA) were reacted at 120 °C to give compound **3a**, and without further isolation and purification, **1a**, H_2_SO_4_ and TBAI were added directly to the reaction solution and the reaction was continued at 120 °C for 12 hours. In this way, the product **4a** could be isolated in 82% yield.

**Scheme 5:**
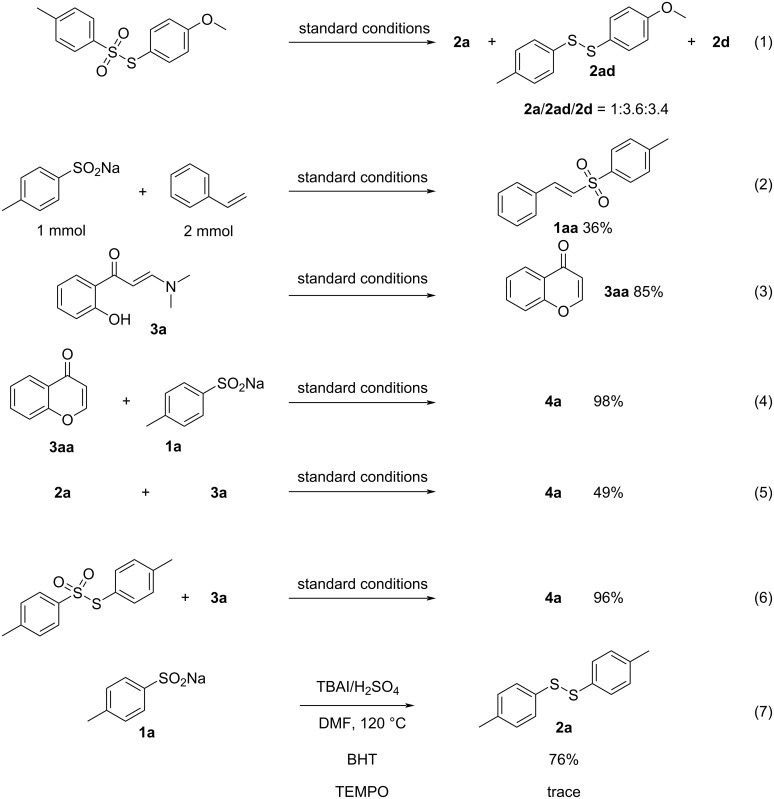
Control experiments.

Several control experiments were performed to investigate the possible mechanism of the reaction (Scheme 5). In Table 1, entry 17, no disulfide product was formed at 80 °C, however, an 85% yield of thiosulfonate was observed. This indicated that thiosulfonate could be an intermediate in the reaction. When the asymmetric thiosulfonate was used as the substrate, unexpectedly a mixture of three disulfide ethers rather than a single disulfide was obtained under the standard reaction conditions (Scheme 5, reaction 1), which led us to conclude that the reaction might be a process in which the thiosulfonate was cleaved and then dimerized to form the disulfide. The reaction of sodium *p*-toluenesulfinate and styrene resulted in the generation of the corresponding vinyl sulfone (Scheme 5, reaction 2), suggesting the formation of *p*-toluenesulfonyl iodide during the reaction [50–51]. Control experiments were performed for the 3-thioflavone formation process. It was found that without the addition of sodium *p*-toluenesulfinate, **3a** was converted to 4*H*-chromen-4-one (**3aa**) in high yield (reaction 3 in Scheme 5). When **3aa** and **1a** were used as substrates, product **4a** could be obtained in high yield (reaction 4, Scheme 5). Additionally, the reaction of **3a** with disulfide and thiosulfonate yielded product **4a** in 49% (reaction 5 in Scheme 5) and 96% (reaction 6), respectively, confirming that the reaction proceeded mainly by the conversion of thiosulfonate to 3-thioflavone. In order to verify whether the reaction proceeded through a free radical mechanism, different free radical inhibitors were added to the reaction (reaction 7, Scheme 5). The addition of TEMPO ((2,2,6,6-tetramethylpiperidin-1-yl)oxyl) resulted in complete inhibition of the reaction, while BHT (2,6-di-*tert*-butyl-4-methylphenol) exhibited a lesser effect. Considering that TEMPO, as an oxidizing agent, affected the reaction, it could be concluded that the reaction did not proceed through a free radical mechanism.

**Scheme 6:**
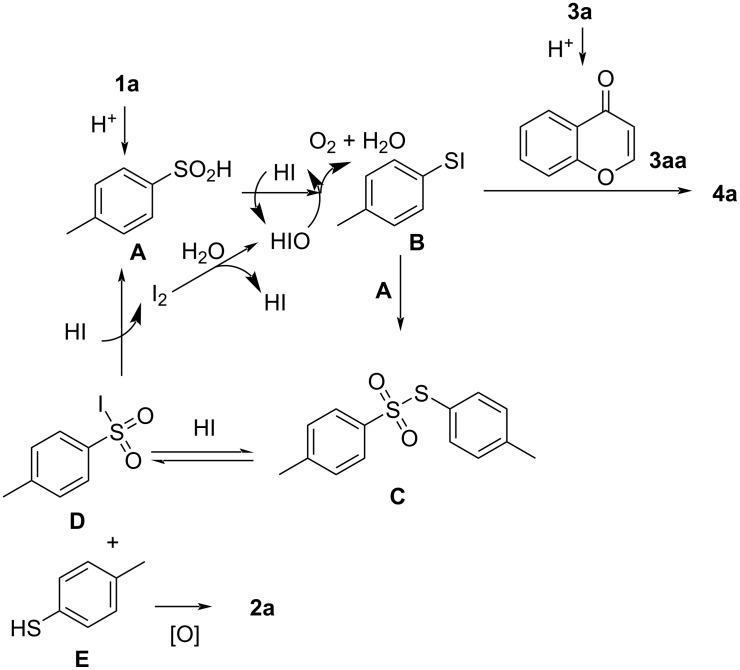
Plausible reaction mechanism.

Based on the results of the control experiments and the related literature [26,30,41,52], a possible mechanism for the model reaction was proposed (Scheme 6). First, **1a** was acidified to form *p*-methylbenzenesulfinic acid **A**, and **A** was reduced by HI to form *p*-tolyl hypoiodothioite **B**, which then reacted with **A** to form the thiosulfonate **C**. **3a** undergoes a cyclization reaction to form **3aa** in an acidic environment. Compound **B** reacted with **3aa** to form **4a** and HI. However, under high-temperature conditions, **C** also reacted with HI to yield **D** and **E**. The latter was then oxidized to form **2a**, while **D** was regenerated to **A** by reaction with HI. Hypoiodous acid underwent a reaction to complete the cycle of iodide ions by decomposing to produce oxygen and HI.

## Conclusion

In summary, we have developed a new TBAI-catalyzed method for the synthesis of symmetrical disulfides using sodium sulfinates as starting materials. The method requires no additional redox reagents and is broadly applicable to both sodium arylsulfinates and sodium alkylsulfinates. This feature will make it a reaction of choice for the pharmaceutical and chemical industries. In addition, a wide variety of 3-sulfenylchromones could be generated by the use of enaminones as the chromone precursors.

## Supporting Information

File 1 Experimental procedures, compound characterization data, and copies of NMR spectra.

## Data Availability

All data that supports the findings of this study is available in the published article and/or the supporting information to this article.
