# Supplementary material for: Synthesis of disulfides and 3-sulfenylchromones from sodium sulfinates catalyzed by TBAI
Source: Beilstein J Org Chem. 2025 Feb 3;21:253–61. doi: 10.3762/bjoc.21.17 (PMC11809575; doi:10.3762/bjoc.21.17)

BEILSTEIN JOURNAL OF ORGANIC CHEMISTRY

Supporting Information

for

Synthesis of disulfides and 3-sulfenylchromones from sodium sulfinates catalyzed by TBAI

Zhenlei Zhang, Ying Wang, Xingxing Pan, Manqi Zhang, Wei Zhao, Meng Li and Hao Zhang

Beilstein J. Org. Chem. **2025,** *21,* 253–261. doi:10.3762/bjoc.21.17

Experimental procedures, compound characterization data, and copies of NMR spectra

License and Terms: This is a supporting information file under the terms of the Creative Commons Attribution License (https://creativecommons.org/licenses/by/4.0). Please note that the reuse, redistribution and reproduction in particular requires that the author(s) and source are credited and that individual graphics may be subject to special legal provisions.
The license is subject to the *Beilstein Journal of Organic Chemistry* terms and conditions: (https://www.beilstein-journals.org/bjoc/terms)

Experimental section

General information: Commercially available reagents were used as received. Solvents were commercially available and used without further purification. Column chromatographic purifications of the compounds were performed using silica gel (mesh 200–300) and petroleum ether/ethyl acetate mixtures as eluent unless otherwise specified. NMR spectra were recorded on a 400 MHz instrument at 25 °C. The chemical shift values are reported in parts per million (ppm) with respect to residual trichloromethane (7.26 ppm for ^{1}H and 77.16 ppm for ^{13}C). The peak patterns are designated as follows: s: singlet; d: doublet; t: triplet; q: quartet; m: multiplet; dd: doublet of doublets; td: triplet of doublets; br s: broad singlet. The coupling constants (*J*) are reported in hertz (Hz).

Typical procedure for the synthesis of sodium thiophene-2-sulfinate: Sodium thiophene-2-sulfinate was prepared by heating 2.08 g of sodium hydrogen sulfite, 1.82 g of thiophene-2-sulfonyl chloride, and 1.68 g of sodium bicarbonate in 20 mL of water at 100 °C for 12 h. After cooling to room temperature, water was removed under vacuum. Recrystallization of the residue in ethanol produced a white solid; yield: 1.02 g (60%).

Similarly, other sodium arylsulfinates were prepared from their corresponding sulfonyl chlorides.

General procedure for the synthesis of compounds 2a–o: Sodium sulfinates **1** (1 mmol), TBAI (0.2 mmol) and H_2SO_4 (1 mmol) were added to DMF (0.5 mL) and the mixture was stirred at 120 °C under air for 2 h. After the reaction was finished, the reaction mixture was diluted with H_2O (15 mL) and extracted with ethyl acetate (3 × 15 mL). The organic extracts were dried over anhydrous Na_2SO_4. After filtration and evaporation of the solvents under reduced pressure, the crude product was purified by column chromatography on silica gel to afford desired product.

General procedure for the synthesis of compounds 4a–p: Sodium sulfinates **1** (1 mmol), enaminones (0.5 mmol), TBAI (0.2 mmol) and H_2SO_4 (1 mmol) were added to DMF (1 mL) and the mixture was stirred at 120 °C under air for 12 h. After the reaction was finished, the reaction mixture was diluted with H_2O (15 mL) and extracted with ethyl acetate (3 × 15 mL). The organic extracts were dried over anhydrous Na_2SO_4. After filtration and evaporation of the solvents under reduced pressure, the crude product was purified by column chromatography on silica gel to afford desired product.

Gram-scale experimental procedure for compound 2a: Sodium 4-methylbenzenesulfinate (**1a**, 20 mmol), TBAI (4 mmol) and H_2SO_4 (20 mmol) were added to DMF (10 mL) and the mixture was stirred at 120 °C under air for 12 h. After the reaction was finished, the reaction mixture was diluted with H_2O (50 mL) and extracted with ethyl acetate (3 × 50 mL). The organic extracts were dried over anhydrous Na_2SO_4. After filtration and evaporation of the solvents under reduced pressure, the crude product was purified by column chromatography on silica gel to afford desired product.

Gram-scale experimental procedure for compound 4a: Sodium 4-methylbenzenesulfinate (**1a**, 20 mmol), (*E*)-3-(dimethylamino)-1-(2-hydroxyphenyl)prop-2-en-1-one (**3a**, 10 mmol), TBAI (4 mmol) and H_2SO_4 (20 mmol) were added to DMF (20 mL) and the mixture was stirred at 120 °C under air for 12 h. After the reaction was finished, the reaction mixture was diluted with H_2O (50 mL) and extracted with ethyl acetate (3 × 50 mL). The organic extracts were dried over anhydrous Na_2SO_4. After filtration and evaporation of the solvents under reduced pressure, the crude product was purified by column chromatography on silica gel to afford desired product.

One-pot synthesis for 4a: 1-(2-Hydroxyphenyl)ethan-1-one (1 mmol) and DMF-DMA (1.2 mmol) was stirred at 120 °C under air for 2 h. The reaction mixture was cooled to room temperature, then **1a** (2 mmol), TBAI (0.4 mmol), H_2SO_4 (2 mmol) and DMF (2 mL) were added to the reaction mixture. The mixture was stirred at 120 °C under air for 2 h. After the reaction was finished, the reaction mixture was diluted with H_2O (15 mL) and extracted with ethyl acetate (3 × 15 mL). The organic extracts were dried over anhydrous Na_2SO_4. After filtration and evaporation of the solvents under reduced pressure, the crude product was purified by column chromatography on silica gel to afford desired product.

Characterization of synthesized compounds:

1,2-Di-*p*-tolyldisulfane (**2a**)[1]

The title compound was prepared according to the general working procedure and purified by column chromatography (petroleum ether / ethyl acetate = 30:1) to give the product **2a** (98 mg; yellow solid; yield 80%):^{1}H NMR (400 MHz, Chloroform-*d*) δ = 7.52 – 7.35 (m, 4H), 7.13 (d, *J*=8.0, 4H), 2.34 (s, 6H). ^{13}C NMR (101 MHz, Chloroform-*d*) δ = 137.53, 133.96, 129.90, 128.59, 21.19.

1,2-Diphenyldisulfane (**2b**)[1]

The title compound was prepared according to the general working procedure and purified by column chromatography (petroleum ether / ethyl acetate = 30:1) to give the product **2b** (88 mg; yellow solid; yield 81%):^{1}H NMR (400 MHz, Chloroform-*d*) δ = 7.57 (td, *J*=7.7, 7.7, 4.0, 4H), 7.36 (dq, *J*=7.6, 4.7, 4.7, 4.5, 4H), 7.28 (dd, *J*=7.0, 2.2, 2H). ^{13}C NMR (101 MHz, Chloroform-*d*) δ = 137.16, 129.22, 127.63, 127.30.

1,2-Dimesityldisulfane (**2c**)[2]

The title compound was prepared according to the general working procedure and purified by column chromatography (petroleum ether / ethyl acetate = 30:1) to give the product **2c** (90 mg; white solid; yield 60%):^{1}H NMR (400 MHz, Chloroform-*d*) δ = 6.84 (s, 4H), 2.26 (s, 6H), 2.21 (s, 12H). ^{13}C NMR (101 MHz, Chloroform-*d*) δ = 143.35, 139.38, 131.65, 128.99, 21.56, 21.27.

1,2-Bis(4-methoxyphenyl)disulfane (**2d**)[2]

The title compound was prepared according to the general working procedure and purified by column chromatography (petroleum ether / ethyl acetate = 30:1) to give the product **2d** (124 mg; white solid; yield 89%): ^{1}H NMR (400 MHz, Chloroform-*d*) δ = 6.84 (s, 4H), 2.26 (s, 6H), 2.21 (s, 12H). ^{13}C NMR (101 MHz, Chloroform-*d*) δ = 143.35, 139.38, 131.65, 128.99, 21.56, 21.27.

1,2-Bis(4-(*tert*-butyl)phenyl)disulfane (**2e**)[2]

The title compound was prepared according to the general working procedure and purified by column chromatography (petroleum ether / ethyl acetate = 30:1) to give the product **2e** (120 mg; white solid; yield 73%): ^{1}H NMR (400 MHz, Chloroform-*d*) δ = 7.53 – 7.41 (m, 4H), 7.39 – 7.31 (m, 4H), 1.31 (s, 18H). ^{13}C NMR (101 MHz, Chloroform-*d*) δ = 150.51, 134.02, 127.72, 126.17, 34.59, 31.31.

1,2-Di(naphthalen-2-yl)disulfane (**2f**)[2]

The title compound was prepared according to the general working procedure and purified by column chromatography (petroleum ether / ethyl acetate = 30:1) to give the product **2f** (119 mg; white solid; yield 75%)^{1}H NMR (400 MHz, Chloroform-*d*) δ = 8.00 (d, *J*=1.8, 2H), 7.80 (dd, *J*=9.0, 2.9, 4H), 7.78 – 7.70 (m, 2H), 7.64 (dd, *J*=8.7, 2.0, 2H), 7.47 (td, *J*=6.7, 6.0, 3.6, 4H). ^{13}C NMR (101 MHz, Chloroform-*d*) δ = 134.35, 133.57, 132.59, 129.11, 127.89, 127.59, 126.87, 126.60, 126.36, 125.74.

1,2-Bis(4-bromophenyl)disulfane (**2g**)[2]

The title compound was prepared according to the general working procedure and purified by column chromatography (petroleum ether / ethyl acetate = 30:1) to give the product **2g** (116 mg; yellow solid; yield 62%) ^{1}H NMR (400 MHz, Chloroform-*d*) δ = 7.42 (d, *J*=8.6, 4H), 7.33 (d, *J*=8.6, 4H). ^{13}C NMR (101 MHz, Chloroform-*d*) δ = 135.80, 132.32, 129.44, 121.63.

1,2-Bis(4-chlorophenyl)disulfane (**2h**)[2]

The title compound was prepared according to the general working procedure and purified by column chromatography (petroleum ether / ethyl acetate = 30:1) to give the product **2h** (104 mg; yellow solid; yield 73%) ^{1}H NMR (400 MHz, Chloroform-*d*) δ = 7.40 (m, 4H), 7.28 (m, 4H). ^{13}C NMR (101 MHz, Chloroform-*d*) δ = 135.16, 133.66, 129.34, 129.34.

1,2-Bis(4-fluorophenyl)disulfane (**2i**)[2]

The title compound was prepared according to the general working procedure and purified by column chromatography (petroleum ether / ethyl acetate = 30:1) to give the product **2i** (90 mg; yellow liquid; yield 71%) ^{1}H NMR (400 MHz, Chloroform-*d*) δ = 7.51 – 7.40 (m, 4H), 7.08 – 6.95 (m, 4H). ^{13}C NMR (101 MHz, Chloroform-*d*) δ = 162.70 (d, *J*=248.46 Hz), 132.26 (d, *J*=3.03 Hz), 131.36 (d, *J*=9.09 Hz), 116.41 (d, *J*=22.22 Hz).

1,2-Di(thiophen-2-yl)disulfane (**2j**)[2]

The title compound was prepared according to the general working procedure and purified by column chromatography (petroleum ether / ethyl acetate = 30:1) to give the product **2j** (89 mg; yellow liquid; yield 77%) H NMR (400 MHz, Chloroform-*d*) δ = 7.50 (dd, *J*=5.3, 1.3, 2H), 7.16 (dd, *J*=3.6, 1.3, 2H), 7.02 (dd, *J*=5.3, 3.6, 2H). ^{13}C NMR (101 MHz, Chloroform-*d*) δ = 135.83, 135.79, 132.42, 127.89.

1,2-Bis(4-(trifluoromethyl)phenyl)disulfane (**2k**)[2]

The title compound was prepared according to the general working procedure and purified by column chromatography (petroleum ether / ethyl acetate = 30:1) to give the product **2k** (102 mg; yellow solid; yield 58%) ^{1}H NMR (400 MHz, Chloroform-*d*) δ = 7.70 – 7.48 (m, 8H). ^{13}C NMR (101 MHz, Chloroform-*d*) δ = 140.91, 129.94, 128.44, 126.65, 126.30 (q, *J*=4.04 Hz).

1,2-Bis(4-nitrophenyl)disulfane (**2l**)[2]

The title compound was prepared according to the general working procedure and purified by column chromatography (petroleum ether / ethyl acetate = 30:1) to give the product **2l** (80 mg; yellow solid; yield 52%) ^{1}H NMR (400 MHz, Chloroform-*d*) δ = 8.19 (d, *J*=8.9, 4H), 7.61 (d, *J*=9.0, 4H). ^{13}C NMR (101 MHz, Chloroform-*d*) δ = 147.05, 144.18, 126.45, 124.60.

1,2-Dibenzyldisulfane (**2m**)[3]

The title compound was prepared according to the general working procedure and purified by column chromatography (petroleum ether / ethyl acetate = 30:1) to give the product **2m** (60 mg; colorless liquid; yield 49%) ^{1}H NMR (400 MHz, Chloroform-*d*) δ = 7.41 (dd, *J*=7.2, 1.8, 4H), 7.38 – 7.21 (m, 6H), 4.49 (s, 4H).^{13}C NMR (101 MHz, Chloroform-*d*) δ = 139.40, 128.96, 128.86, 128.02, 5.93.

1,2-Dibutyldisulfane (**2n**)[3]

The title compound was prepared according to the general working procedure and purified by column chromatography (petroleum ether / ethyl acetate = 30:1) to give the product **2n** (75 mg; colorless liquid; yield 84%) ^{1}H NMR (400 MHz, Chloroform-*d*) δ = 2.76 – 2.63 (m, 4H), 1.73 – 1.59 (m, 4H), 1.47 – 1.32 (m, 4H), 0.92 (t, *J*=7.4, 6H). ^{13}C NMR (101 MHz, Chloroform-*d*) δ = 38.97, 31.42, 21.79, 13.85.

1,2-Dicyclopropyldisulfane (**2o**)[4]

The title compound was prepared according to the general working procedure and purified by column chromatography (petroleum ether / ethyl acetate = 30:1) to give the product **2o** (44 mg; colorless liquid; yield 60%) ^{1}H NMR (400 MHz, Chloroform-*d*) δ = 2.30 (tt, *J*=7.4, 4.3, 2H), 1.00 – 0.92 (m, 4H), 0.73 (qd, *J*=4.9, 2.3, 4H).^{13}C NMR (101 MHz, Chloroform-*d*) δ = 18.74, 9.89.

1-(4-Methoxyphenyl)-2-(*p*-tolyl)disulfane (**2ad**)[5]

The title compound was prepared according to the general working procedure and purified by Preparative Thin-Layer Chromatography to give the product **2ad** (43 mg; white solid; yield 33%) ^{1}H NMR (400 MHz, Chloroform-*d*) δ = 7.49 – 7.38 (m, 2H), 7.29 – 7.23 (m, 2H), 7.21 (d, *J*=8.1, 2H), 6.87 – 6.79 (m, 2H), 3.82 (s, 3H), 2.41 (s, 3H). ^{13}C NMR (101 MHz, Chloroform-*d*) δ = 162.26, 144.63, 140.27, 138.37, 129.43, 127.63, 118.69, 114.97, 55.52, 21.72.

3-(*p*-Tolylthio)-4*H*-chromen-4-one (**4a**)[6]

The title compound was prepared according to the general working procedure and purified by column chromatography (petroleum ether / ethyl acetate = 5:1) to give the product **4a** (127 mg; white solid; yield 95%) ^{1}H NMR (400 MHz, Chloroform-*d*) δ = 8.24 (dd, *J*=8.0, 1.7, 1H), 8.05 (s, 1H), 7.68 (ddd, *J*=8.6, 7.1, 1.7, 1H), 7.48 – 7.39 (m, 2H), 7.36 – 7.32 (m, 2H), 7.11 (d, *J*=8.0, 2H), 2.31 (s, 3H). ^{13}C NMR (101 MHz, Chloroform-*d*) δ = 175.27, 156.43, 137.73, 134.04, 131.08, 130.19, 129.91, 126.53, 125.76, 123.67, 121.17, 118.25, 21.24.

6-Chloro-3-(*p*-tolylthio)-4*H*-chromen-4-one (**4b**)[7]

The title compound was prepared according to the general working procedure and purified by column chromatography (petroleum ether / ethyl acetate = 5:1) to give the product **4b** (146 mg; white solid; yield 97%) ^{1}H NMR (400 MHz, Chloroform-*d*) δ = 8.18 (d, *J*=2.6, 1H), 7.97 (s, 1H), 7.60 (dd, *J*=9.0, 2.6, 1H), 7.37 (dd, *J*=25.6, 8.4, 3H), 7.12 (d, *J*=7.8, 2H), 2.31 (s, 3H). ^{13}C NMR (101 MHz, Chloroform-*d*) δ = 174.10, 155.95, 154.68, 138.08, 134.22, 131.62, 131.47, 130.27, 129.20, 125.75, 124.40, 121.74, 120.00, 21.24.

6-Bromo-3-(*p*-tolylthio)-4*H*-chromen-4-one (**4c**)[7]

The title compound was prepared according to the general working procedure and purified by column chromatography (petroleum ether / ethyl acetate = 5:1) to give the product **4c** (149 mg; white solid; yield 86%)^{1}H NMR (400 MHz, Chloroform-*d*) δ = 8.34 (d, *J*=2.5, 1H), 7.97 (s, 1H), 7.74 (dd, *J*=8.9, 2.5, 1H), 7.34 (d, *J*=8.3, 3H), 7.12 (d, *J*=7.9, 2H), 2.32 (s, 3H).^{13}C NMR (101 MHz, Chloroform-*d*) δ = 173.97, 155.93, 155.15, 138.12, 136.99, 131.52, 130.29, 129.20, 129.00, 124.79, 121.90, 120.22, 119.12, 21.26.

6-Fluoro-3-(*p*-tolylthio)-4*H*-chromen-4-one (**4d**)[8]

The title compound was prepared according to the general working procedure and purified by column chromatography (petroleum ether / ethyl acetate = 5:1) to give the product **4d** (132 mg; white solid; yield 92%) ^{1}H NMR (400 MHz, Chloroform-*d*) δ = 8.24 (dd, *J*=8.7, 6.2, 1H), 7.95 (s, 1H), 7.35 (m, 2H), 7.13 (m, 4H), 2.31 (s, 3H). ^{13}C NMR (101 MHz, Chloroform-*d*) δ = 174.25,165.73 (d, *J*=257.55 Hz), 157.35(d, *J*=13.13 Hz), 155.86 (d, *J*=1.01 Hz), 138.01, 131.47, 130.24, 129.30, 129.05 (d, *J*=11.11 Hz), 121.92, 120.43, 120.41, 114.56 (d, *J*=23.23 Hz), 104.86 (d, *J*=25.25 Hz), 21.22.

7-Methoxy-3-(*p*-tolylthio)-4*H*-chromen-4-one (**4e**)[8]

The title compound was prepared according to the general working procedure and purified by column chromatography (petroleum ether / ethyl acetate = 5:1) to give the product **4e** (142 mg; white solid; yield 95%)^{1}H NMR (400 MHz, Chloroform-*d*) δ = 8.13 (d, *J*=9.0, 1H), 7.97 (s, 1H), 7.33 (d, *J*=8.1, 2H), 7.10 (d, *J*=7.8, 2H), 6.97 (dd, *J*=9.0, 2.4, 1H), 6.82 (d, *J*=2.4, 1H), 3.90 (s, 3H), 2.30 (s, 3H). ^{13}C NMR (101 MHz, Chloroform-*d*) δ = 174.57, 164.29, 158.20, 156.04, 137.56, 130.87, 130.14, 130.11, 127.89, 120.90, 117.54, 115.06, 100.29, 56.02, 21.23.

6-Nitro-3-(*p*-tolylthio)-4*H*-chromen-4-one (**4f**)[7]

The title compound was prepared according to the general working procedure and purified by column chromatography (petroleum ether / ethyl acetate = 5:1) to give the product **4f** (91 mg; yellow solid; yield 58%)^{1}H NMR (400 MHz, Chloroform-*d*) δ = 9.07 (d, *J*=2.8, 1H), 8.48 (dd, *J*=9.2, 2.8, 1H), 7.88 (s, 1H), 7.60 (d, *J*=9.2, 1H), 7.38 (d, *J*=7.9, 2H), 7.15 (d, *J*=7.8, 2H), 2.33 (s, 3H). ^{13}C NMR (101 MHz, Chloroform-*d*) δ = 173.75, 159.02, 154.65, 144.95, 138.85, 132.46, 130.49, 128.19, 127.77, 123.81, 123.23, 123.12, 120.11, 21.27.

3-(Thiophen-2-ylthio)-4*H*-chromen-4-one (**4g**)[9]

The title compound was prepared according to the general working procedure and purified by column chromatography (petroleum ether / ethyl acetate = 5:1) to give the product **4g** (96 mg; yellow solid; yield 74%) ^{1}H NMR (400 MHz, Chloroform-*d*) δ = 8.24 (m, 1H), 7.88 (s, 1H), 7.66 (ddd, *J*=8.6, 7.2, 1.7, 1H), 7.43 (m, 3H), 7.37 (dd, *J*=3.6, 1.3, 1H), 7.03 (dd, *J*=5.4, 3.6, 1H). ^{13}C NMR (101 MHz, Chloroform-*d*) δ = 174.92, 156.36, 154.65, 135.95, 134.05, 131.19, 130.13, 128.05, 126.37, 125.74, 123.45, 123.25, 118.24.

3-(Phenylthio)-4*H*-chromen-4-one (**4h**)[6]

The title compound was prepared according to the general working procedure and purified by column chromatography (petroleum ether / ethyl acetate = 5:1) to give the product **4h** (110 mg; white solid; yield 87%)^{1}H NMR (400 MHz, Chloroform-*d*) δ = 8.20 (dd, *J*=8.0, 1.7, 1H), 8.11 (s, 1H), 7.64 (ddd, *J*=8.7, 7.1, 1.7, 1H), 7.47 – 7.29 (m, 4H), 7.24 (dd, *J*=8.6, 6.2, 2H), 7.21 – 7.13 (m, 1H). ^{13}C NMR (101 MHz, Chloroform-*d*) δ = 175.13, 157.49, 156.34, 134.08, 134.04, 129.80, 129.23, 127.14, 126.42, 125.79, 123.64, 119.84, 118.22.

3-(Methylthio)-4*H*-chromen-4-one (**4i**)[6]

The title compound was prepared according to the general working procedure and purified by column chromatography (petroleum ether / ethyl acetate = 5:1) to give the product **4i** (60 mg; white solid; yield 63%)^{1}H NMR (400 MHz, Chloroform-*d*) δ = 8.21 (dd, *J*=8.1, 1.7, 1H), 8.02 (s, 1H), 7.65 (ddd, *J*=8.6, 7.2, 1.7, 1H), 7.52 – 7.30 (m, 2H), 2.38 (s, 3H). ^{13}C NMR (101 MHz, Chloroform-*d*) δ = 175.70, 156.30, 153.91, 133.88, 126.12, 125.53, 123.15, 121.89, 118.16, 16.32.

3-((4-(Trifluoromethyl)phenyl)thio)-4*H*-chromen-4-one (**4j**)

The title compound was prepared according to the general working procedure and purified by column chromatography (petroleum ether / ethyl acetate = 5:1) to give the product **4j** (117 mg; white solid; yield 73%) mp: 161-162 ℃, ^{1}H NMR (400 MHz, Chloroform-*d*) δ = 8.39 (s, 1H), 8.27 (dd, *J*=8.0, 1.7, 1H), 7.76 (ddd, *J*=8.7, 7.1, 1.7, 1H), 7.51 (dt, *J*=16.0, 8.2, 8.2, 4H), 7.38 (d, *J*=8.2, 2H). ^{13}C NMR (101 MHz, Chloroform-*d*) δ = 174.93, 159.52, 156.52, 140.21, 134.50, 128.59, 128.26, 127.66, 126.61, 126.25, 125.96 (q, *J*=4.4 Hz), 123.87, 118.40, 117.30. HRMS calc. $C_{16}H_9F_3O_2S$ (M+Na): 345.0168, found: 345.0179.

3-((4-Fluorophenyl)thio)-4*H*-chromen-4-one (**4k**)[10]

The title compound was prepared according to the general working procedure and purified by column chromatography (petroleum ether / ethyl acetate = 5:1) to give the product **4k** (122 mg; yellow liquid; yield 90%)^{1}H NMR (400 MHz, Chloroform-*d*) δ = 8.21 (dd, *J*=8.0, 1.7, 1H), 8.13 (s, 1H), 7.68 (ddd, *J*=8.7, 7.1, 1.7, 1H), 7.50 – 7.32 (m, 4H), 6.98 (t, *J*=8.6, 2H).^{13}C NMR (101 MHz, Chloroform-*d*) δ = 175.15, 163.62, 161.16, 157.07, 156.37, 134.17, 132.88, 132.80, 128.88, 128.84, 126.42, 125.87, 123.68, 120.47, 118.26, 116.53, 116.31.

3-((4-Chlorophenyl)thio)-4*H*-chromen-4-one (**4l**)[6]

The title compound was prepared according to the general working procedure and purified by column chromatography (petroleum ether / ethyl acetate = 5:1) to give the product **4l** (111 mg; white solid; yield 77%)^{1}H NMR (400 MHz, Chloroform-*d*) δ = 8.34 – 8.17 (m, 2H), 7.75 (ddd, *J*=8.6, 7.1, 1.7, 1H), 7.57 – 7.43 (m, 2H), 7.40 – 7.32 (m, 2H), 7.30 – 7.23 (m, 2H).^{13}C NMR (101 MHz, Chloroform-*d*) δ = 175.11, 158.08, 156.47, 134.32, 133.19, 132.90, 130.92, 129.40, 126.57, 126.05, 123.82, 119.27, 118.34.

3-((4-Bromophenyl)thio)-4*H*-chromen-4-one (**4m**)[6]

The title compound was prepared according to the general working procedure and purified by column chromatography (petroleum ether / ethyl acetate = 5:1) to give the product **4m** (156 mg; white solid; yield 94%)^{1}H NMR (400 MHz, Chloroform-*d*) δ = 8.24 (d, *J*=9.6, 2H), 7.71 (ddd, *J*=8.7, 7.1, 1.7, 1H), 7.52 – 7.42 (m, 2H), 7.42 – 7.34 (m, 2H), 7.29 – 7.19 (m, 2H). ^{13}C NMR (101 MHz, Chloroform-*d*) δ = 175.09, 158.23, 156.48, 134.33, 133.66, 132.30, 131.02, 126.58, 126.07, 123.83, 121.10, 119.04, 118.35.

3-((4-Methoxyphenyl)thio)-4*H*-chromen-4-one (**4n**)[8]

The title compound was prepared according to the general working procedure and purified by column chromatography (petroleum ether / ethyl acetate = 5:1) to give the product **4n** (113 mg; white solid; yield 80%)^{1}H NMR (400 MHz, Chloroform-*d*) δ = 8.21 (dd, *J*=8.0, 1.6, 1H), 7.88 (s, 1H), 7.64 (ddd, *J*=8.6, 7.1, 1.7, 1H), 7.57 – 7.31 (m, 4H), 6.93 – 6.68 (m, 2H), 3.77 (s, 3H). ^{13}C NMR (101 MHz, Chloroform-*d*) δ = 175.20, 159.84, 156.27, 154.99, 134.31, 133.90, 126.28, 125.57, 123.38, 123.03, 122.54, 118.16, 115.04, 55.42.

3-(Naphthalen-2-ylthio)-4*H*-chromen-4-one (**4o**)[9]

The title compound was prepared according to the general working procedure and purified by column chromatography (petroleum ether / ethyl acetate = 5:1) to give the product **4o** (100 mg; white solid; yield 66%) ^{1}H NMR (400 MHz, Chloroform-*d*) δ = 8.25 (dd, *J*=8.0, 1.7, 1H), 8.18 (s, 1H), 7.87 (d, *J*=1.8, 1H), 7.81 – 7.67 (m, 4H), 7.46 (dtd, *J*=15.0, 7.4, 4.0, 5H). ^{13}C NMR (101 MHz, Chloroform-*d*) δ = 175.25, 157.45, 156.46, 134.16, 133.81, 132.41, 131.34, 129.03, 128.87, 127.84, 127.50, 126.72, 126.57, 126.29, 125.89, 123.75, 120.10, 118.30.

3-((4-(*tert*-Butyl)phenyl)thio)-4*H*-chromen-4-one (**4p**)[11]

The title compound was prepared according to the general working procedure and purified by column chromatography (petroleum ether / ethyl acetate = 5:1) to give the product **4p** (118 mg; white solid; yield 76%)^{1}H NMR (400 MHz, Chloroform-*d*) δ = 8.25 (dd, *J*=8.0, 1.7, 1H), 8.18 (s, 1H), 7.87 (d, *J*=1.8, 1H), 7.81 – 7.67 (m, 4H), 7.46 (dtd, *J*=15.0, 7.4, 4.0, 5H). ^{13}C NMR (101 MHz, Chloroform-*d*) δ = 175.25, 157.45, 156.46, 134.16, 133.81, 132.41, 131.34, 129.03, 128.87, 127.84, 127.50, 126.72, 126.57, 126.29, 125.89, 123.75, 120.10, 118.30.

(*E*)-1-Methyl-4-(styrylsulfonyl)benzene (**1aa**)[12]

The title compound was prepared according to the general working procedure and purified by column chromatography (petroleum ether / ethyl acetate = 5:1) to give the product **1aa** (92 mg; white solid; yield 36%) ^{1}H NMR (400 MHz, Chloroform-*d*) δ = 7.92 – 7.74 (m, 2H), 7.66 (d, *J*=15.4, 1H), 7.56 – 7.43 (m, 2H), 7.44 – 7.31 (m, 5H), 6.85 (d, *J*=15.4, 1H), 2.43 (s, 3H). ^{13}C NMR (101 MHz, Chloroform-*d*) δ = 144.54, 142.06, 137.79, 132.53, 131.24, 130.10, 129.19, 128.65, 127.83, 127.67, 21.76.

References

[1] S. Sun, J. Li, L. Pan, H. Liu, Y. Guo, Z. Gao, X. Bi, *Organic & Biomolecular Chemistry.* **2022**, *20*, 8885-8892.

[2] Y. Zheng, F.-L. Qing, Y. Huang, X.-H. Xu, *Advanced Synthesis & Catalysis.* **2016**, *358*, 3477-3481.

[3] C. Zhu, D. Wu, H. Liu, C. Meng, T. Tang, *Green Chemistry.* **2022**, *24*, 9033-9039.

[4] E. Block, A. Schwan, D. A. Dixon, *Journal of the American Chemical Society.* **1992**, *114*, 3492-3499.

[5] K. Yang, Y. Luo, Q. Hu, M. Song, J. Liu, Z. Li, B. Li, X. Sun, *The Journal of Organic Chemistry.* **2023**, *88*, 13699-13711.

[6] W. Zhao, P. Xie, Z. Bian, A. Zhou, H. Ge, M. Zhang, Y. Ding, L. Zheng, *The Journal of Organic Chemistry.* **2015**, *80*, 9167-9175.

[7] Y. Ding, W. Wu, W. Zhao, Y. Li, P. Xie, Y. Huang, Y. Liu, A. Zhou, *Organic & Biomolecular Chemistry.* **2016**, *14*, 1428-1431.

[8] C. Balakrishna, R. Gudipati, V. Kandula, S. Yennam, P. Uma Devi, M. Behera, *New Journal of Chemistry.* **2019**, *43*, 2458-2463.

[9] T. Guo, *Synthetic Communications.* **2017**, *47*, 2053-2061.

[10] W. Zhao, P. Xie, Z. Bian, A. Zhou, H. Ge, B. Niu, Y. Ding, *RSC Advances.* **2015**, *5*, 59861-59864.

[11] J. Zhu, B. Xu, J. Yu, Y. Ren, J. Wang, P. Xie, C. U. Pittman, A. Zhou, *Organic & Biomolecular Chemistry.* **2018**, *16*, 5999-6005.

[12] Y. Zhao, Y.-L. Lai, K.-S. Du, D.-Z. Lin, J.-M. Huang, *The Journal of Organic Chemistry.* **2017**, *82*, 9655-9661.

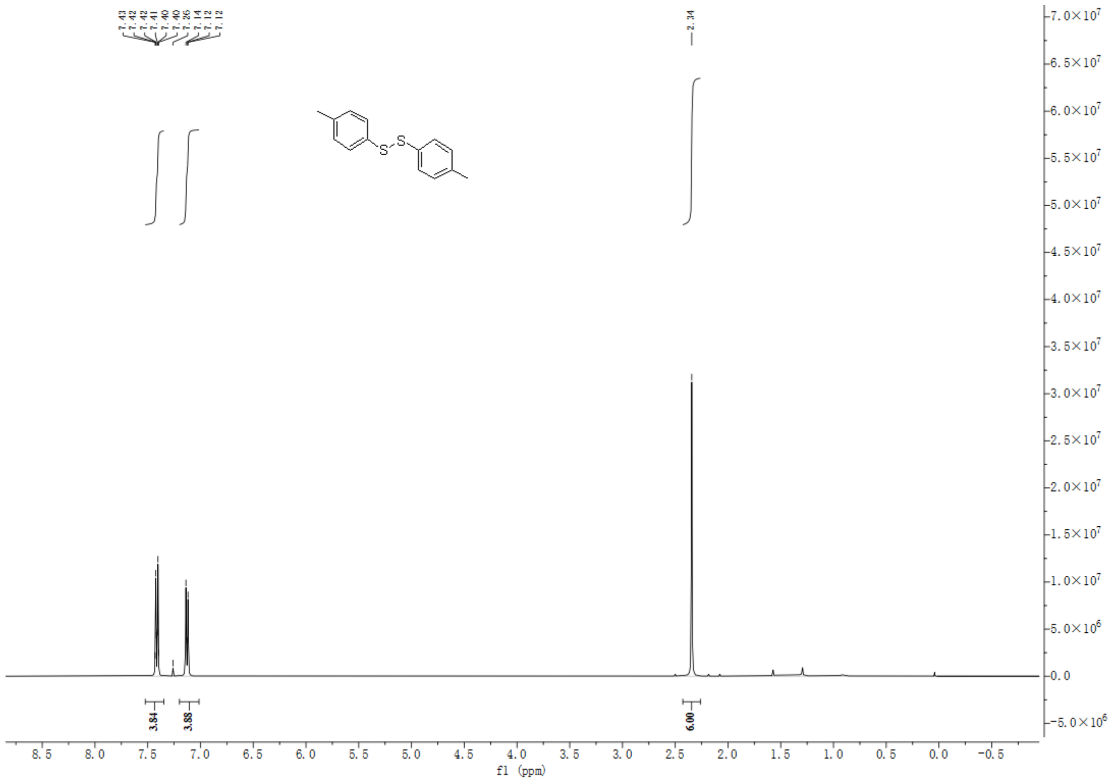
7.43
7.42
7.41
7.40
7.40
7.26
7.14
7.12
7.12
2.34
3.84
3.88
6.00
f1 (ppm)

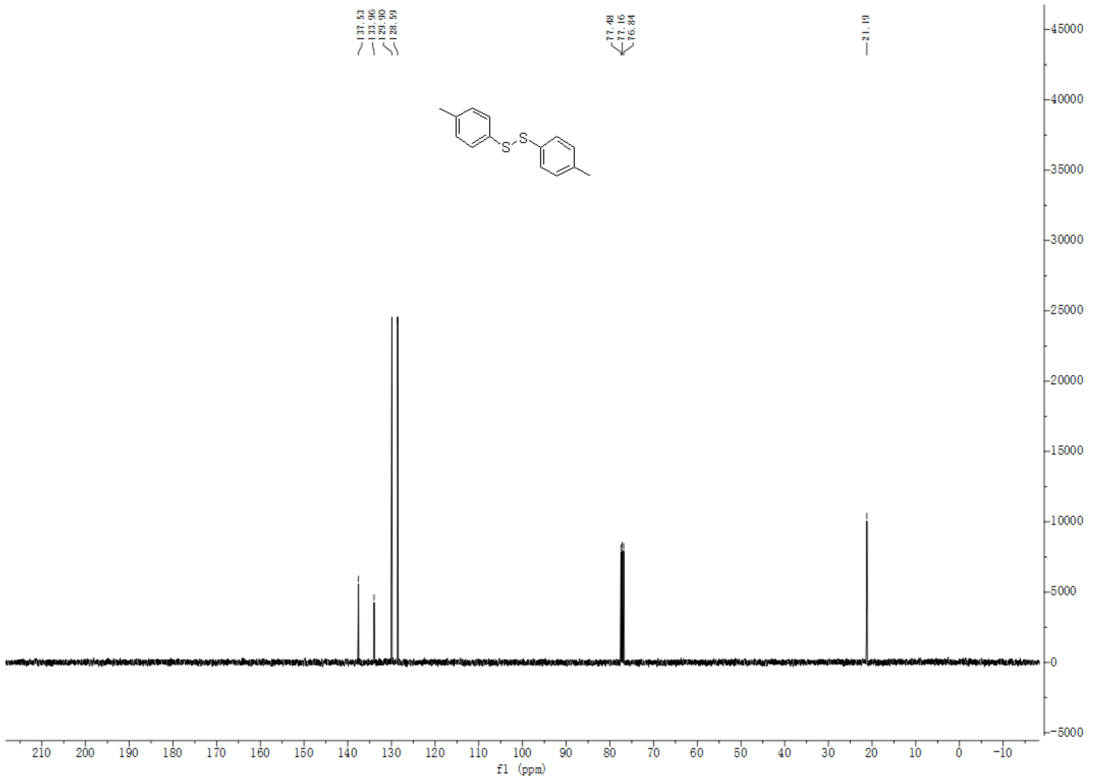
137.53
133.96
129.90
128.59
77.48
77.16
76.84
21.19
f1 (ppm)

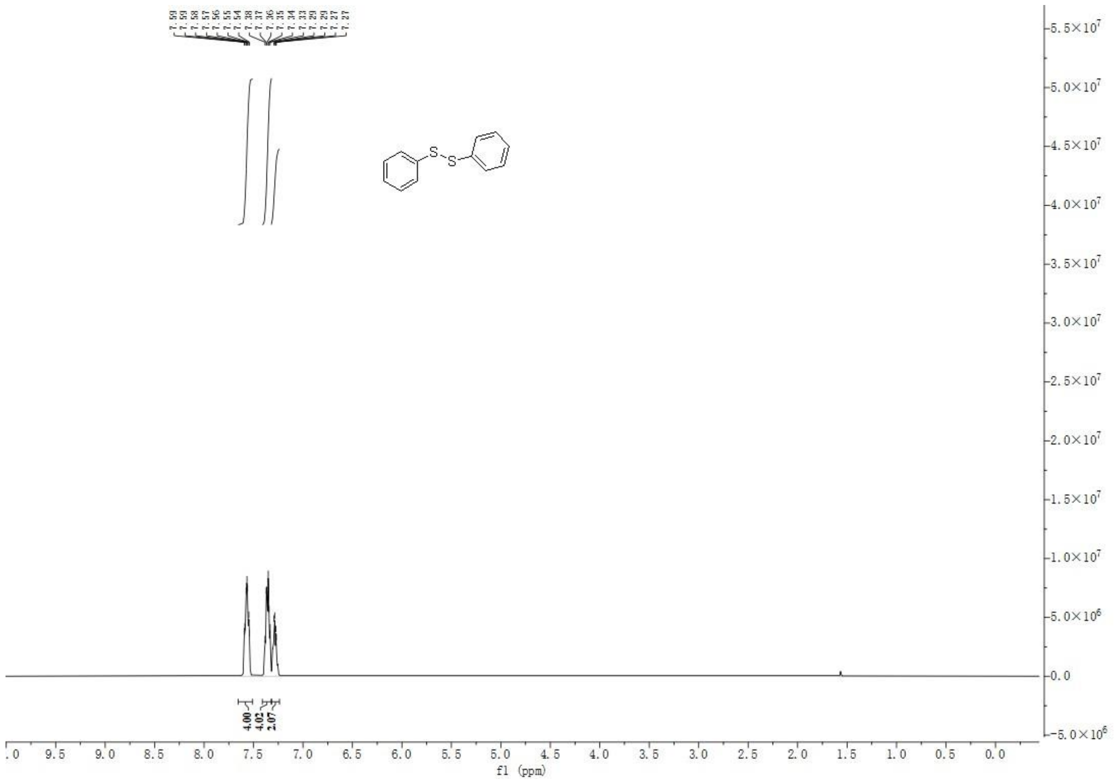

7.59
7.59
7.58
7.57
7.56
7.55
7.54
7.38
7.37
7.36
7.35
7.34
7.33
7.29
7.29
7.27
7.27
4.00
4.02
2.07
f1 (ppm)

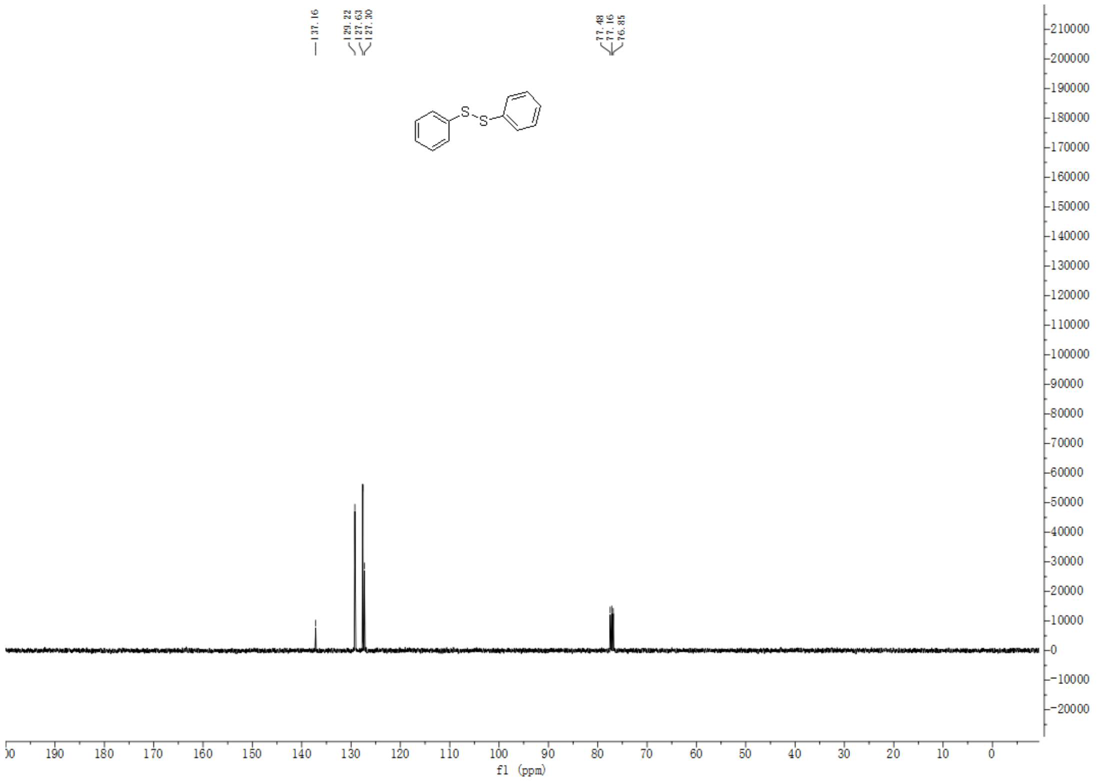

137.16
129.22
127.63
127.30
77.48
77.16
76.85
f1 (ppm)

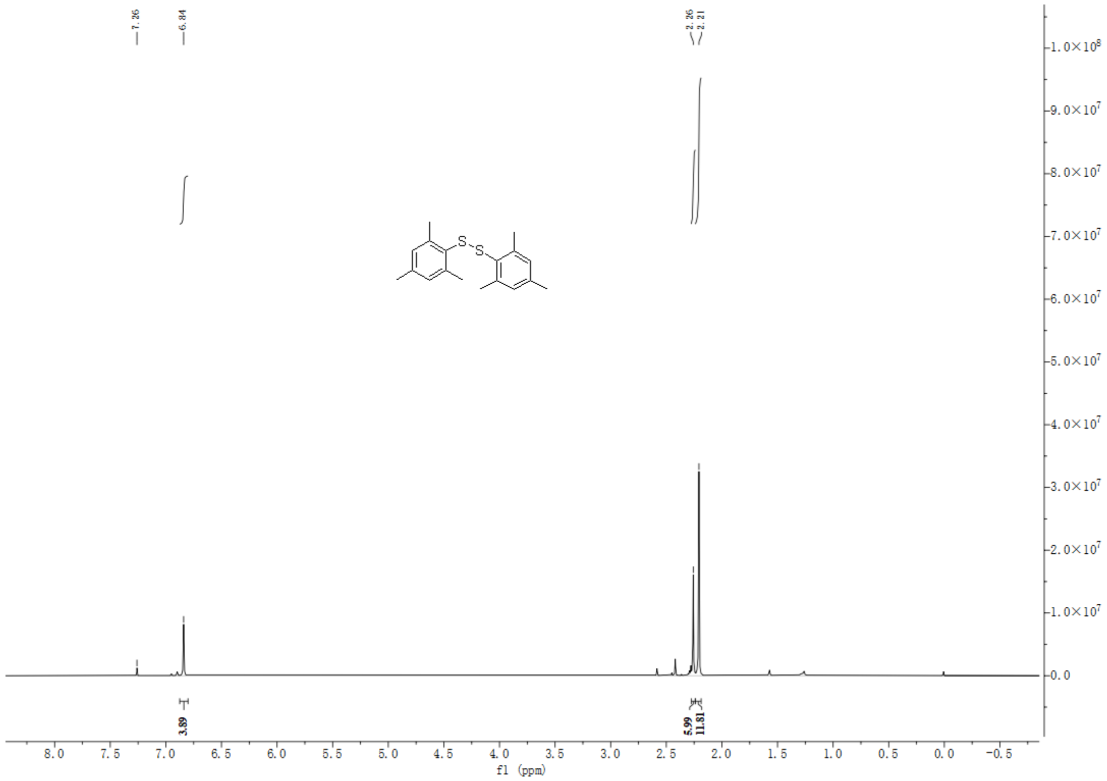
7.26
6.84
2.26
2.21
3.89
5.99
11.81
f1 (ppm)

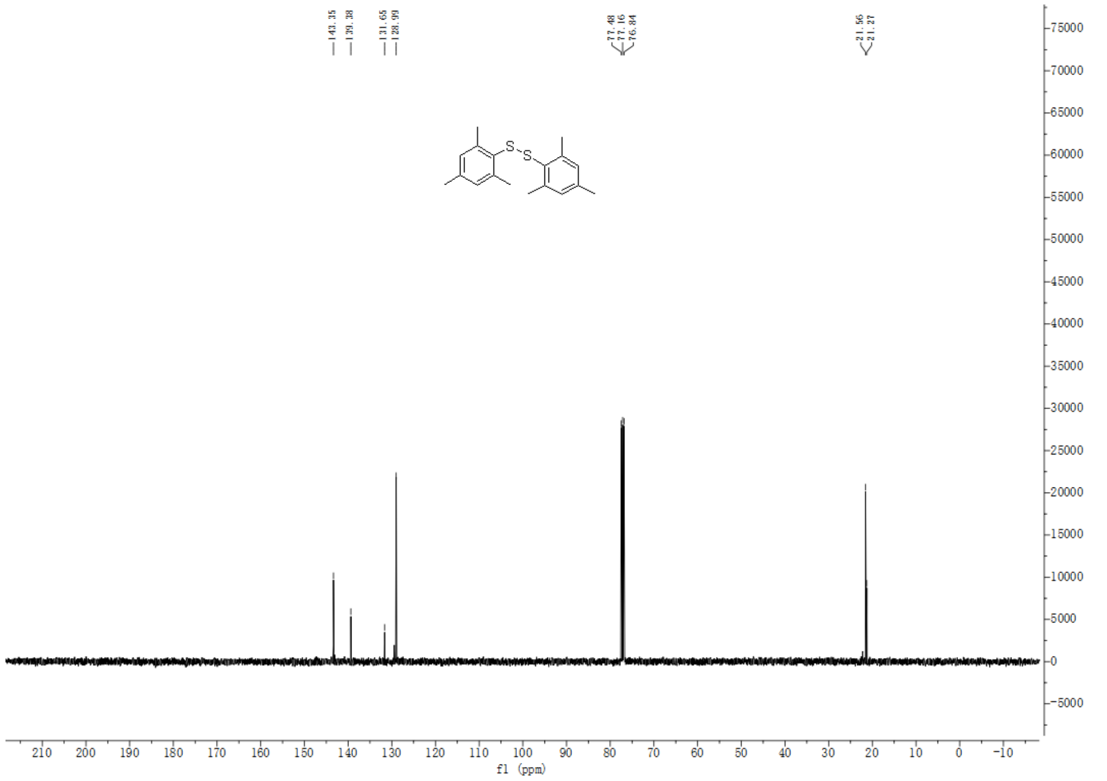
143.35
139.38
131.65
128.99
77.48
77.16
76.84
21.56
21.27
f1 (ppm)

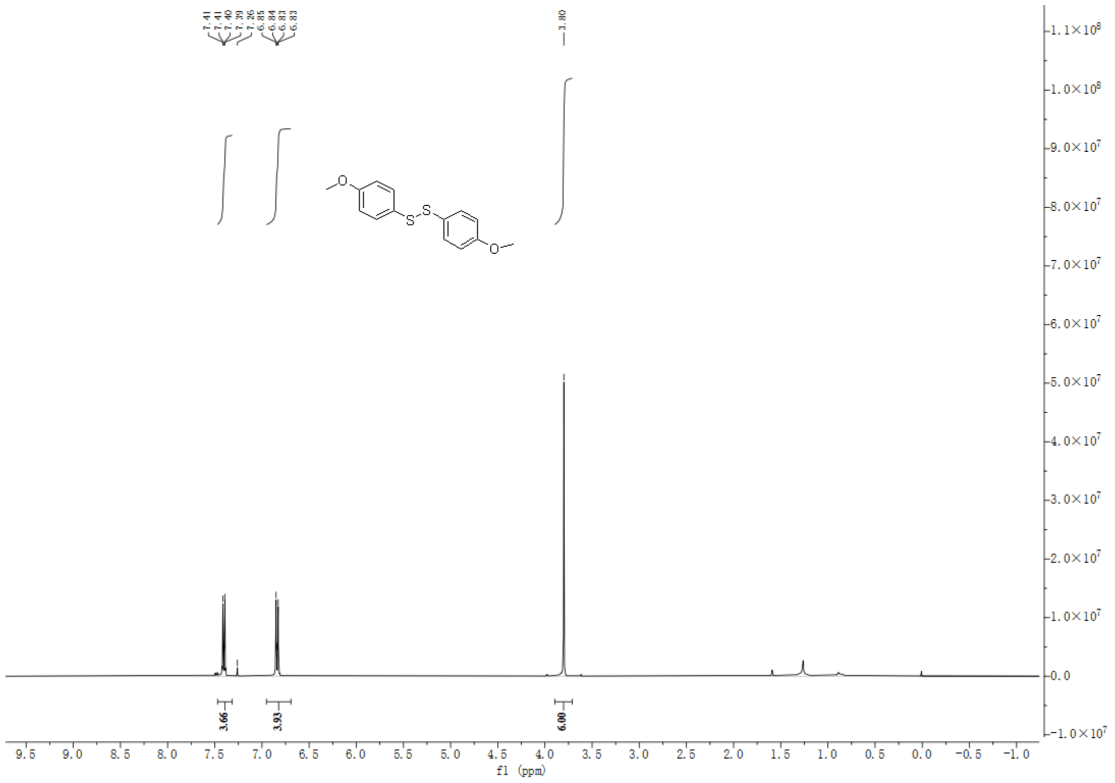

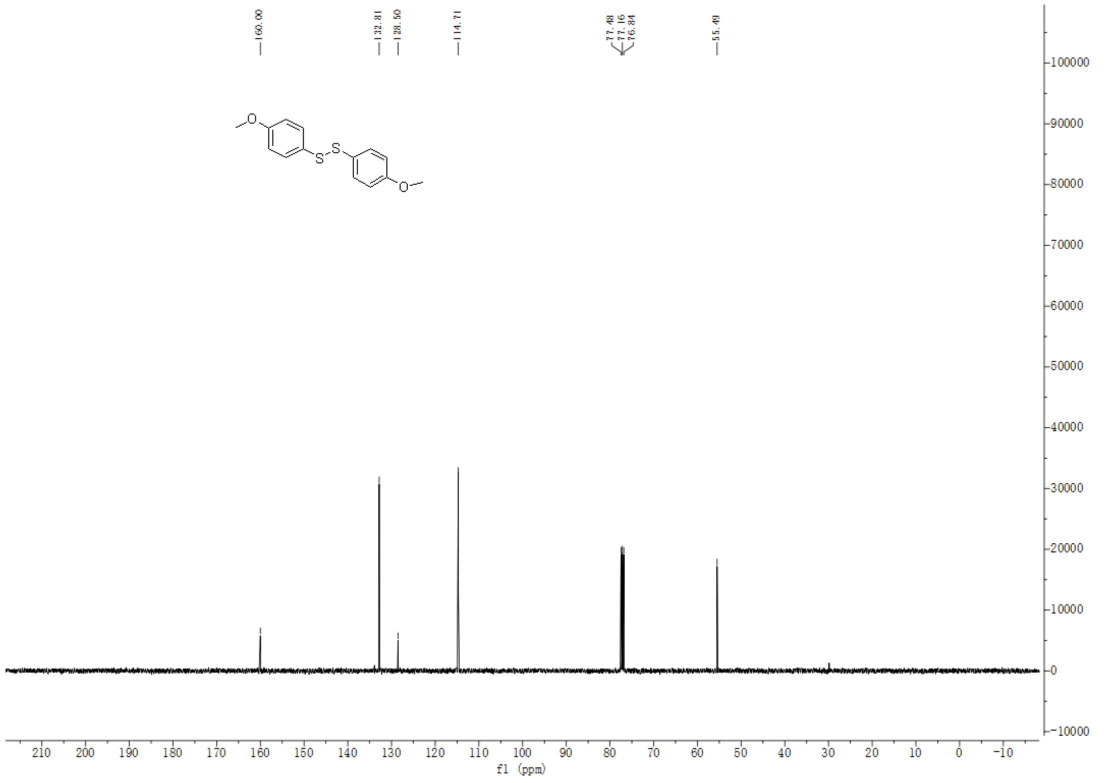

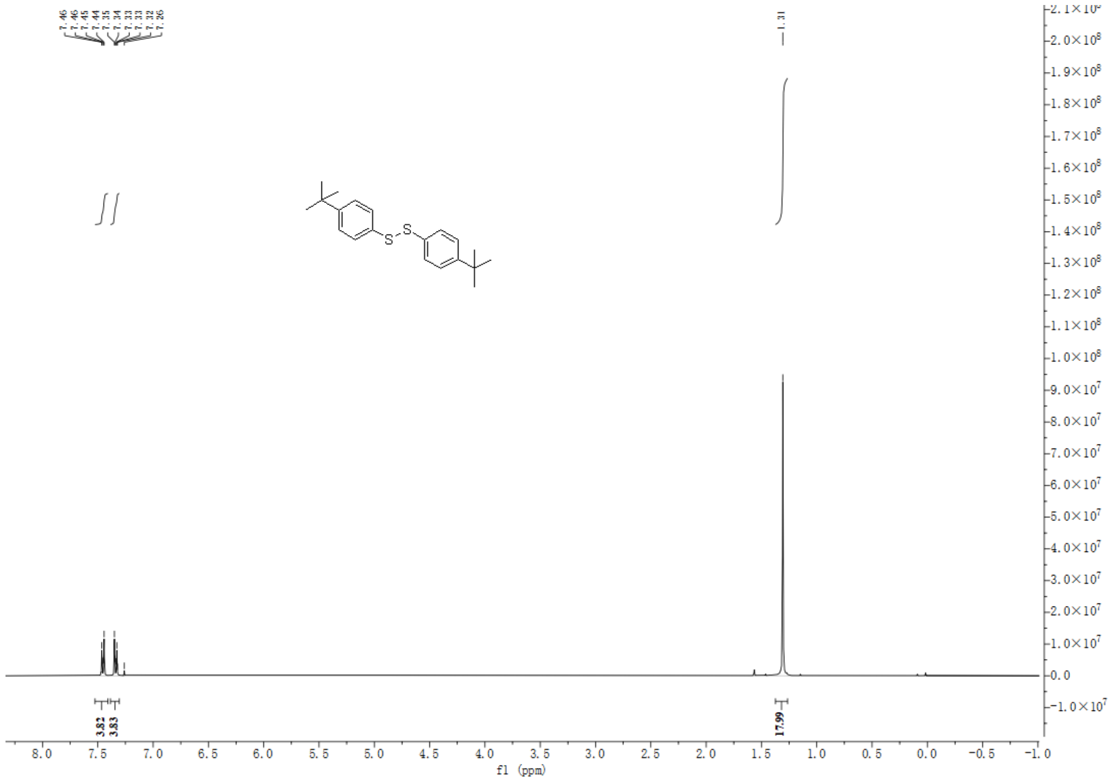
7.46
7.46
7.45
7.44
7.35
7.34
7.33
7.33
7.32
7.26
1.31
3.82
3.83
17.99
S
S
f1 (ppm)

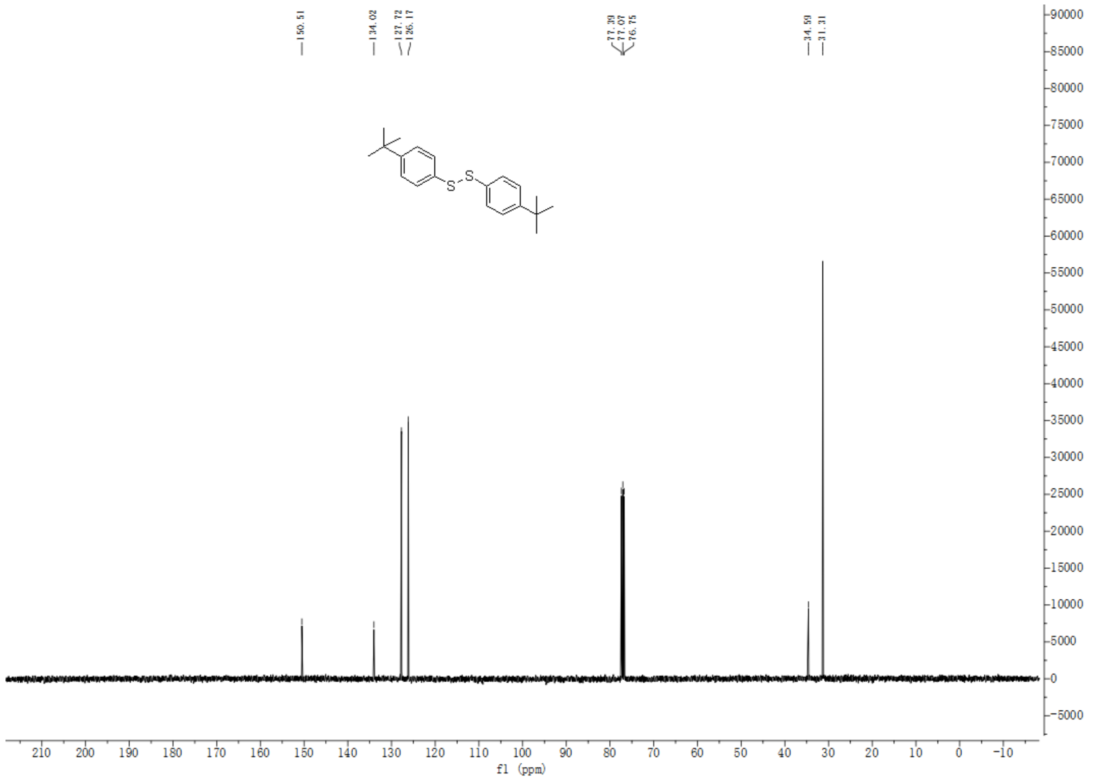
150.51
134.02
127.72
126.17
77.39
77.07
76.75
34.59
31.31
S
S
f1 (ppm)

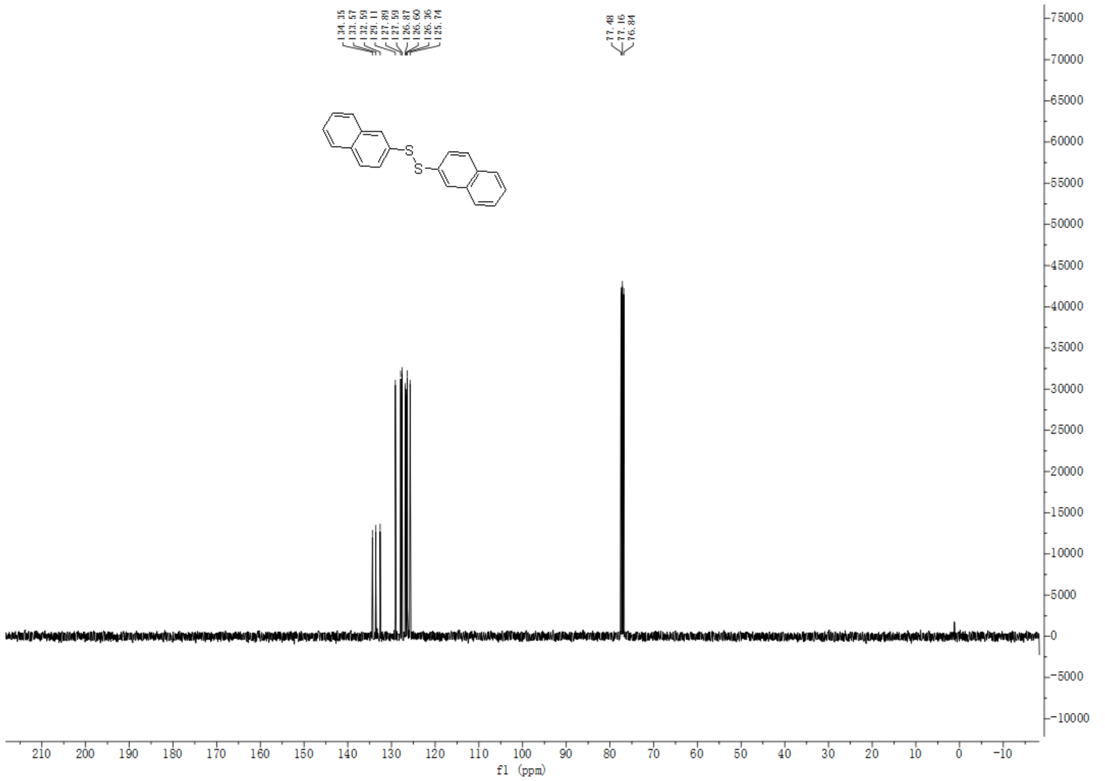
134.35
133.57
132.59
129.11
127.89
127.59
126.87
126.60
126.36
125.74
77.48
77.16
76.84
S
S
f1 (ppm)

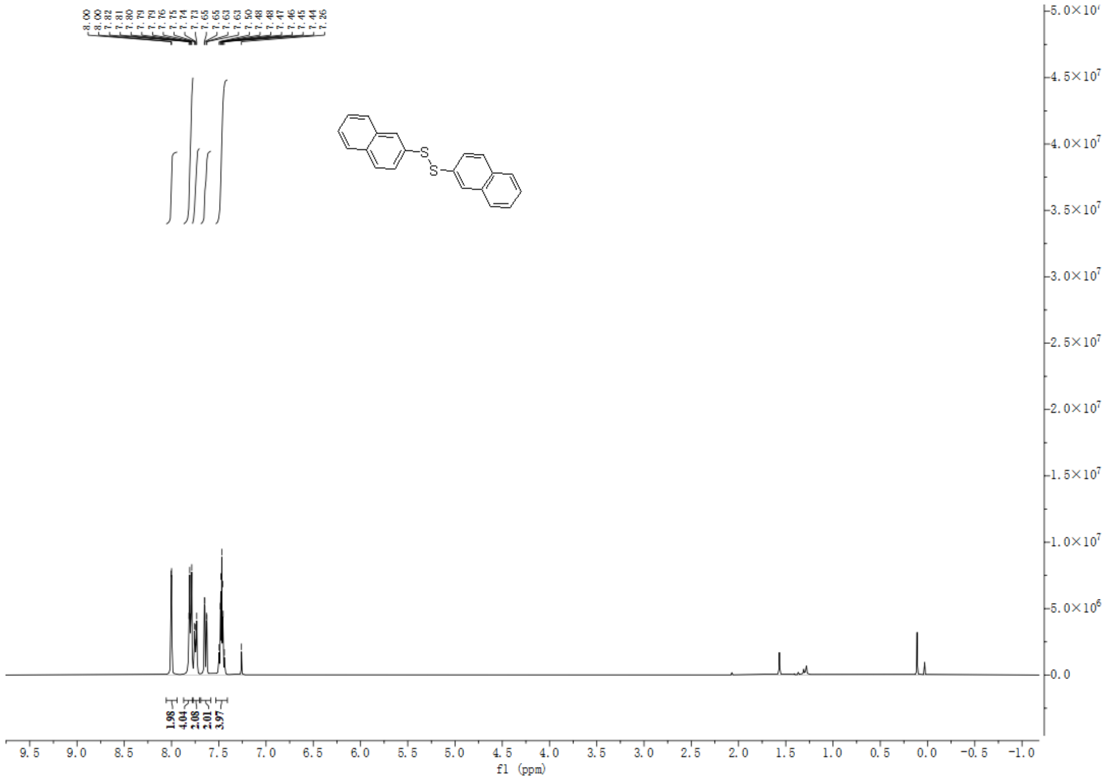
S
S
1.98
4.04
2.08
2.01
3.97
f1 (ppm)

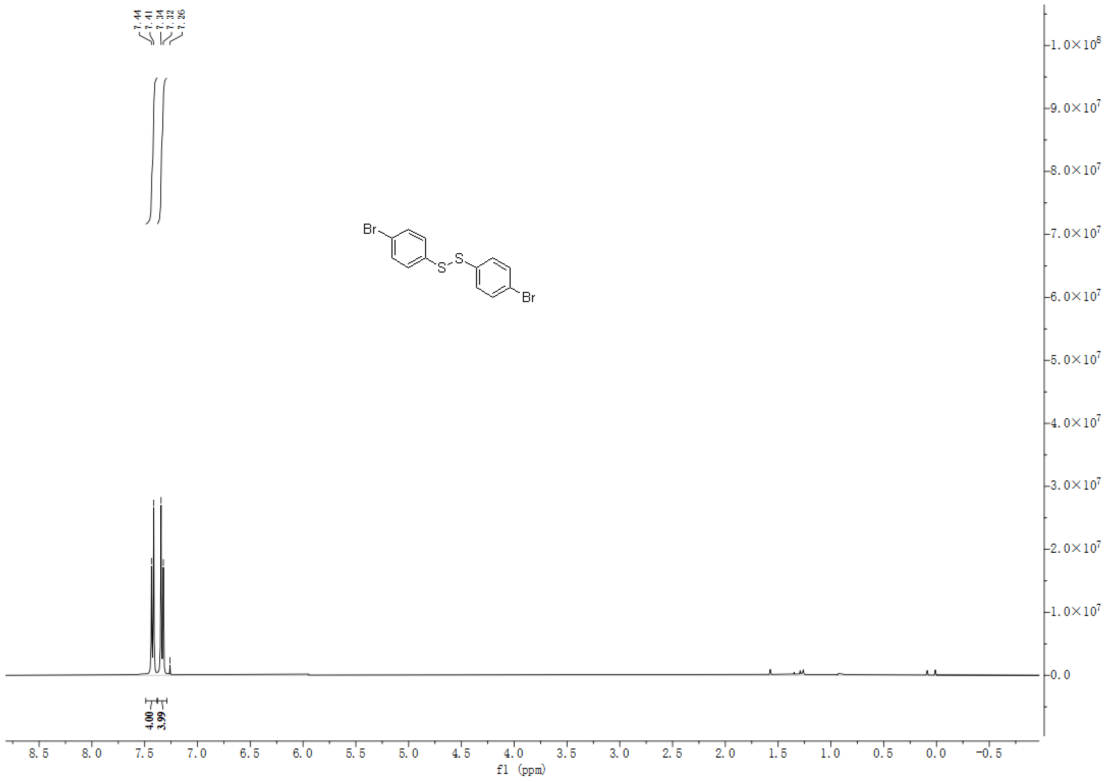
7.44
7.41
7.34
7.32
7.26
Br
S
S
Br
4.00
3.99
f1 (ppm)

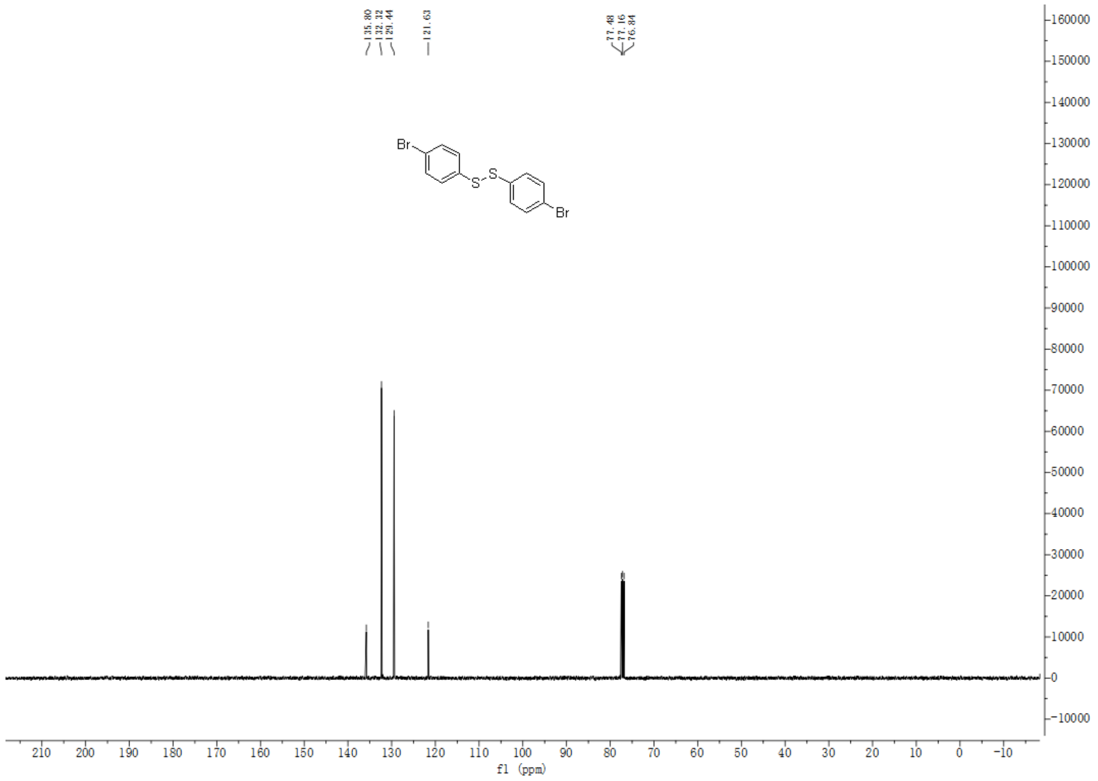
135.80
132.32
129.44
121.63
77.48
77.16
76.84
Br
S
S
Br
f1 (ppm)

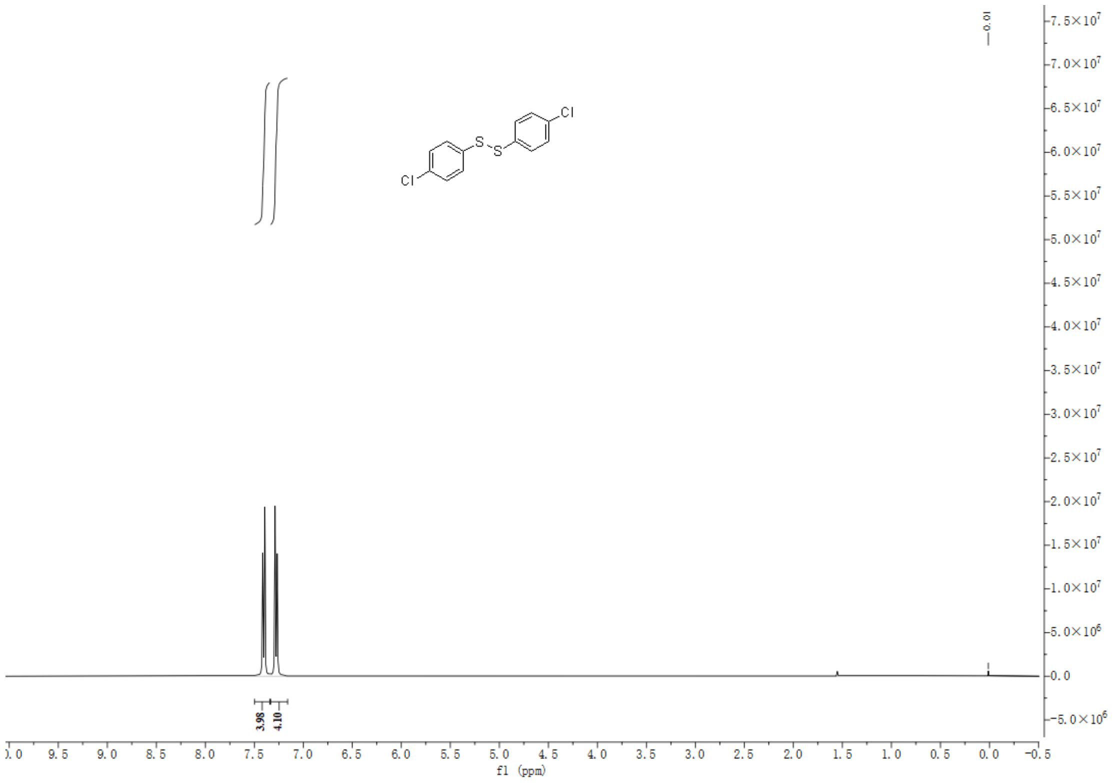
0.01
3.98
4.10
f1 (ppm)

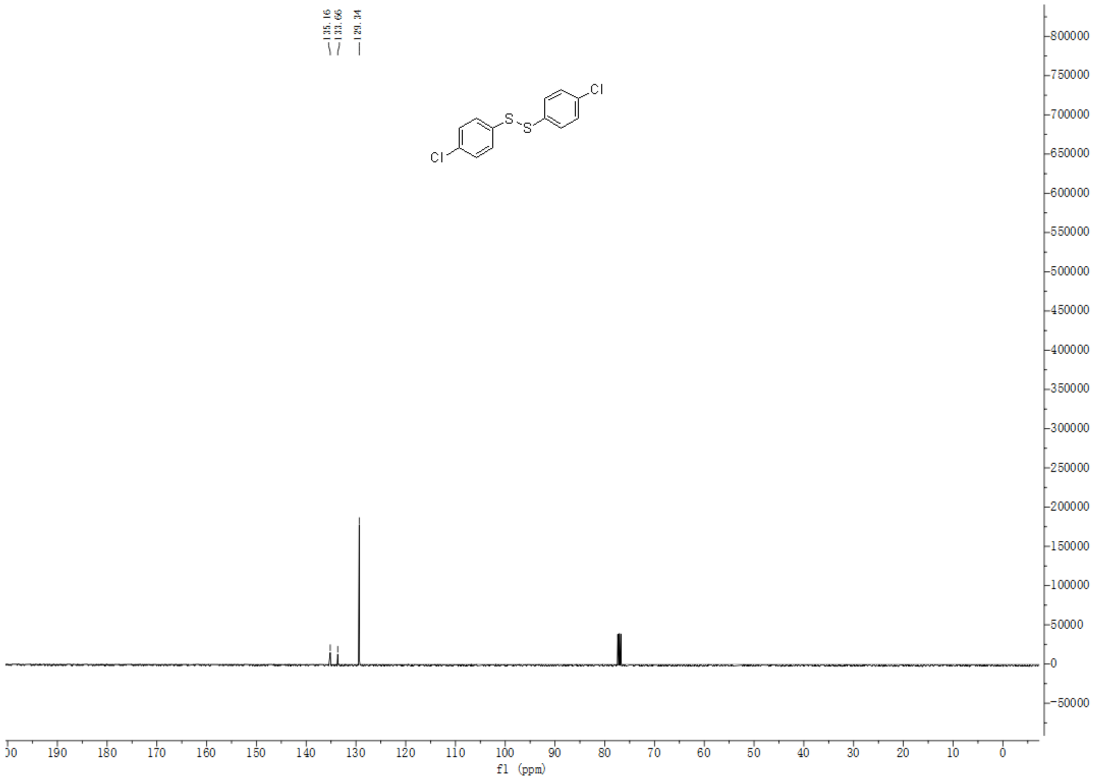
135.16
133.66
129.34
f1 (ppm)

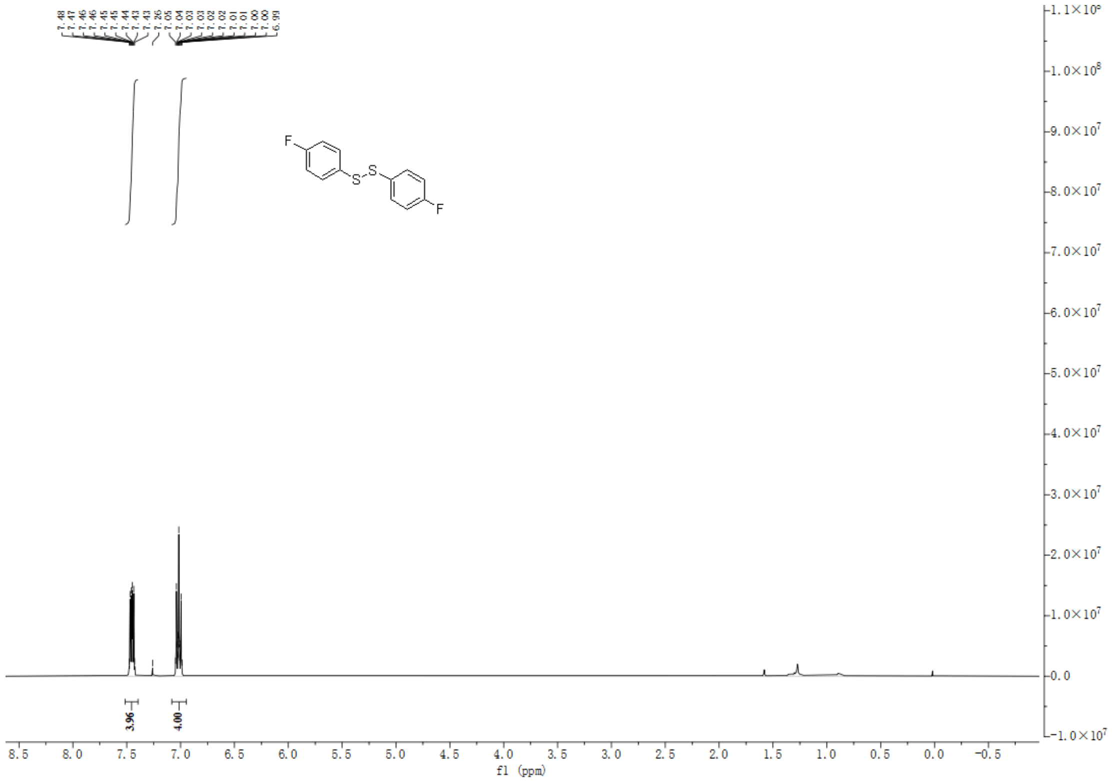
7.48
7.47
7.46
7.46
7.45
7.45
7.44
7.43
7.43
7.26
7.05
7.04
7.03
7.03
7.02
7.02
7.01
7.01
7.00
7.00
6.99
F
S
S
F
3.96
4.00
f1 (ppm)

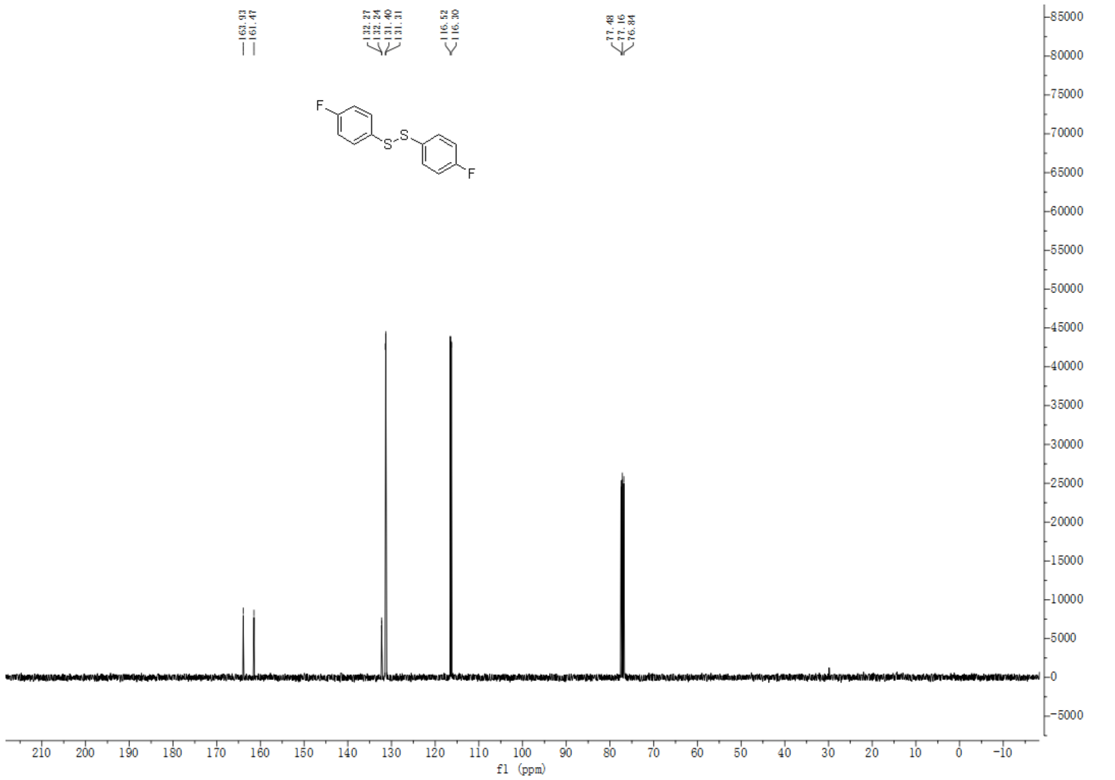
163.93
161.47
132.27
132.24
131.40
131.31
116.52
116.30
77.48
77.16
76.84
F
S
S
F
f1 (ppm)

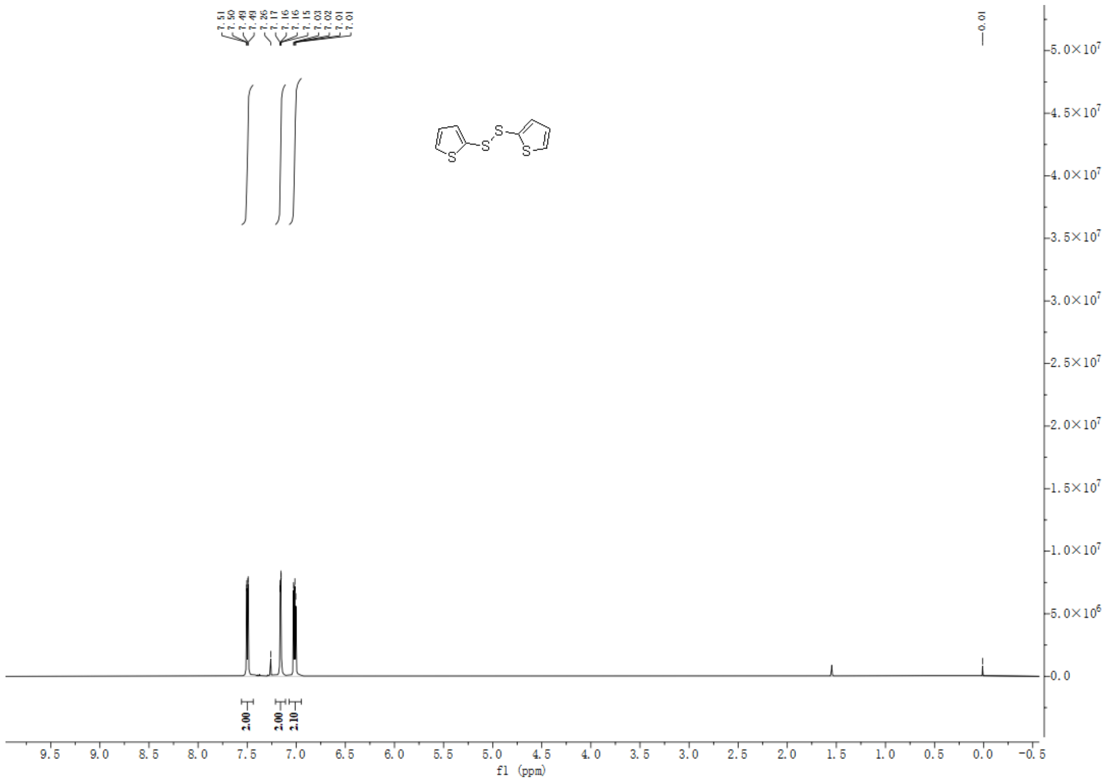

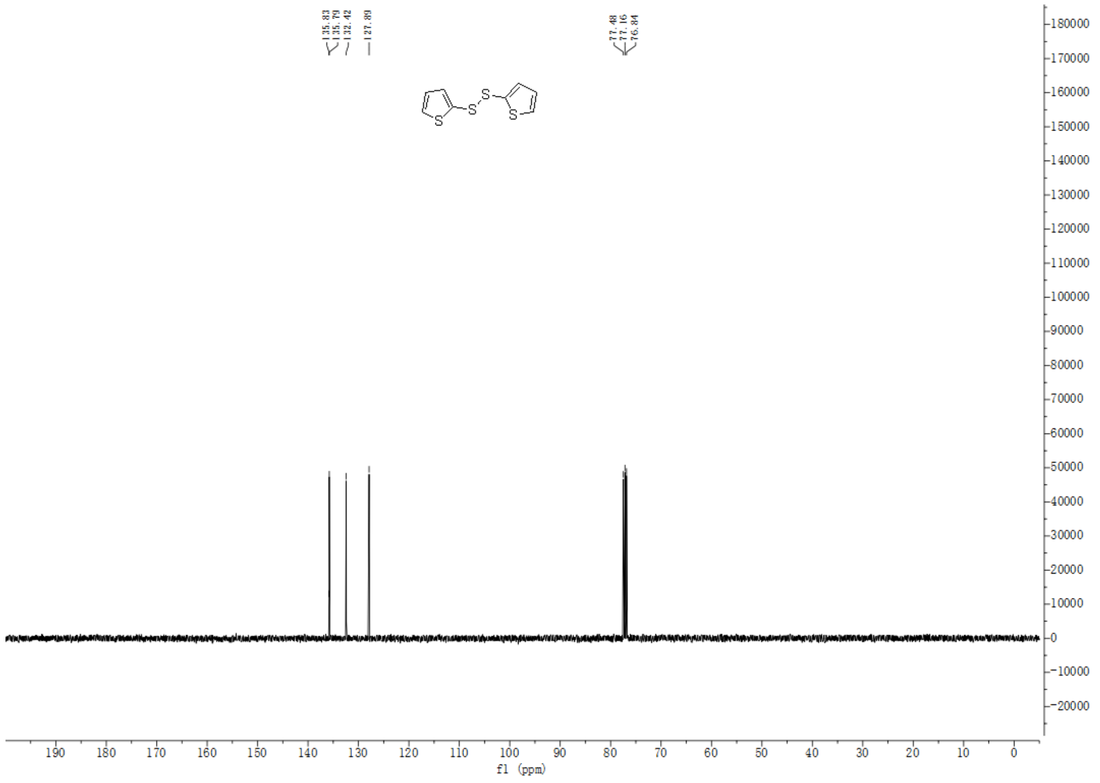

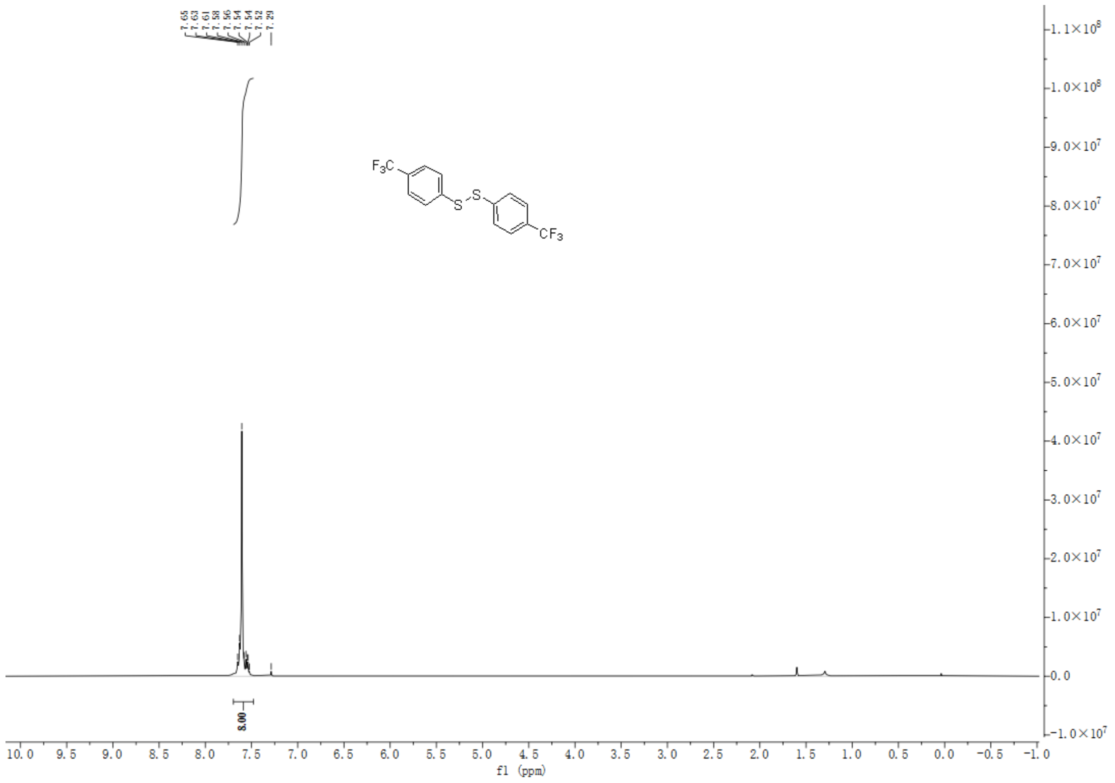

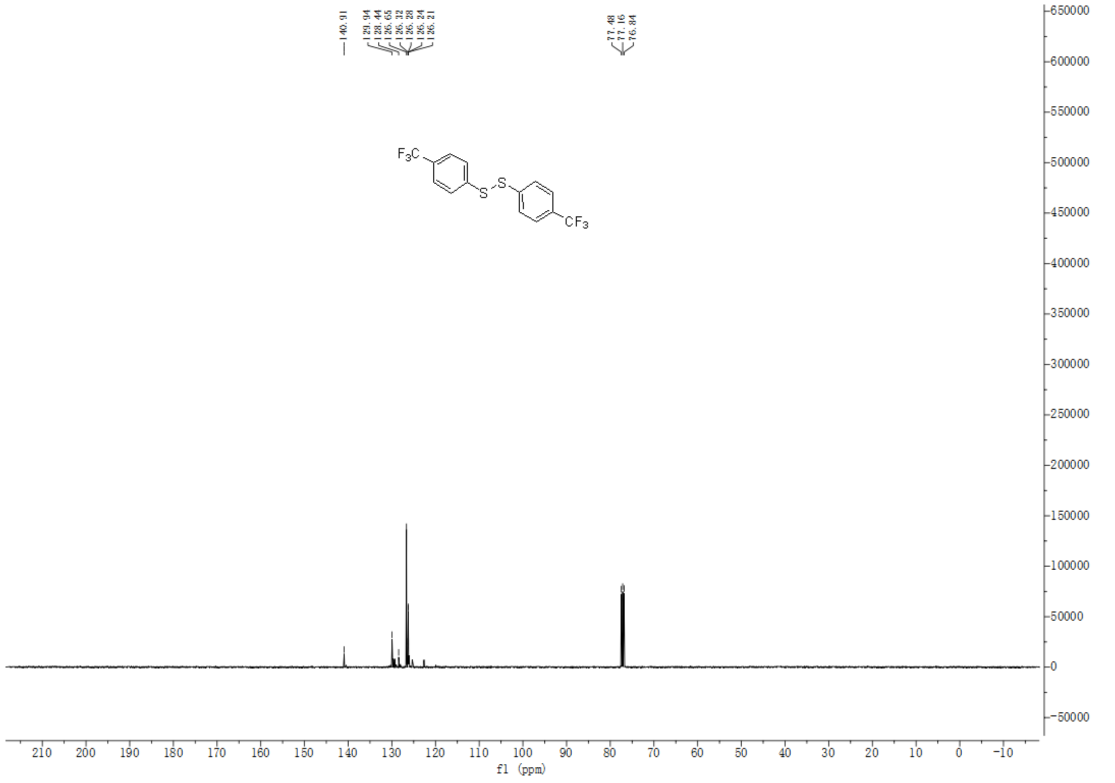

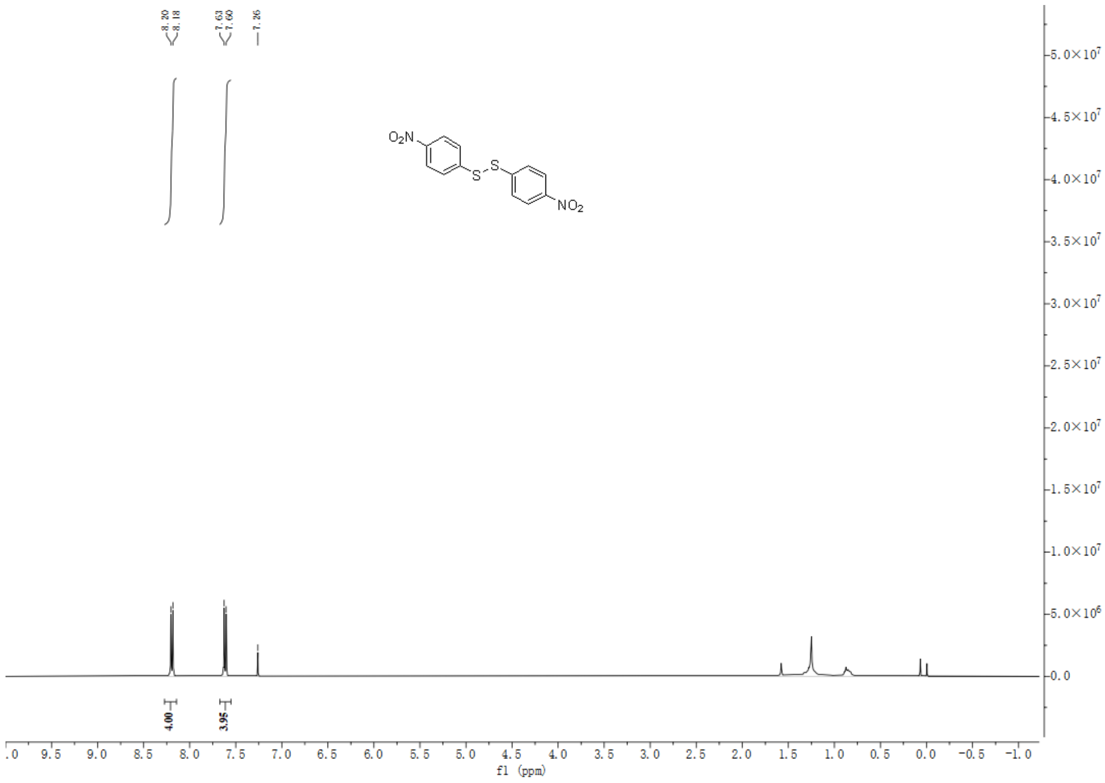

8.20
8.18
7.63
7.60
7.26
4.00
3.95
f1 (ppm)

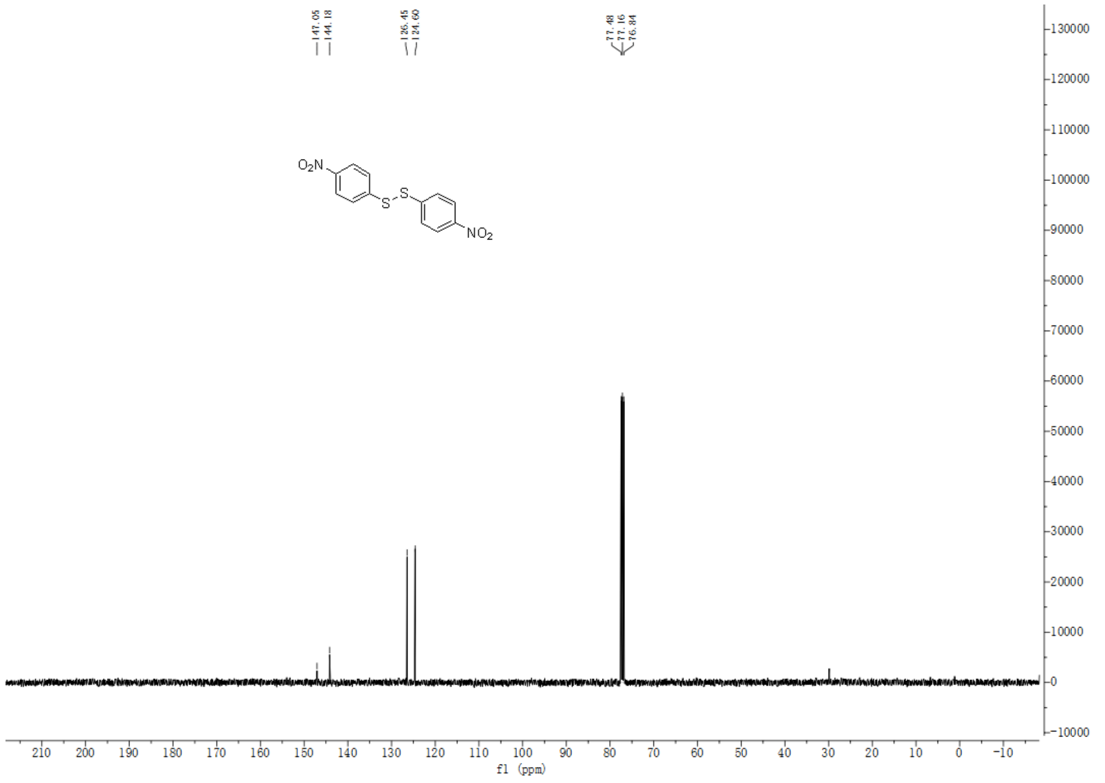

147.05
144.18
126.45
124.60
77.48
77.16
76.84
f1 (ppm)

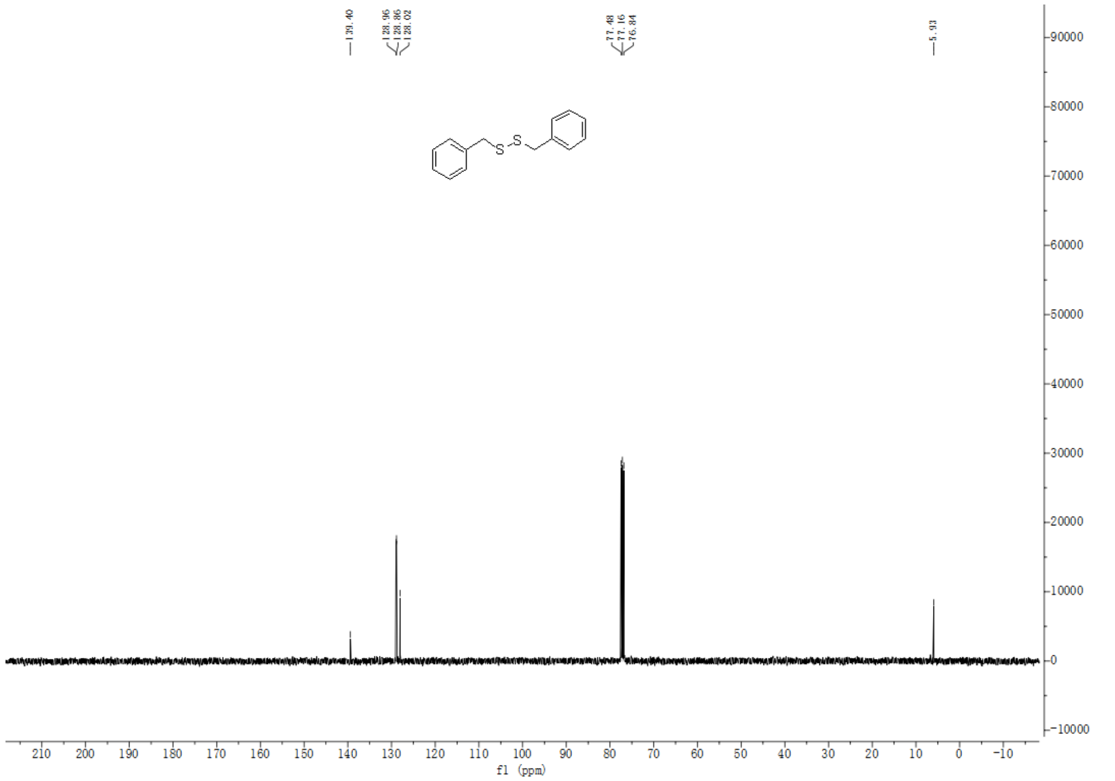
139.40
128.96
128.86
128.02
77.48
77.16
76.84
5.93
f1 (ppm)

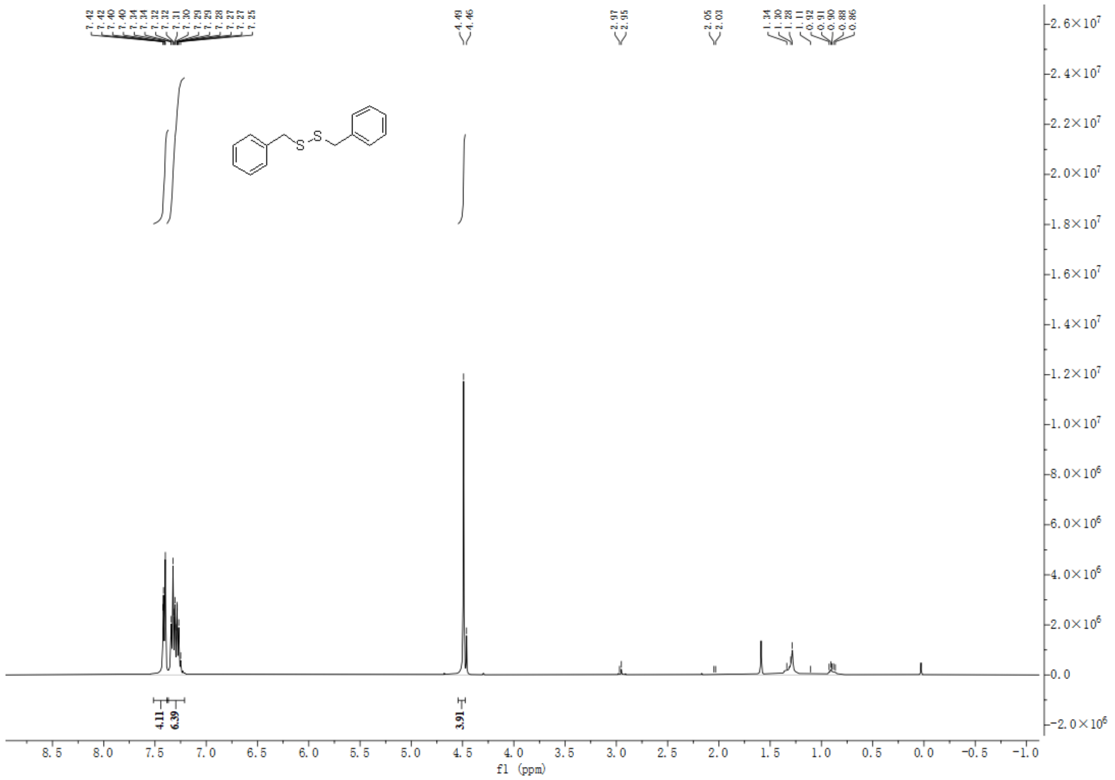
7.42
7.42
7.40
7.34
7.34
7.32
7.32
7.31
7.30
7.29
7.29
7.28
7.27
7.27
7.25
4.49
4.46
2.97
2.95
2.05
2.03
1.34
1.30
1.28
1.11
0.92
0.91
0.90
0.88
0.86
4.11
6.39
3.91
f1 (ppm)

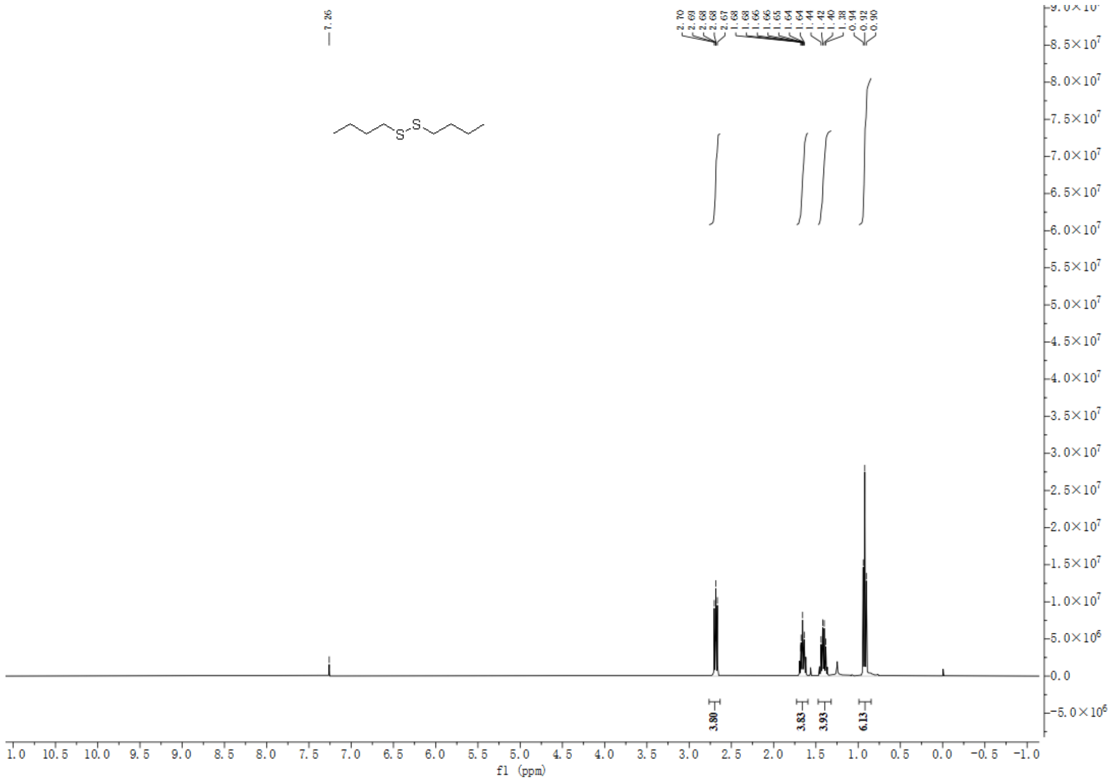

7.26
2.70
2.69
2.68
2.68
2.67
1.68
1.68
1.66
1.66
1.65
1.64
1.64
1.64
1.44
1.42
1.40
1.38
0.94
0.92
0.90
3.80
3.83
3.93
6.13
f1 (ppm)

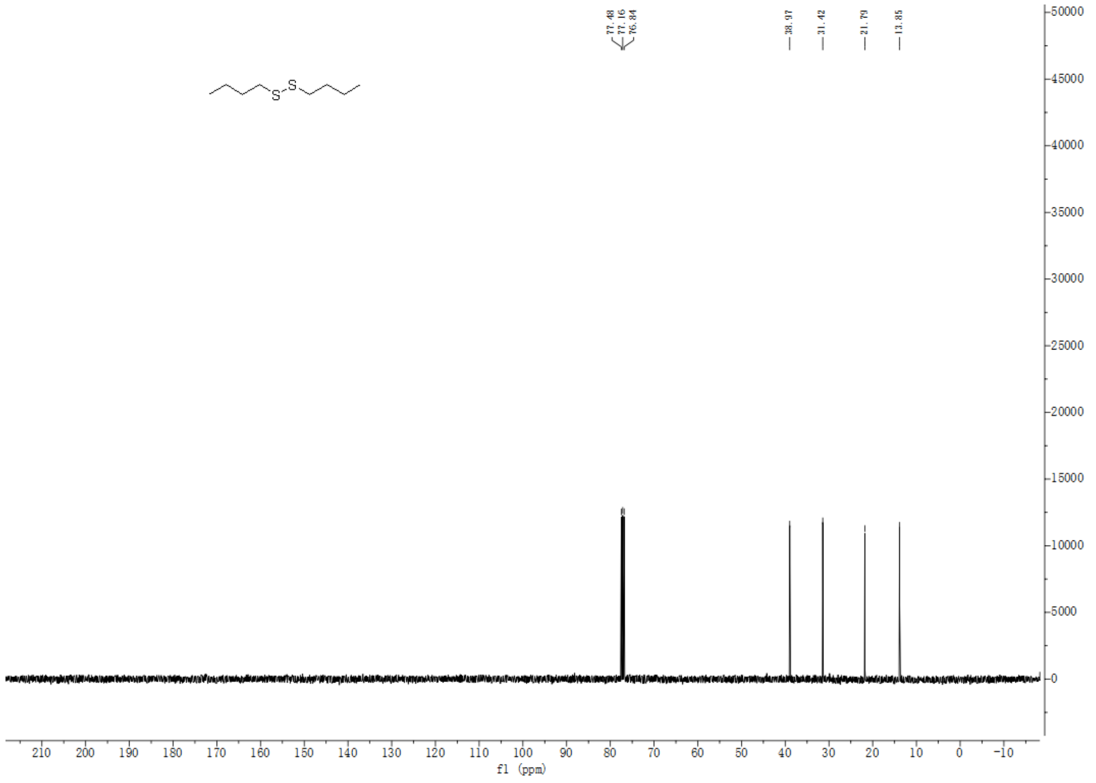

77.48
77.16
76.84
38.97
31.42
21.79
13.85
f1 (ppm)

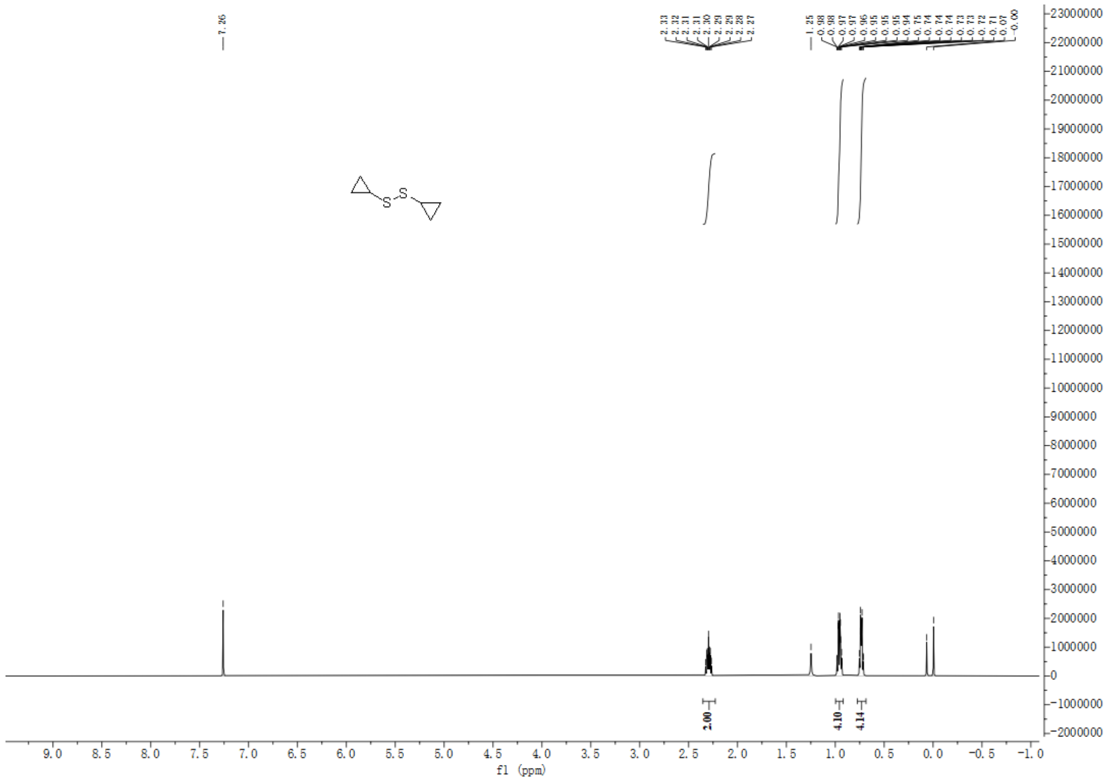
7.26
2.33
2.32
2.31
2.31
2.30
2.29
2.29
2.28
2.27
1.25
0.98
0.98
0.97
0.97
0.96
0.95
0.95
0.95
0.94
0.75
0.74
0.74
0.74
0.73
0.73
0.72
0.71
0.07
0.00
2.00
4.10
4.14
f1 (ppm)

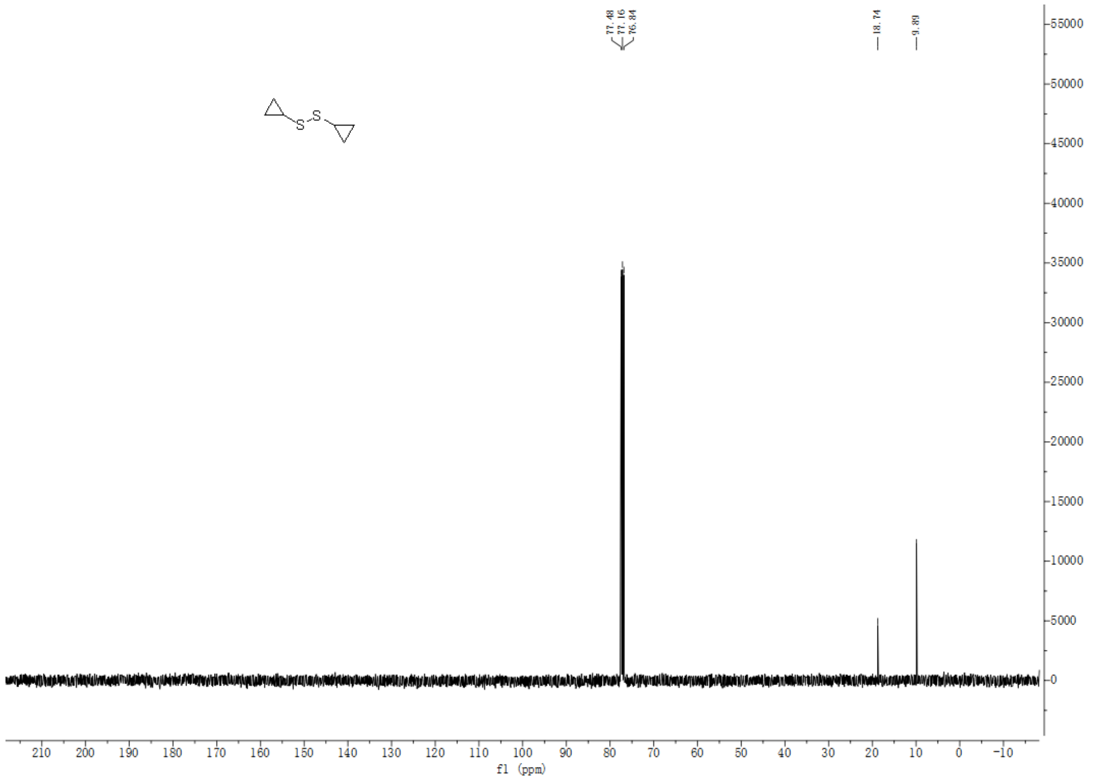
77.48
77.16
76.84
18.74
9.89
f1 (ppm)

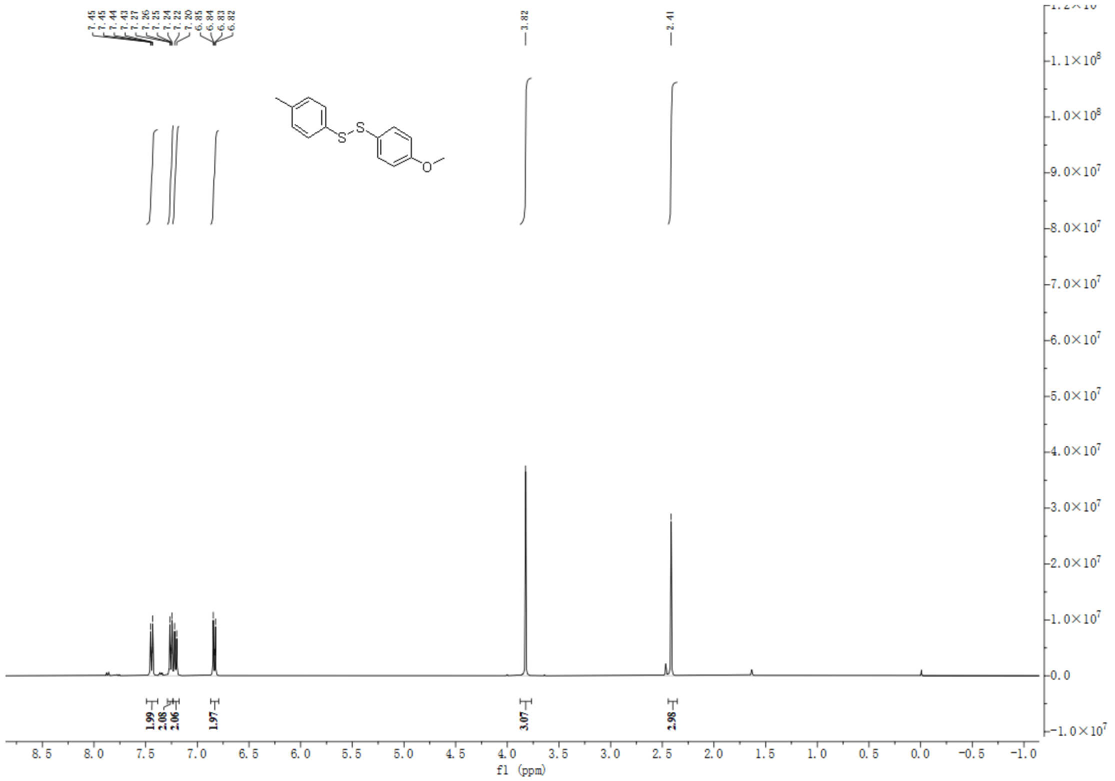
7.45
7.45
7.44
7.43
7.27
7.26
7.25
7.24
7.22
7.20
6.85
6.84
6.83
6.82
3.82
2.41
1.99
2.08
2.06
1.97
3.07
2.98
S
S
O
f1 (ppm)

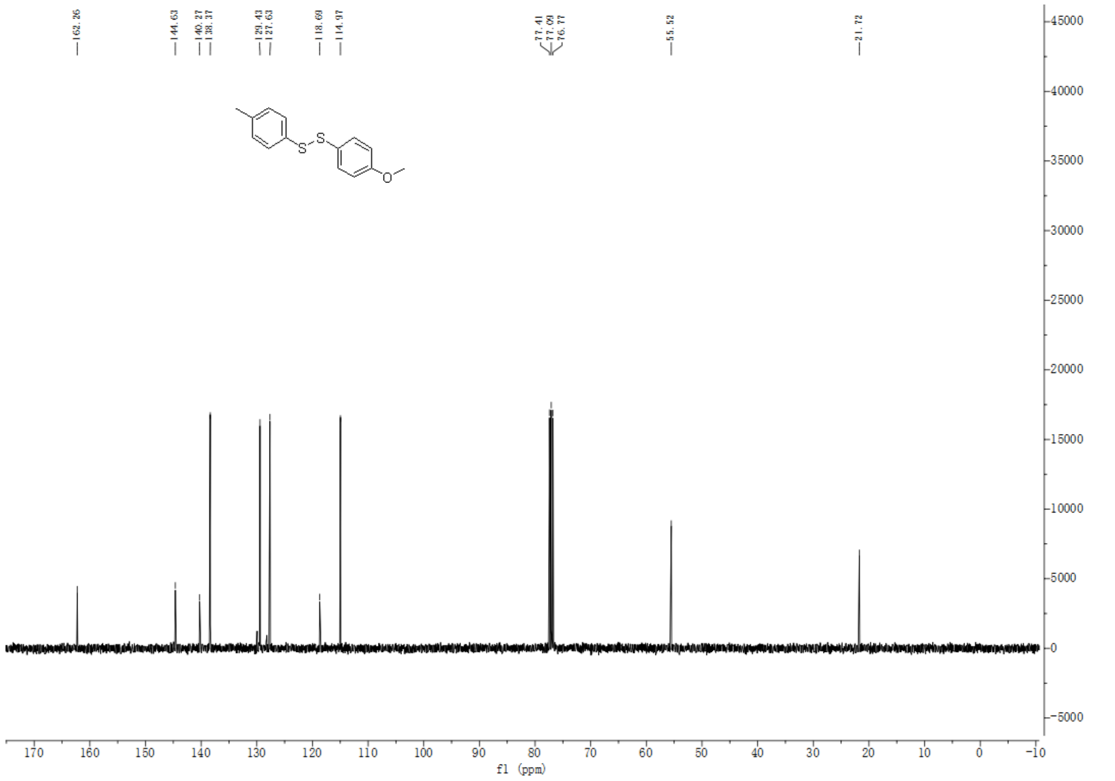
162.26
144.63
140.27
138.37
129.43
127.63
118.69
114.97
77.41
77.09
76.77
55.52
21.72
S
S
O
f1 (ppm)

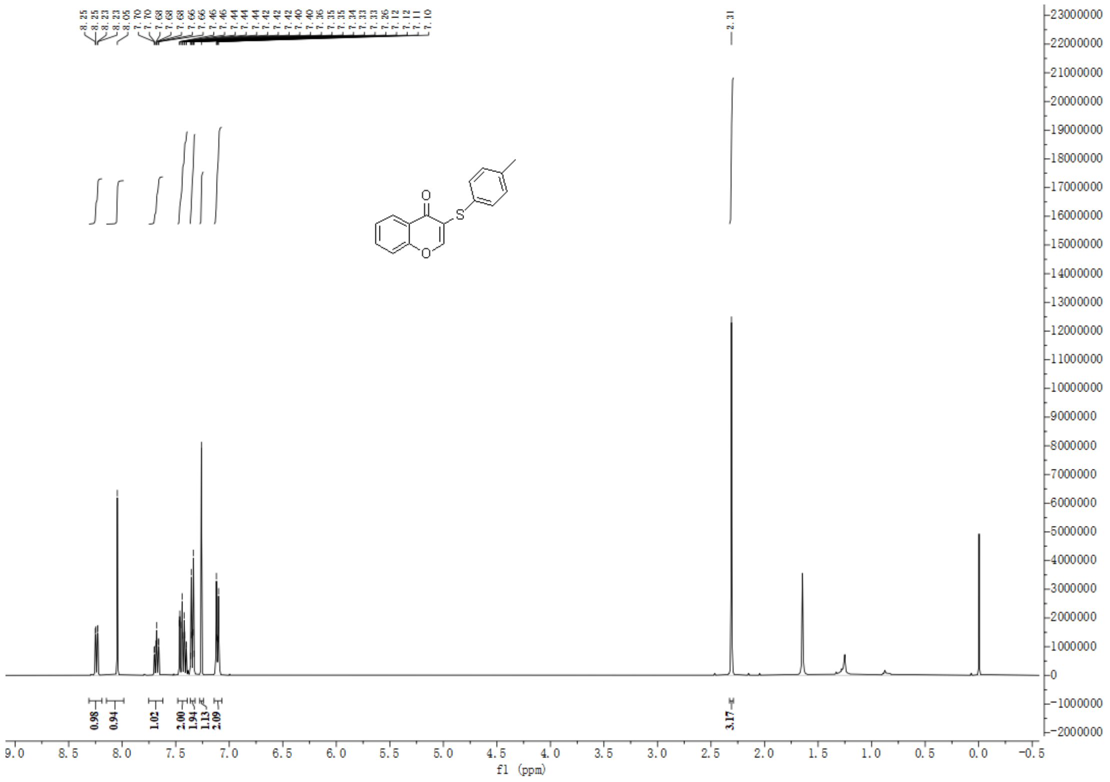

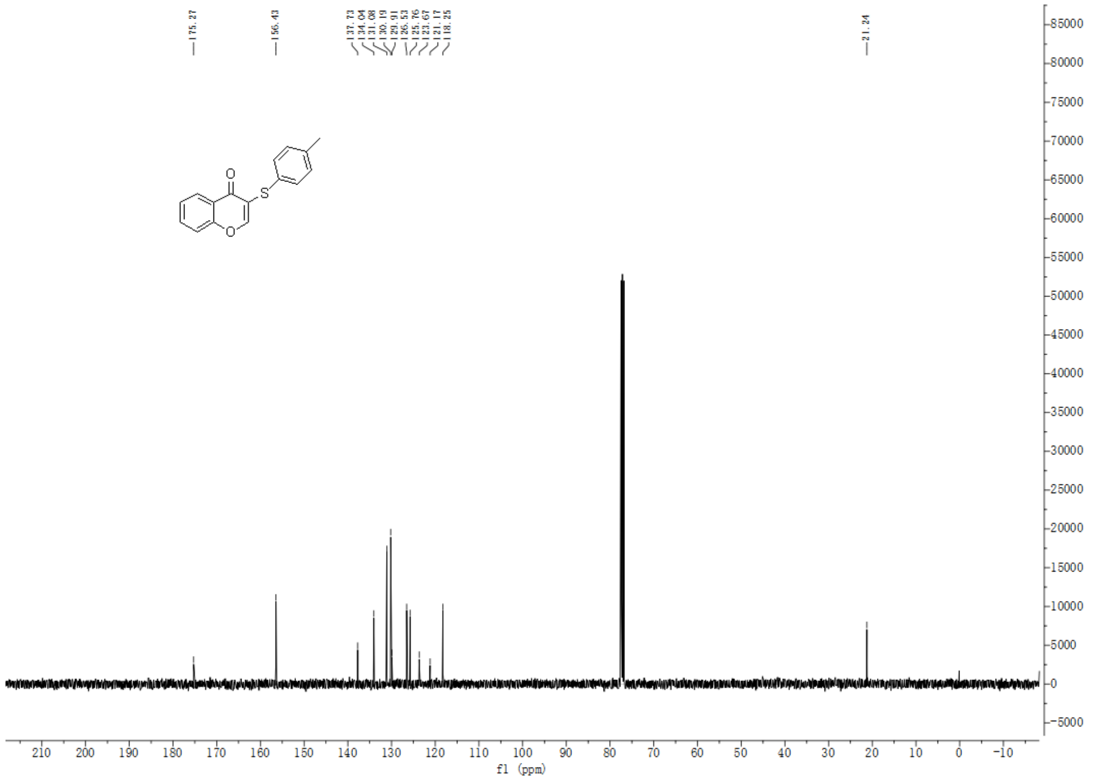

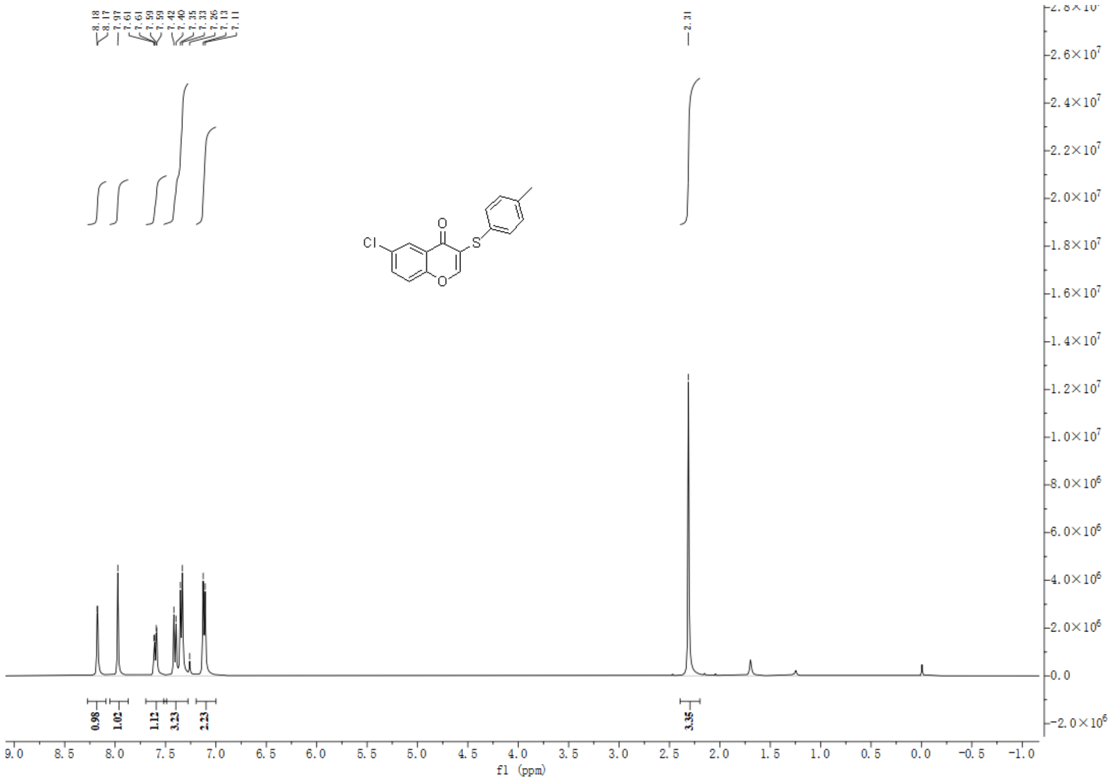
8.18
8.17
7.97
7.61
7.61
7.59
7.59
7.42
7.40
7.35
7.33
7.26
7.13
7.11
2.31
Cl
O
S
0.98
1.02
1.12
3.23
2.23
3.35
f1 (ppm)

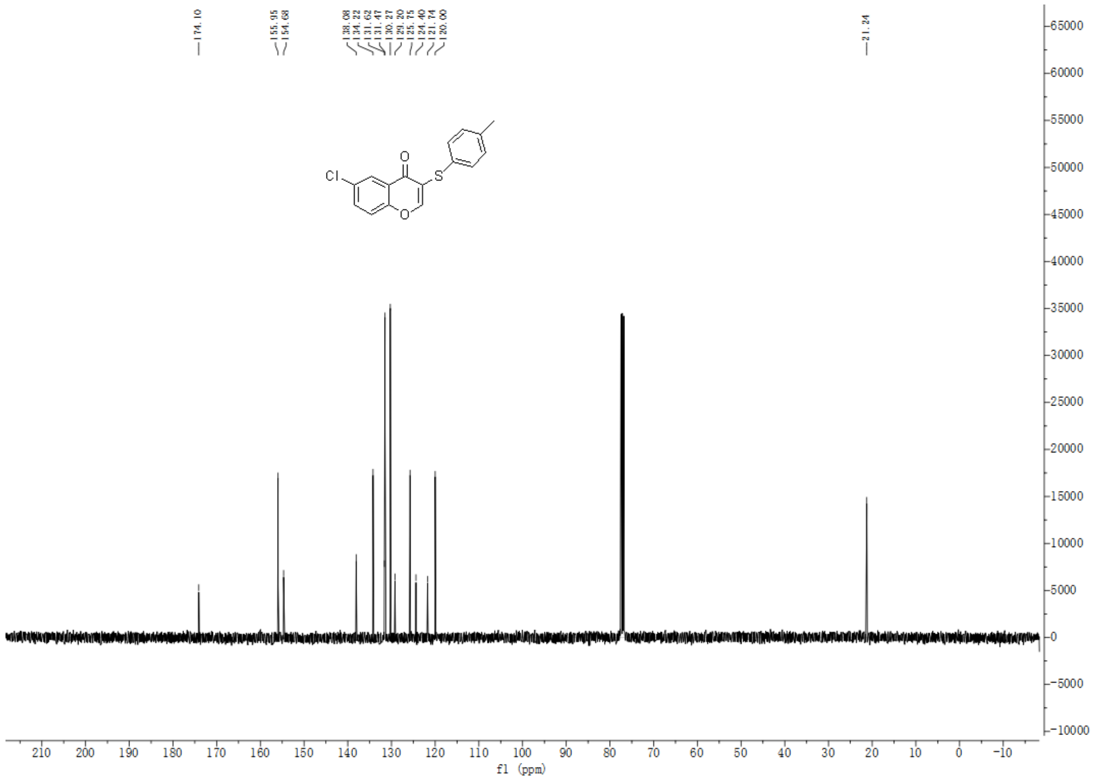
174.10
155.95
154.68
138.08
134.22
131.62
131.47
130.27
129.20
125.75
124.40
121.74
120.00
21.24
Cl
O
S
f1 (ppm)

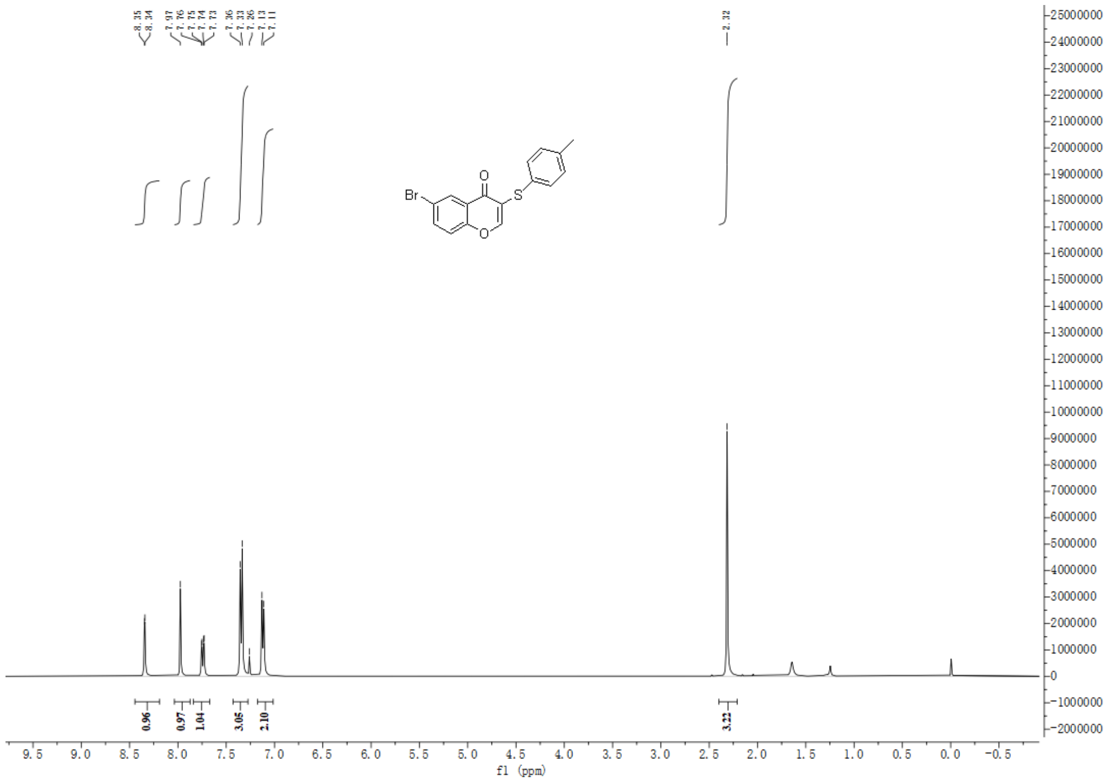
8.35
8.34
7.97
7.76
7.75
7.74
7.73
7.36
7.33
7.26
7.13
7.11
2.32
Br
O
S
O
0.96
0.97
1.04
3.05
2.10
3.22
f1 (ppm)

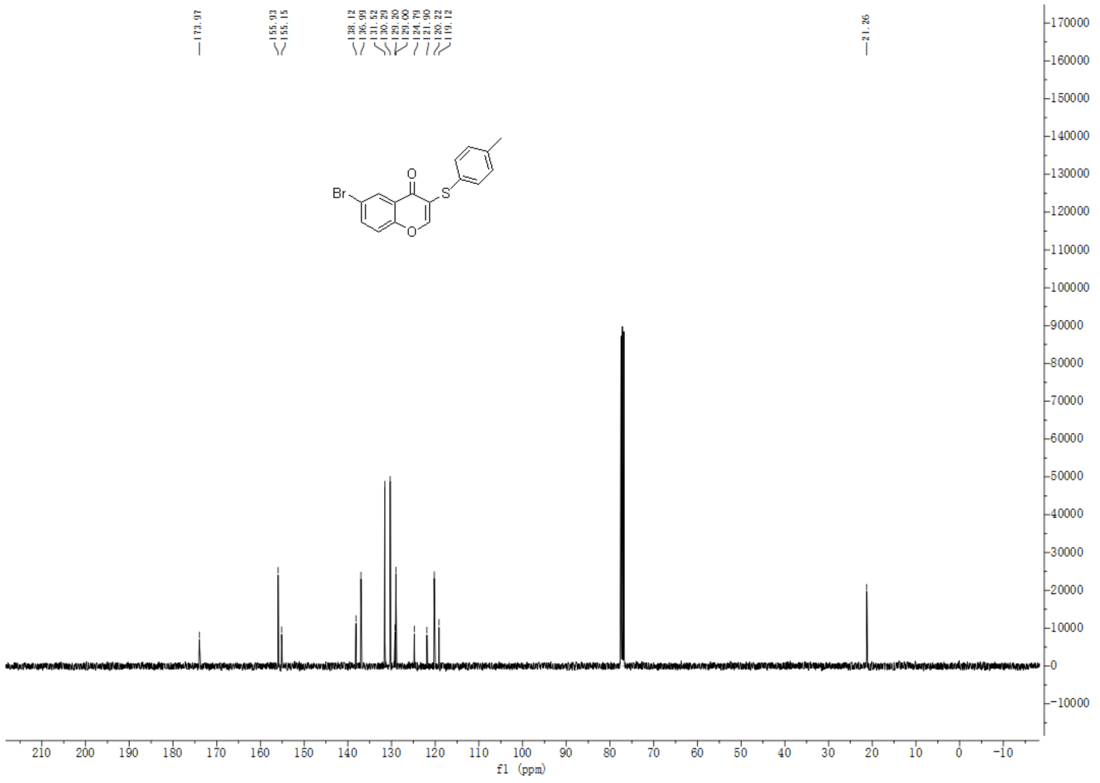
173.97
155.93
155.15
138.12
136.99
131.52
130.29
129.20
129.00
124.79
121.90
120.22
119.12
21.26
Br
O
S
O
f1 (ppm)

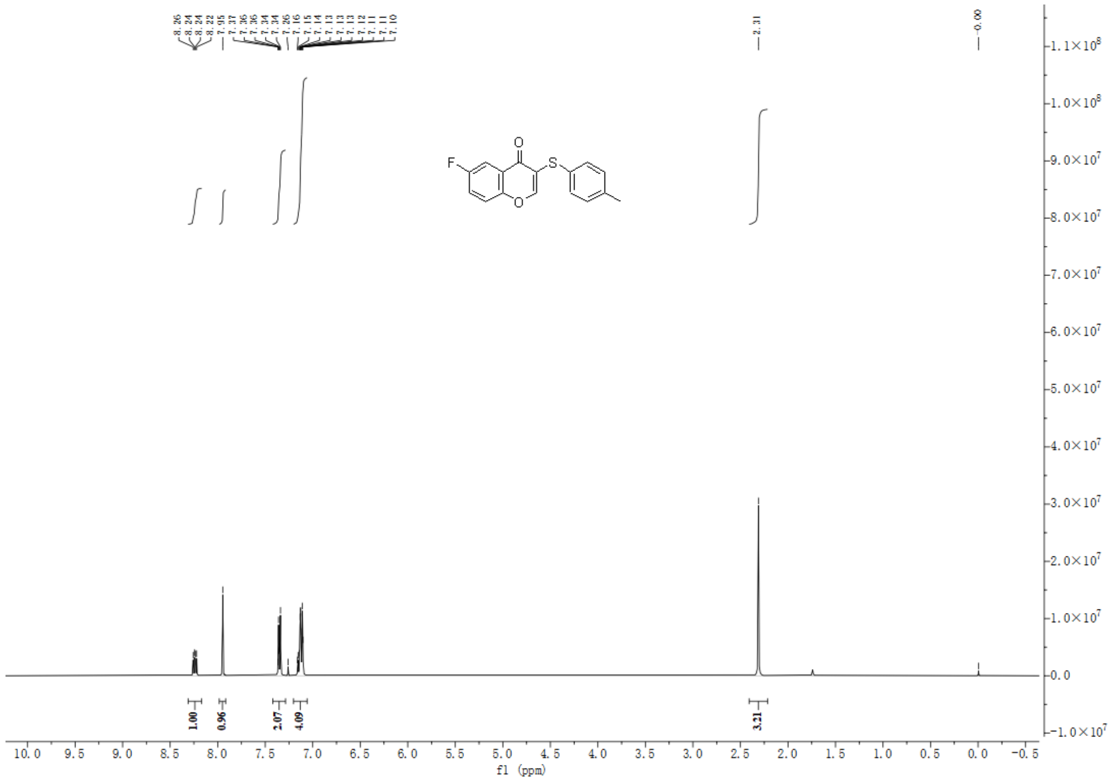

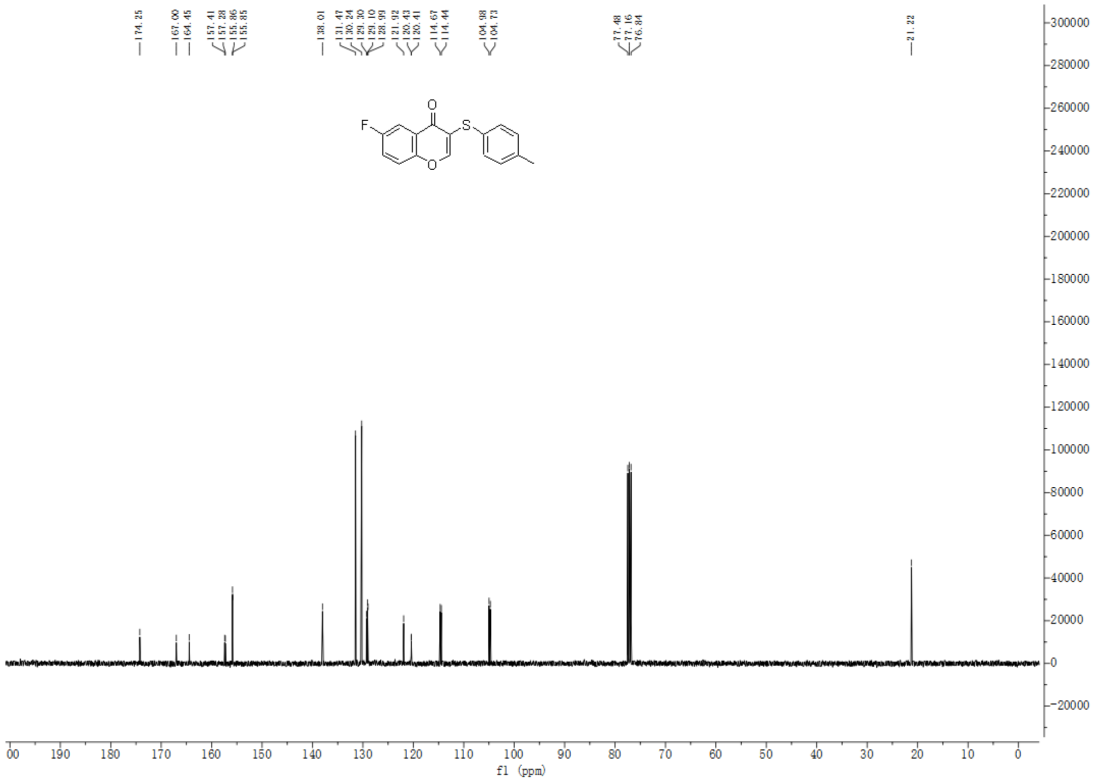

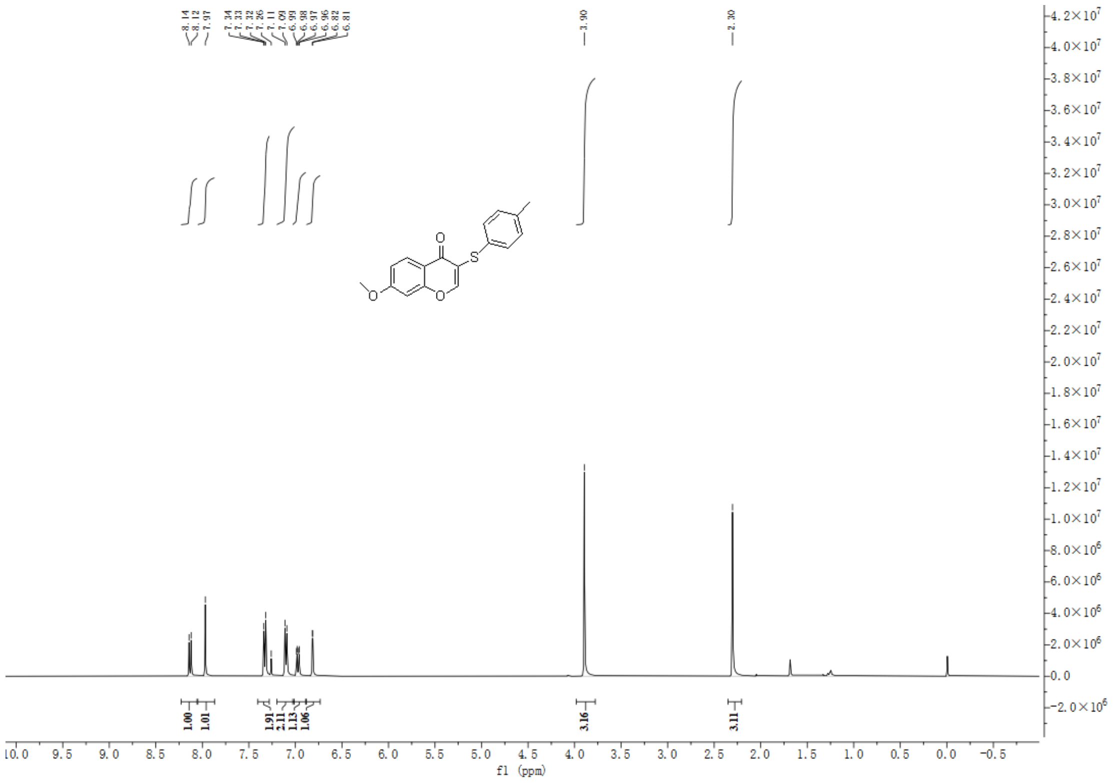

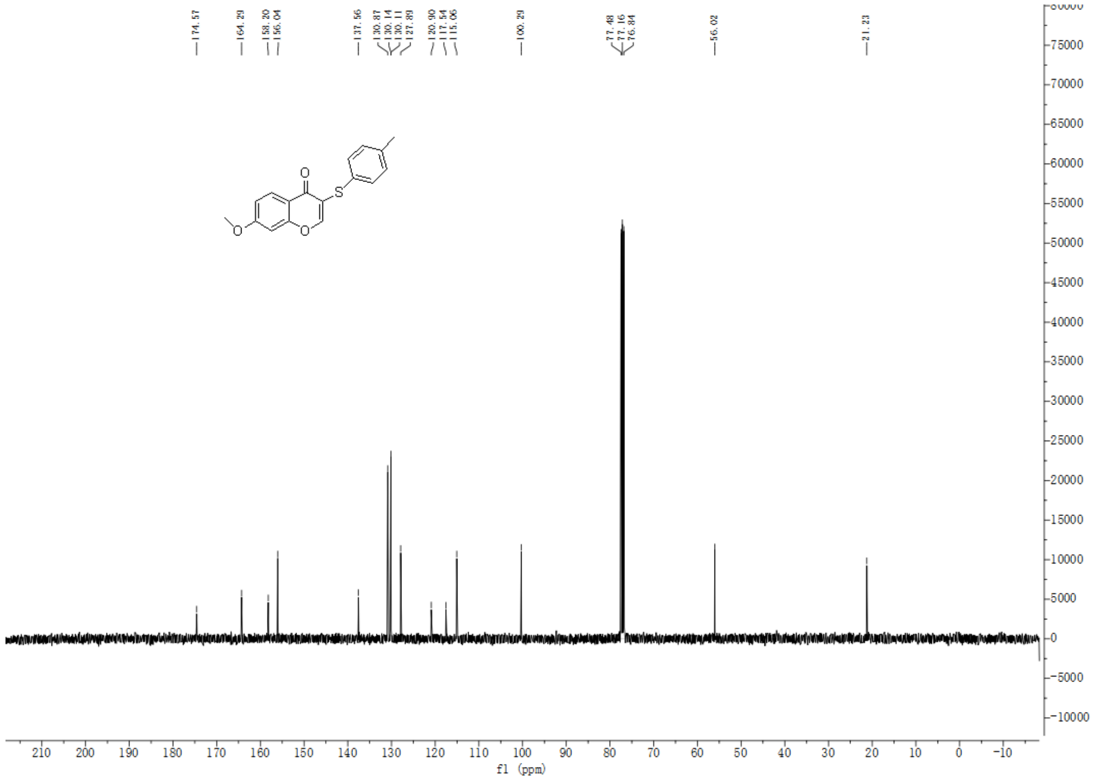

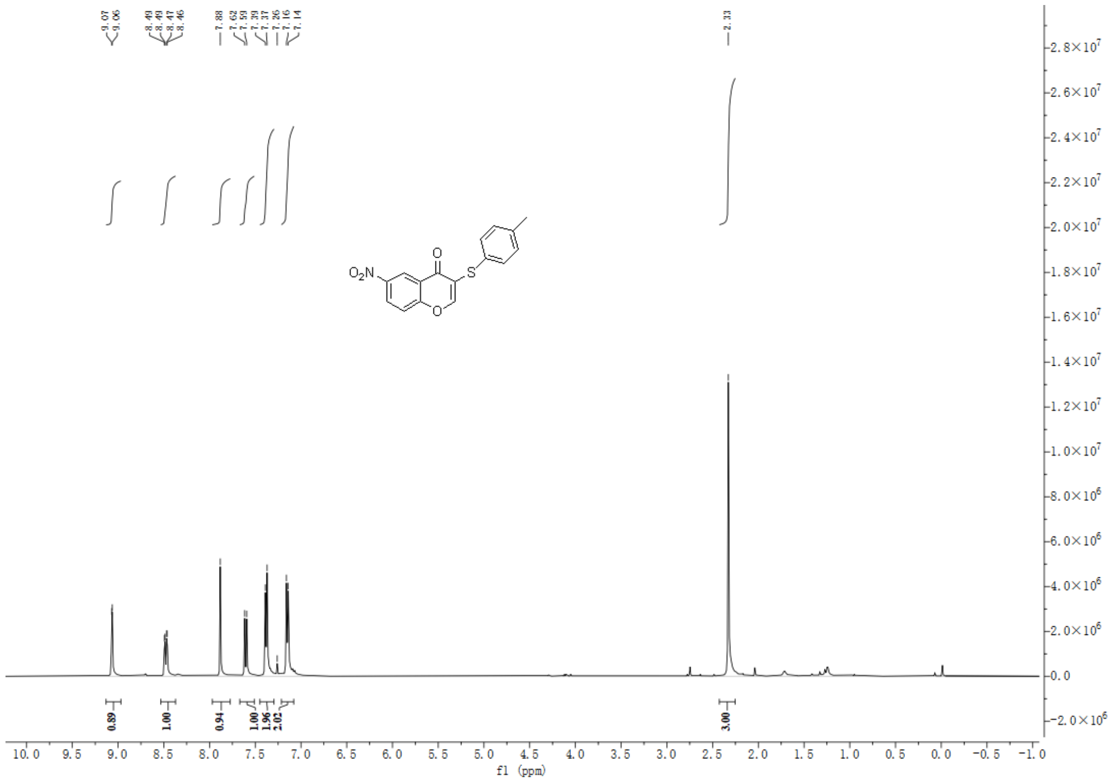

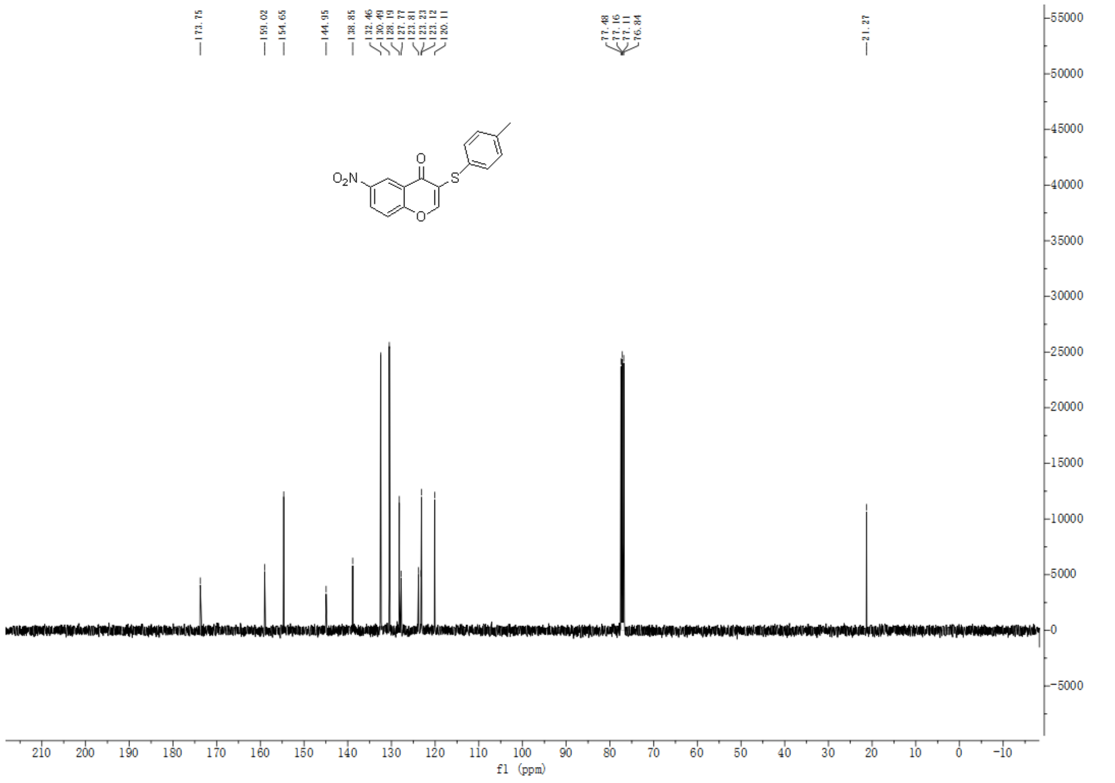

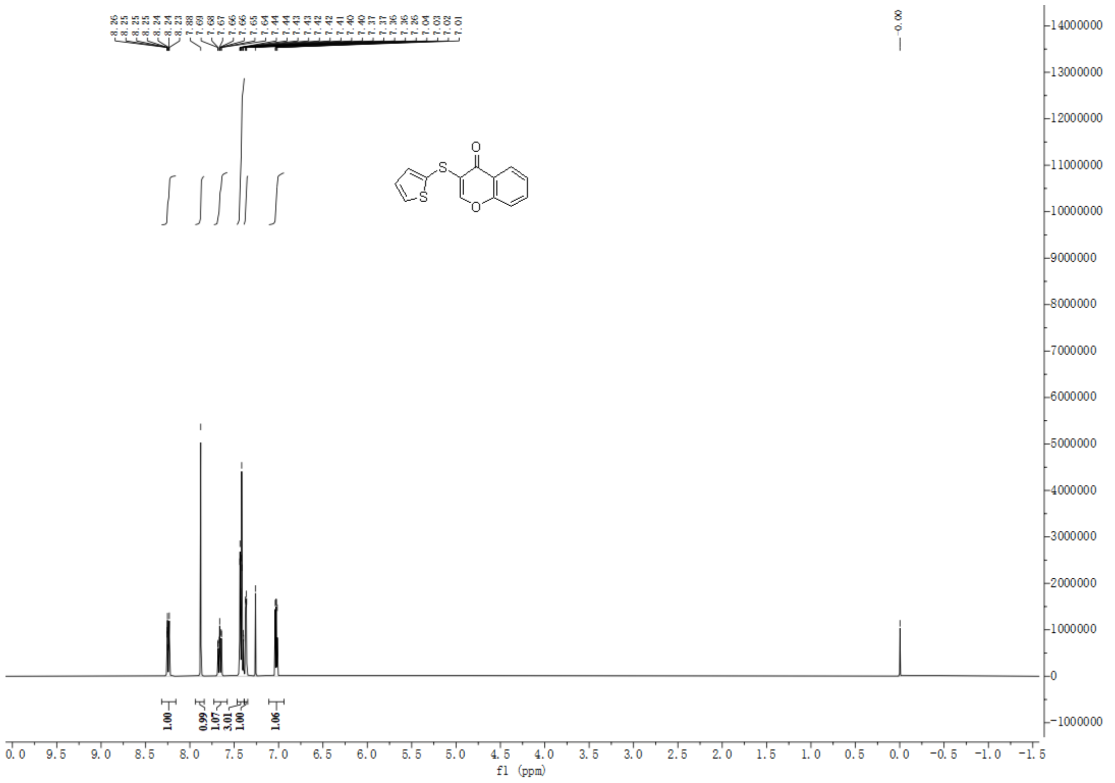

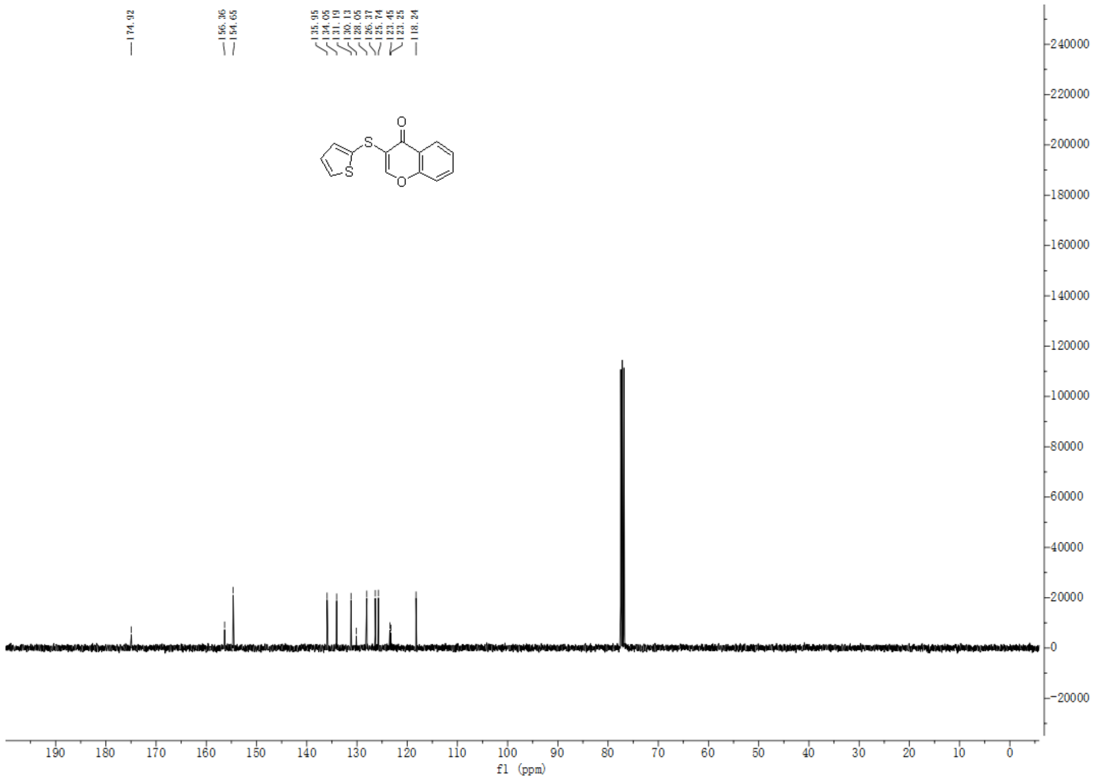

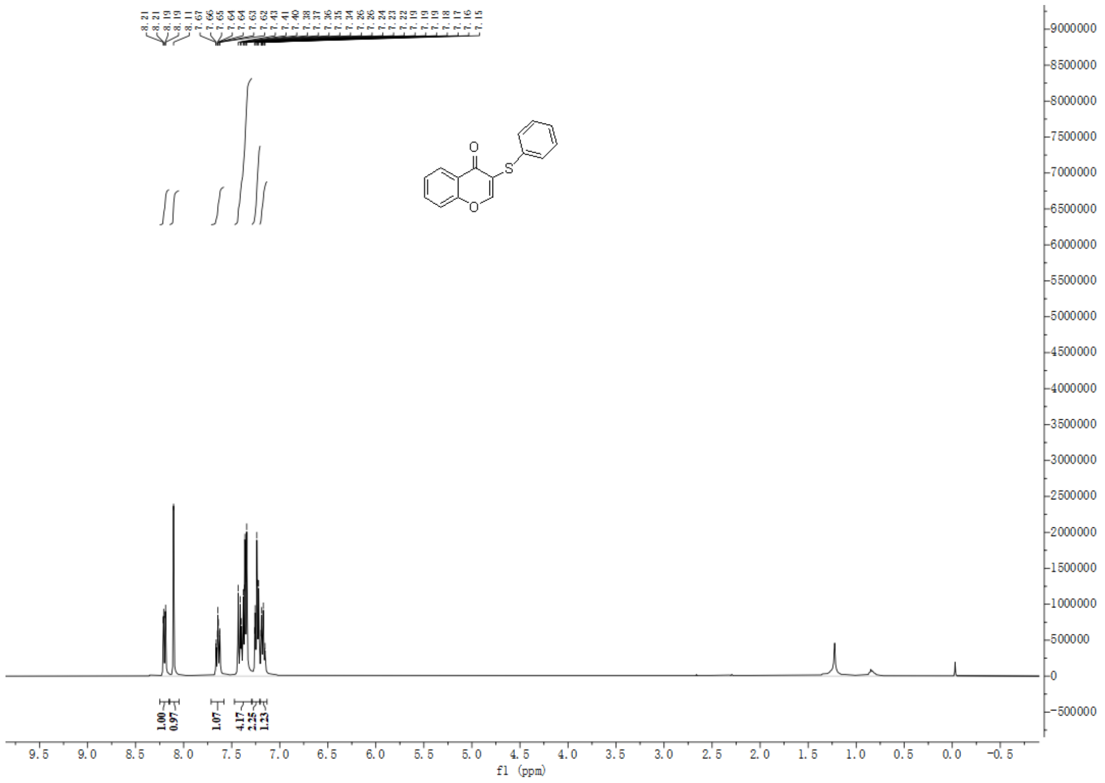

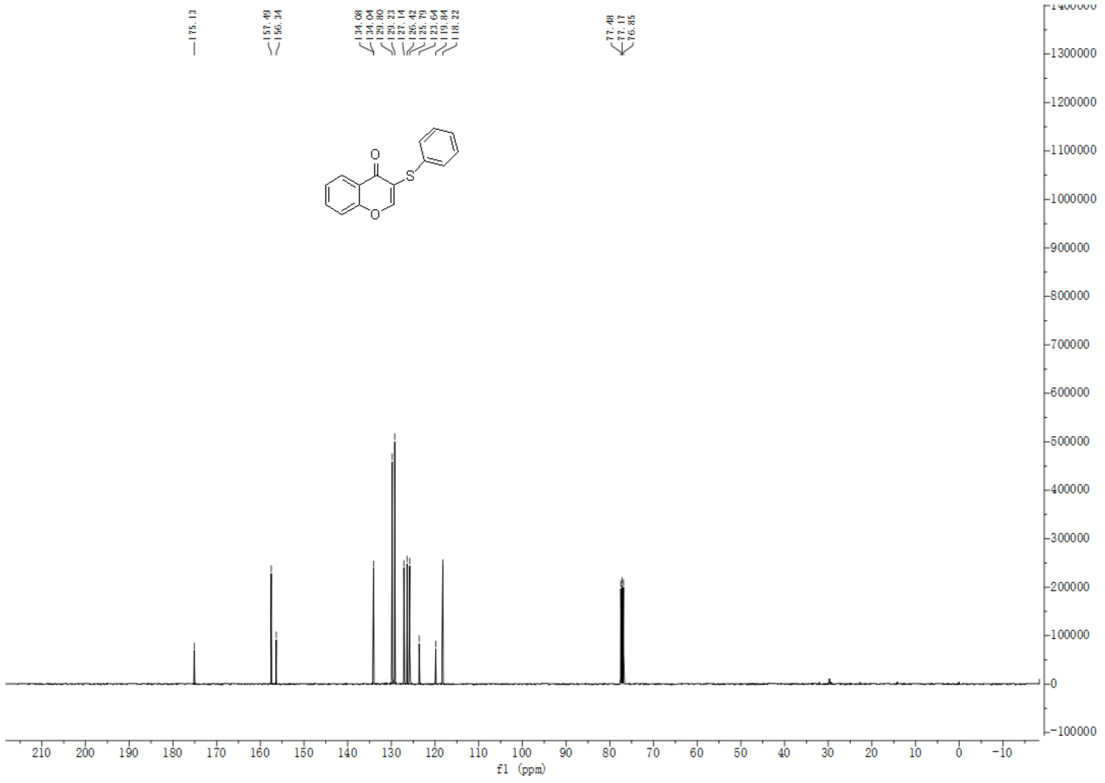

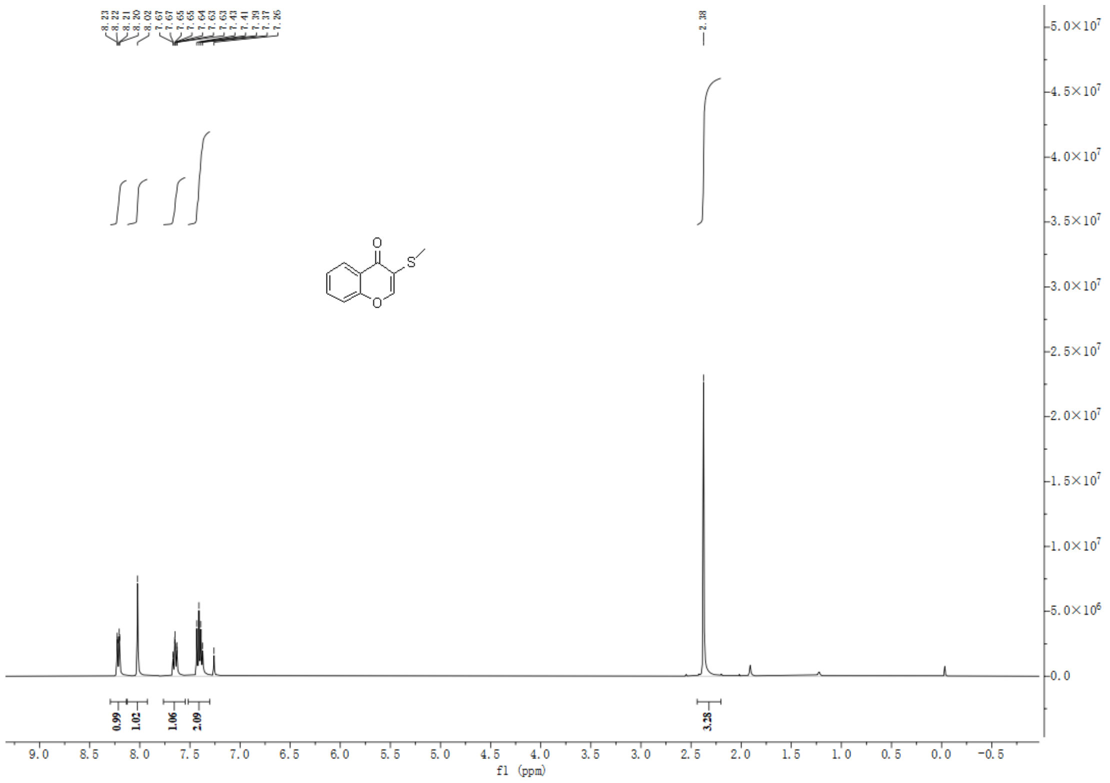
8.23
8.22
8.21
8.20
8.02
7.67
7.67
7.65
7.65
7.64
7.63
7.63
7.43
7.41
7.39
7.37
7.26
2.38
O
S
O
0.99
1.02
1.06
2.09
3.28
f1 (ppm)

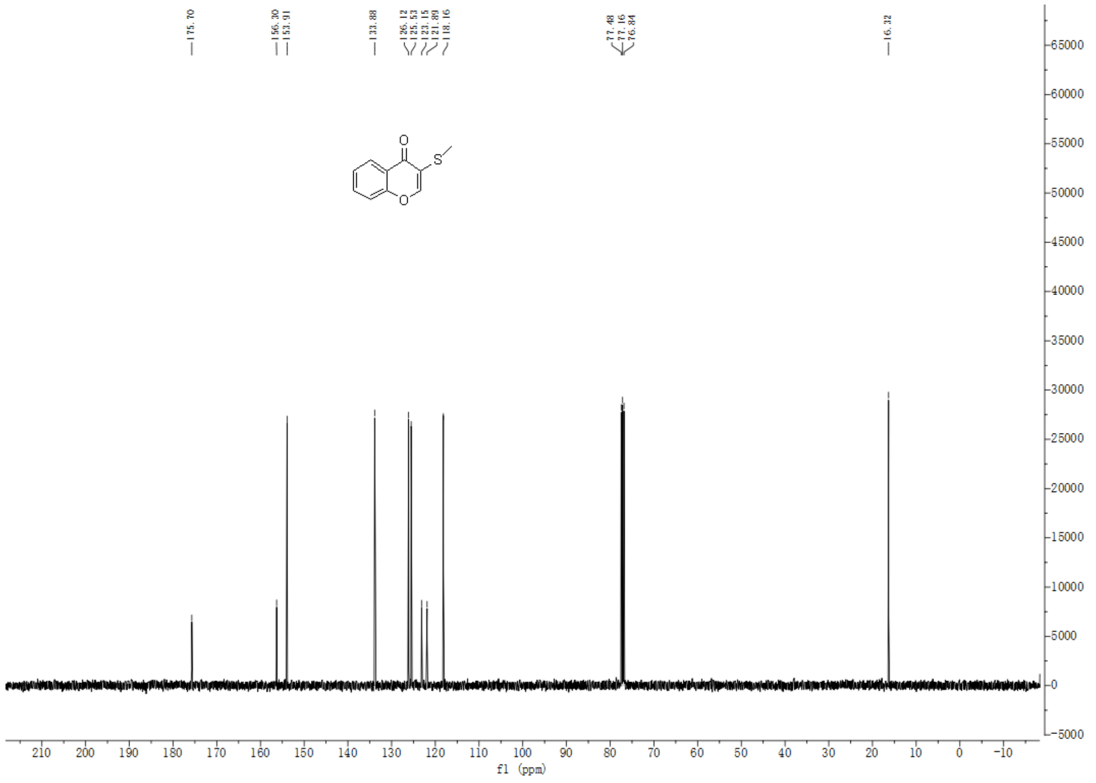
175.70
156.30
153.91
133.88
126.12
125.53
123.15
121.89
118.16
77.48
77.16
76.84
16.32
O
S
O
f1 (ppm)

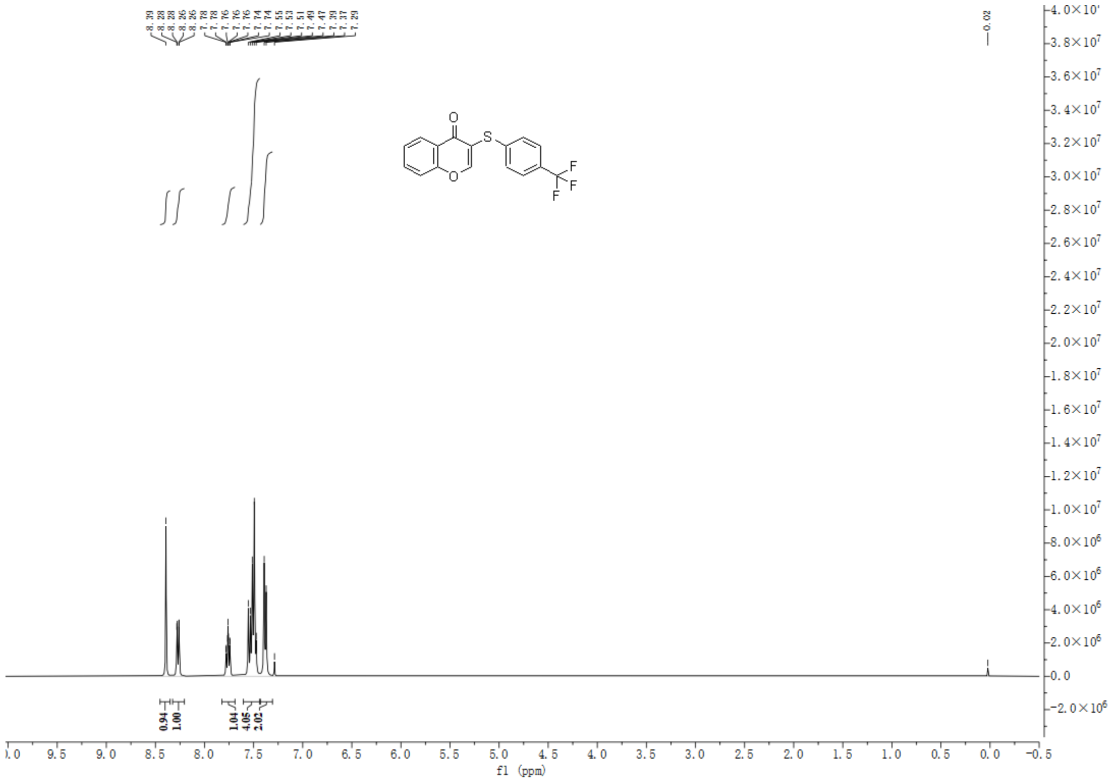

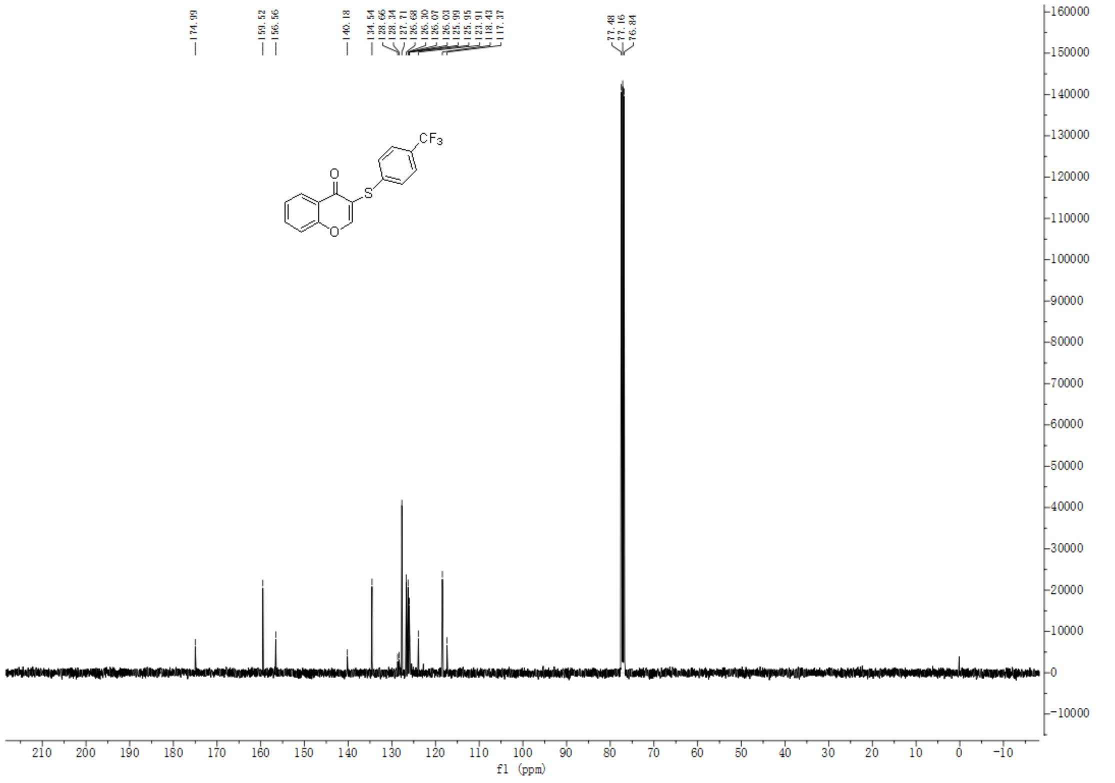

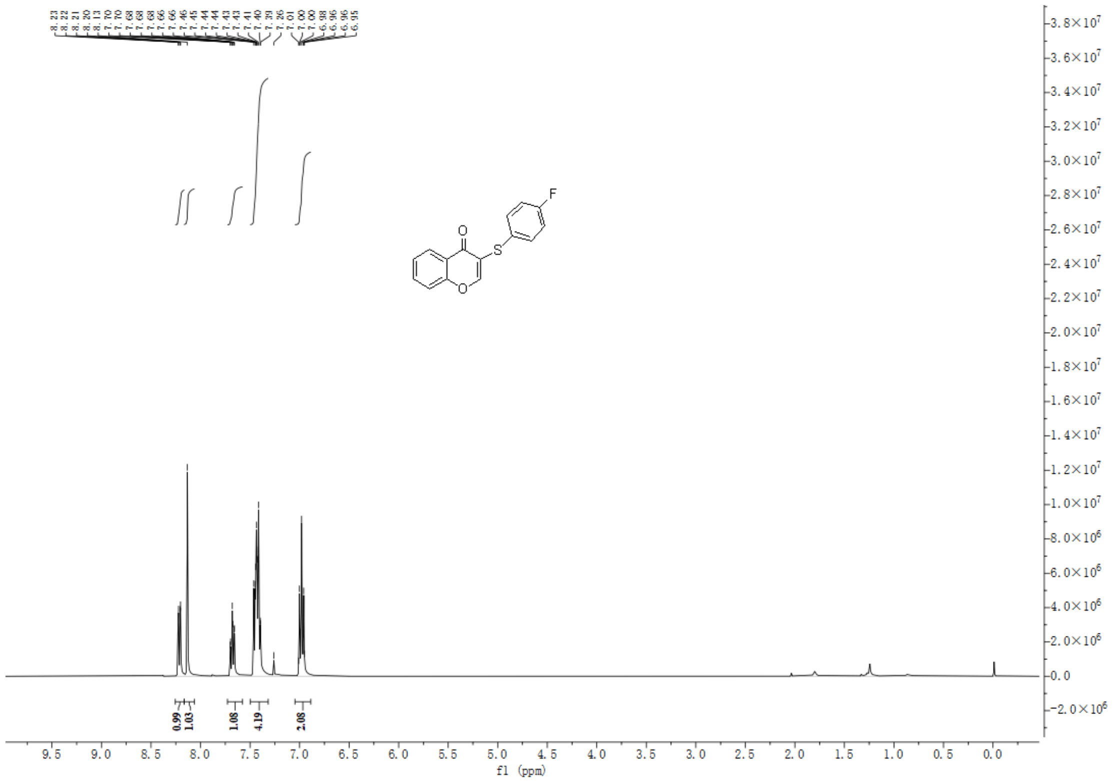
f1 (ppm)

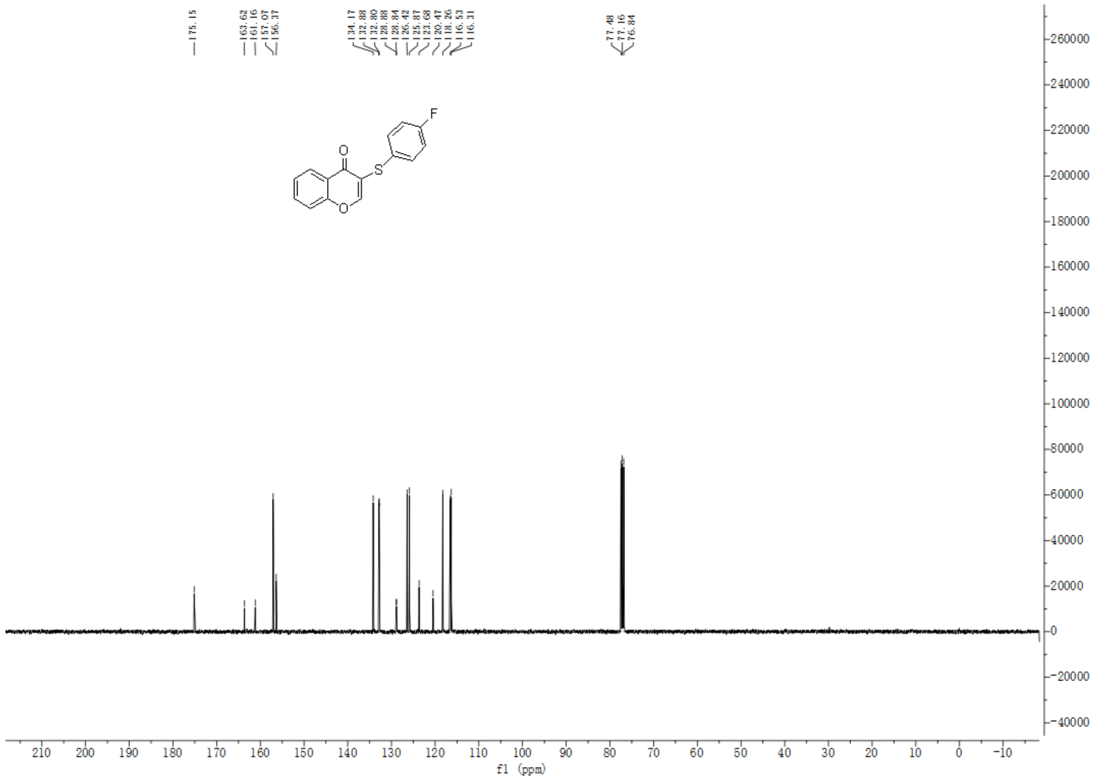
f1 (ppm)

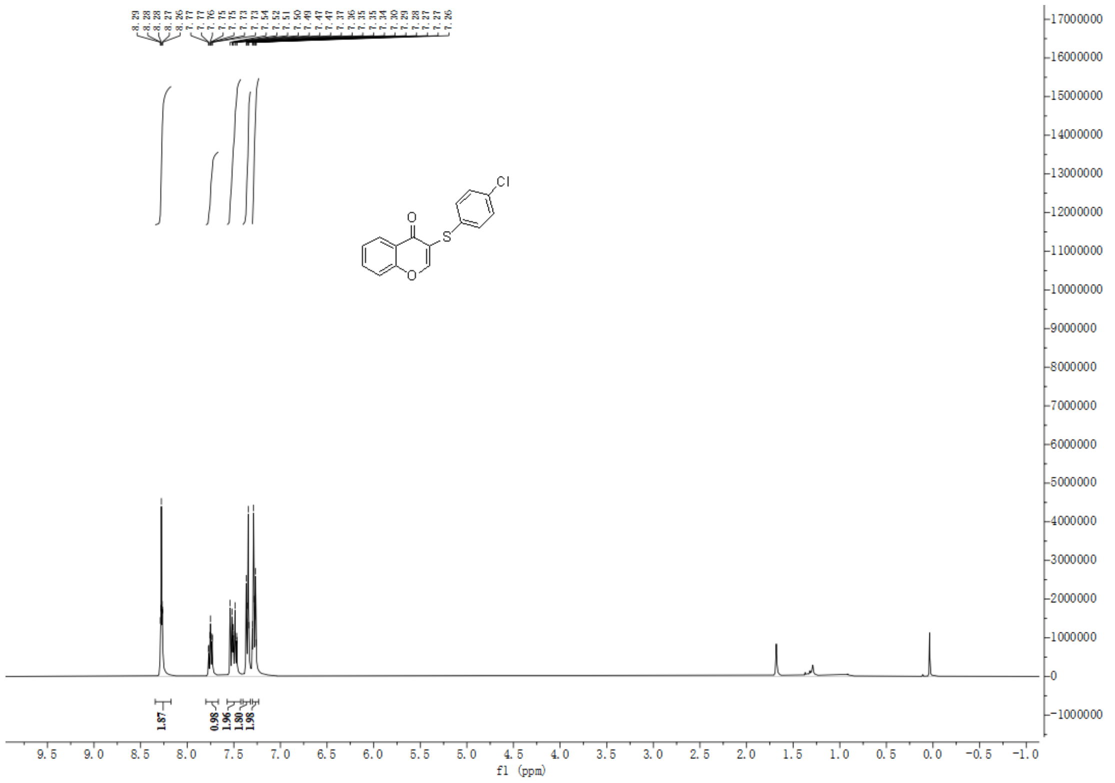

Cl
O
S
O
fl (ppm)

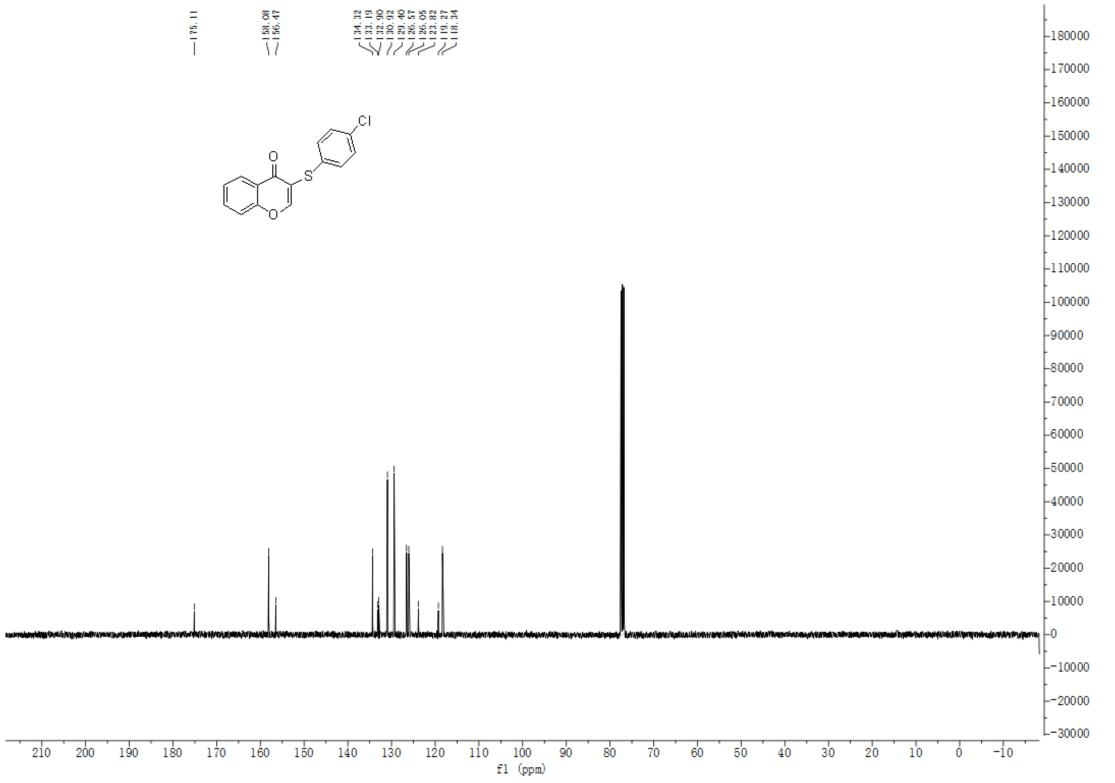

175.11
158.08
156.47
134.32
133.19
132.90
130.92
129.40
126.57
126.05
123.82
119.27
118.34
Cl
O
S
O
fl (ppm)

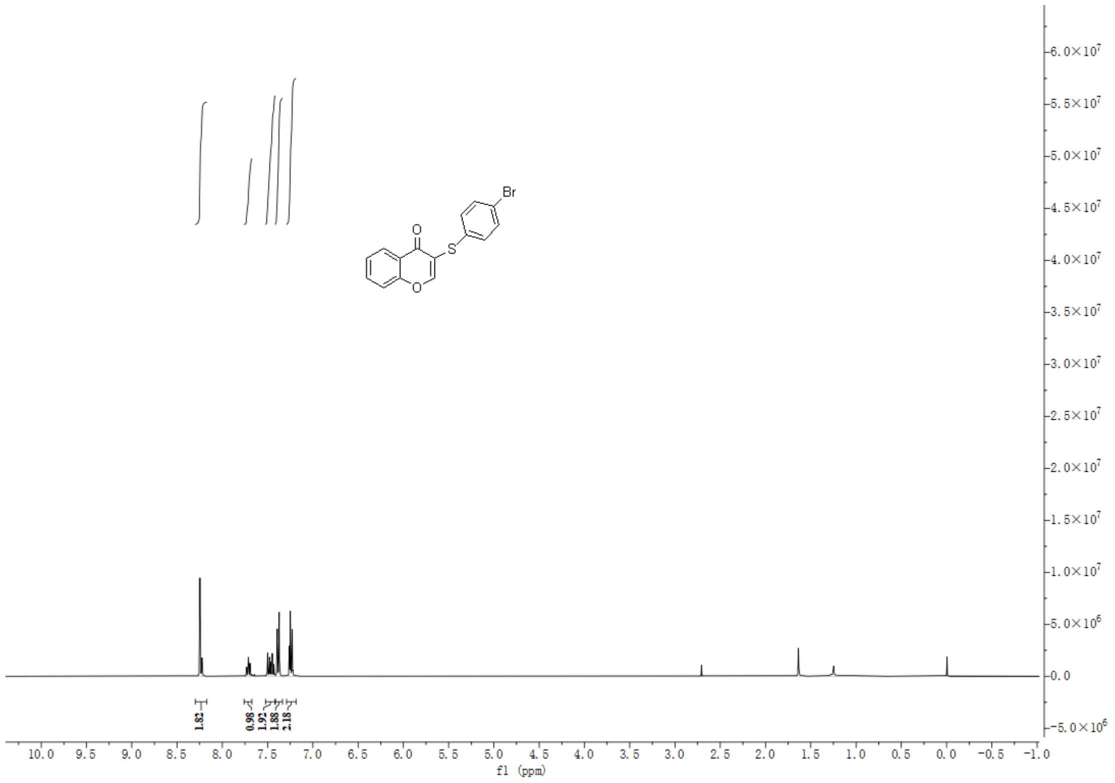

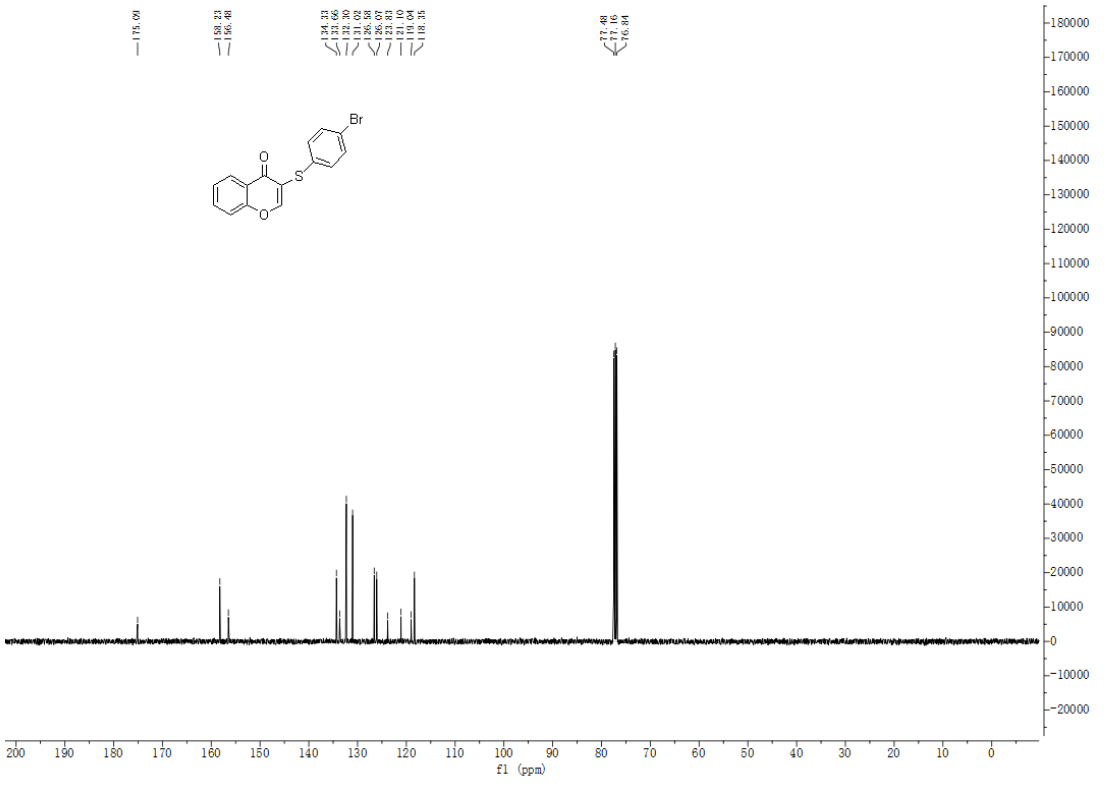

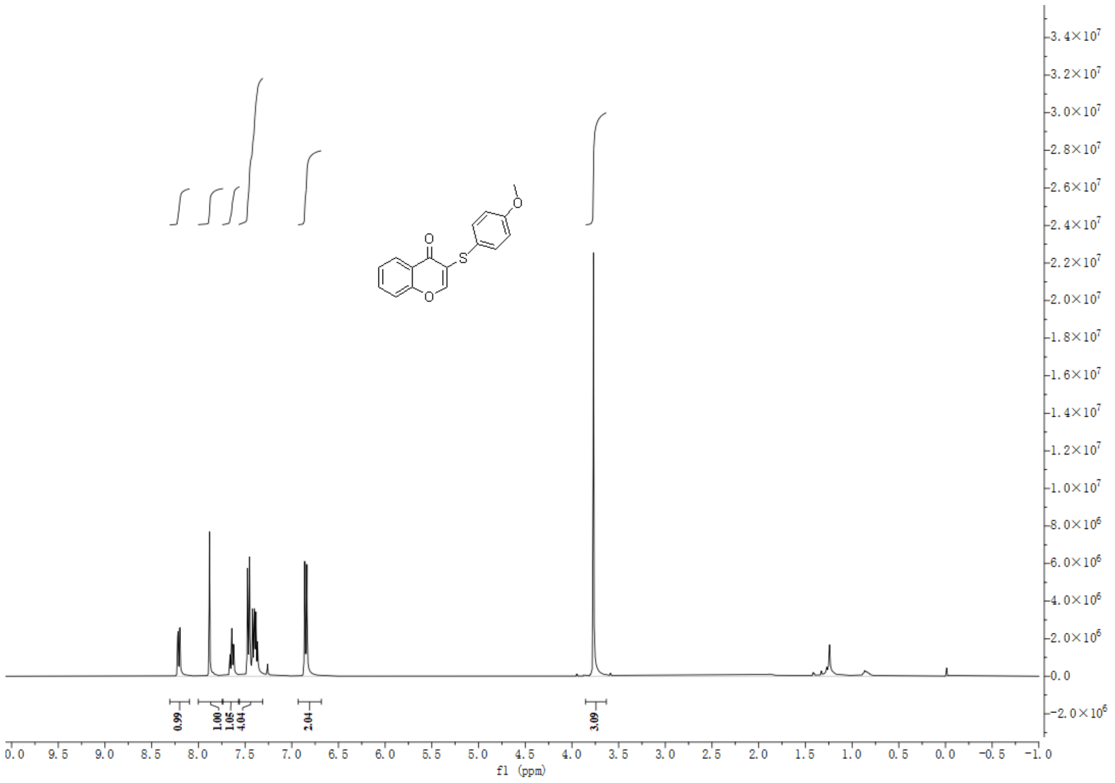

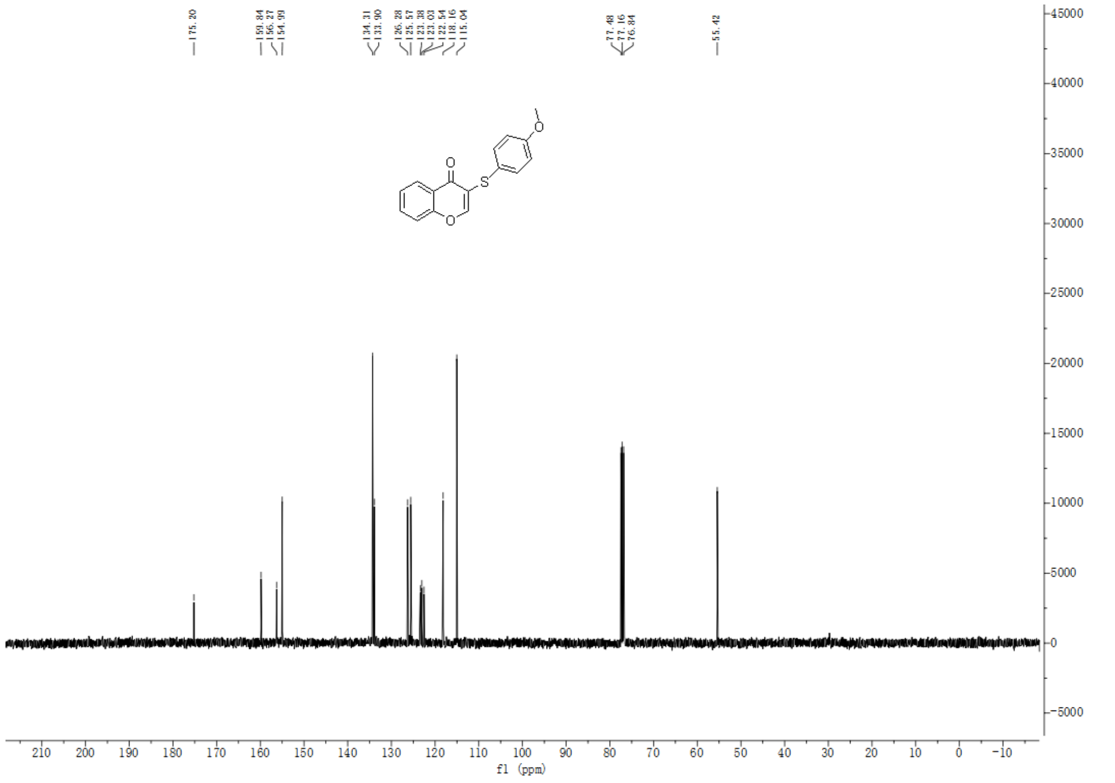

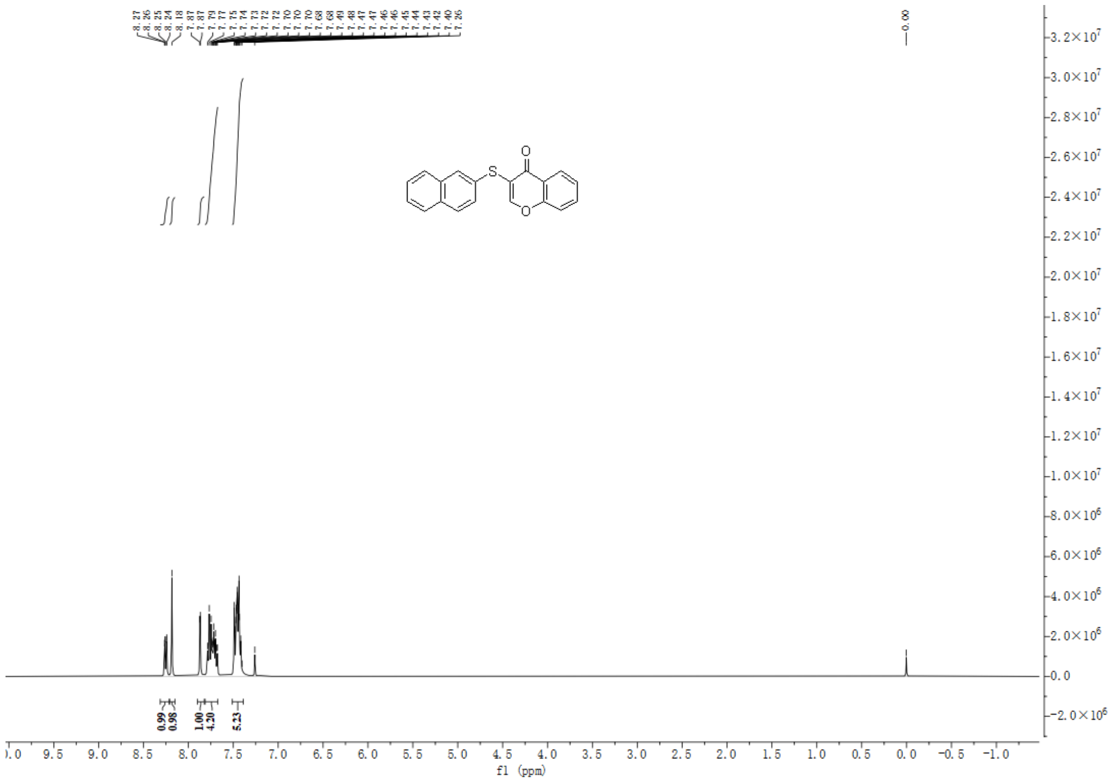

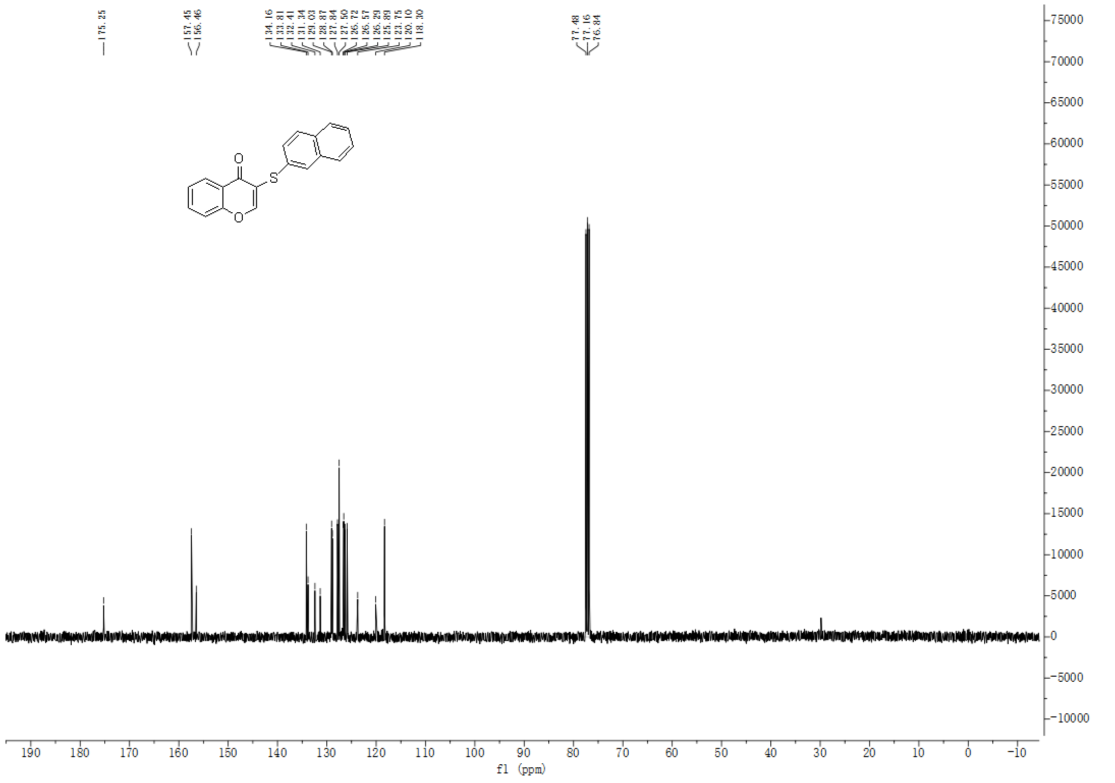

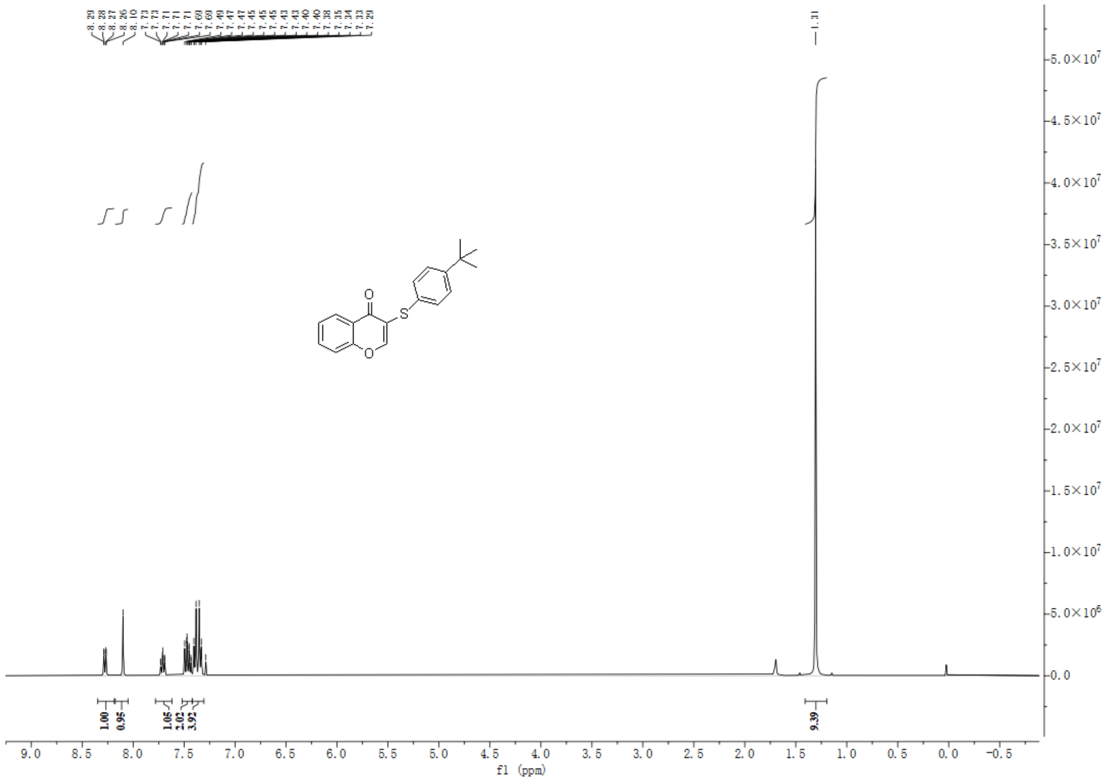

8.29
8.28
8.27
8.26
8.10
7.73
7.73
7.71
7.71
7.71
7.69
7.69
7.49
7.47
7.47
7.45
7.45
7.45
7.43
7.43
7.40
7.40
7.38
7.35
7.34
7.33
7.29
1.31
1.00
0.95
1.05
2.02
3.92
9.39
S
O
O
f1 (ppm)

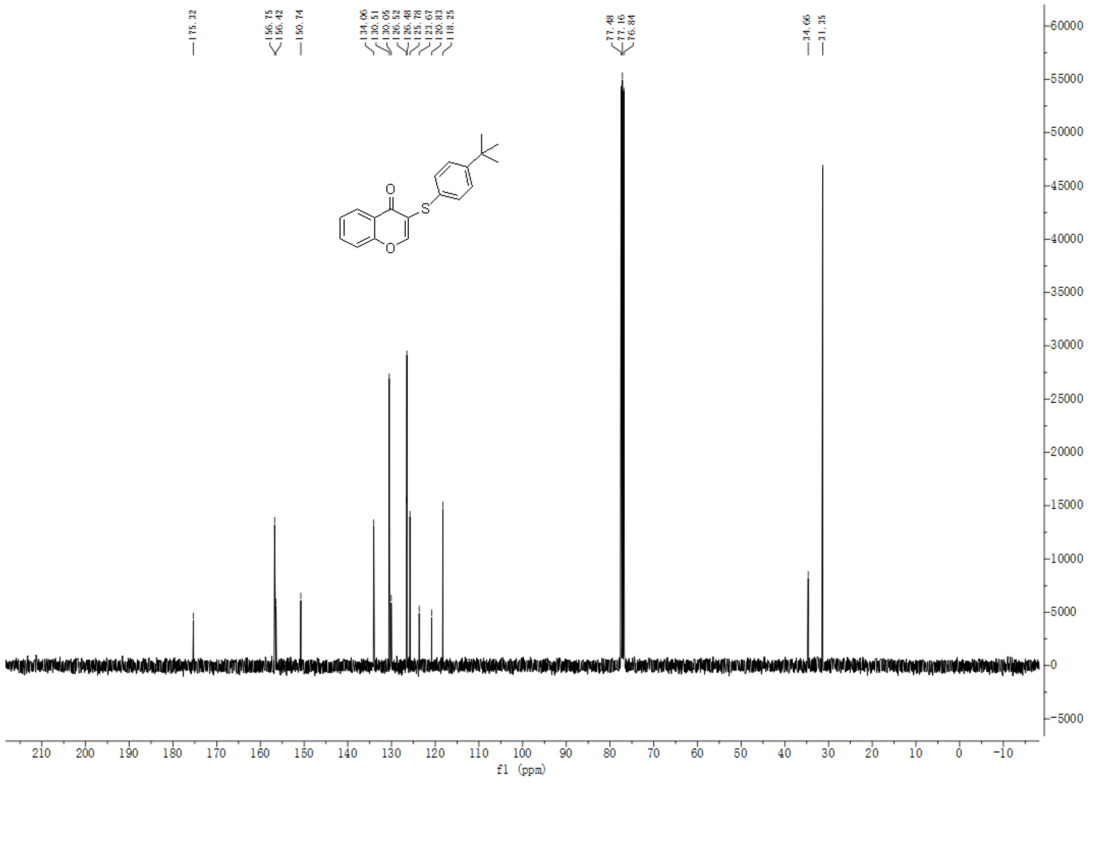

175.32
156.75
156.42
150.74
134.06
130.51
130.05
126.52
126.48
125.78
123.67
120.83
118.25
77.48
77.16
76.84
34.66
31.35
S
O
O
f1 (ppm)

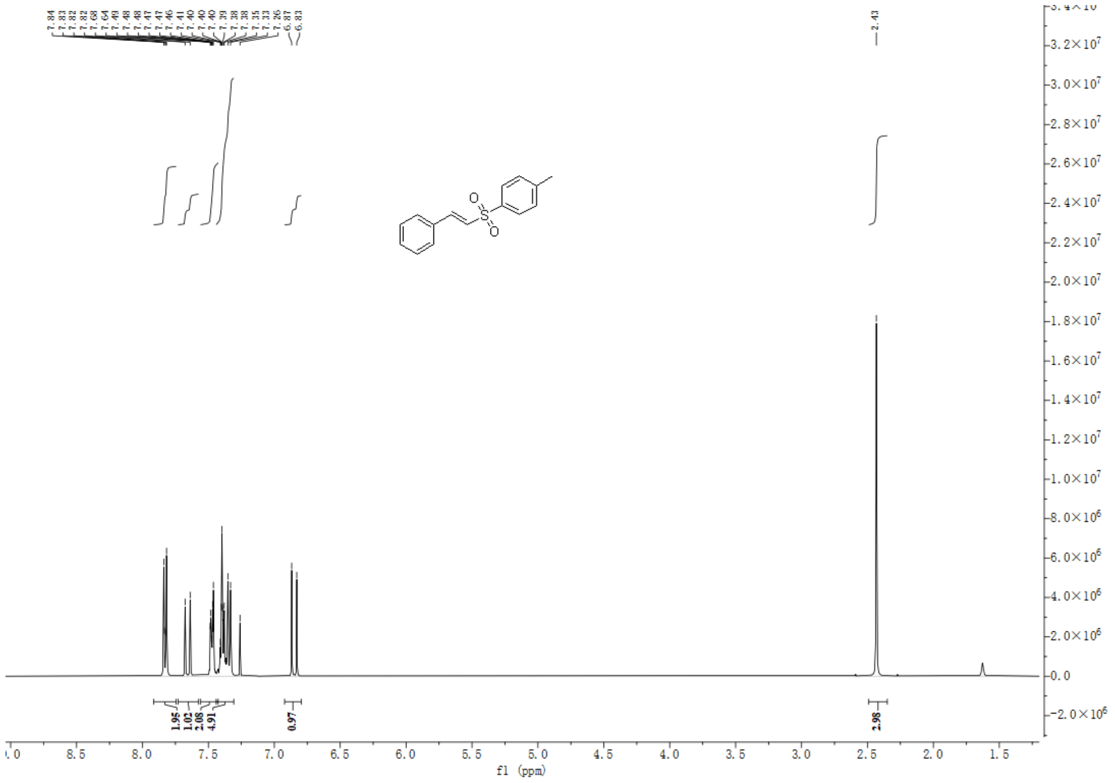

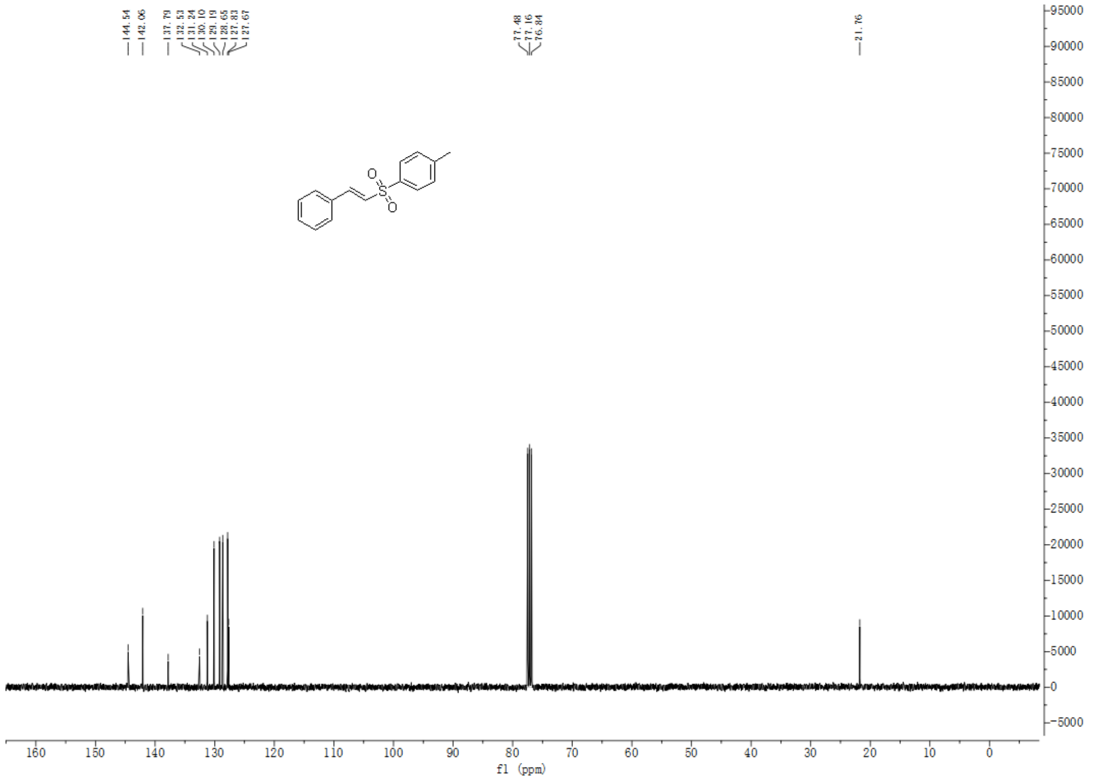

Supplement: File 1 — Experimental procedures, compound characterization data, and copies of NMR spectra. [file Beilstein_J_Org_Chem-21-253-s001.pdf]
